# Diverse effects of a *Cyperus rotundus* extract on glucose uptake in myotubes and adipocytes and its suppression on adipocyte maturation

**DOI:** 10.1038/s41598-024-59357-0

**Published:** 2024-04-19

**Authors:** Vipawee Pichetkun, Hnin Ei Ei Khine, Suchada Srifa, Sasiwimon Nukulkit, Nitra Nuengchamnong, Supakarn Hansapaiboon, Rattaporn Saenmuangchin, Chatchai Chaotham, Chaisak Chansriniyom

**Affiliations:** 1 Pharmaceutical Sciences and Technology Program, Faculty of Pharmaceutical Sciences, Chulalongkorn University, Bangkok, 10330 Thailand; 2 Center of Excellence in Natural Products and Nanoparticles (NP2), Faculty of Pharmaceutical Sciences, Chulalongkorn University, Bangkok, 10330 Thailand; 3 Department of Biochemistry and Microbiology, Faculty of Pharmaceutical Sciences, Chulalongkorn University, Bangkok, 10330 Thailand; 4 Preclinical Toxicity and Efficacy Assessment of Medicines and Chemicals Research Unit, Faculty of Pharmaceutical Sciences, Chulalongkorn University, Bangkok, 10330 Thailand; 5 Science Laboratory Center, Faculty of Science, Naresuan University, Phitsanulok, 65000 Thailand; 6 Pharmaceutical Research Instrument Center of the Faculty of Pharmaceutical Sciences, Chulalongkorn University, Bangkok, 10330 Thailand; 7 National Nanotechnology Center, National Science and Technology Development Agency, 111 Phahonyothin Rd., Klongluang, Pathumthani, 12120 Thailand; 8 Department of Pharmacognosy and Pharmaceutical Botany, Faculty of Pharmaceutical Sciences, Chulalongkorn University, Bangkok, 10330 Thailand

**Keywords:** *Cyperus rotundus*, Glucose transport, Adipogenesis, L6 myotubes, 3T3-L1 adipocytes, Cell biology, Molecular biology, Endocrinology

## Abstract

*Cyperus rotundus* rhizomes have been used in longevity remedies in Thailand for nourishing good health, which led us to investigate the effect on energy homeostasis, especially glucose utilization in myotubes and adipocytes, and on inhibition of lipogenesis in adipocytes. The results showed that an ethyl acetate extract of *C. rotundus* rhizomes (ECR) containing 1.61%w/w piceatannol, with a half-maximal concentration of 17.76 ± 0.03 μg/mL in 2,2-diphenyl-1-picrylhydrazyl (DPPH) radical scavenging assay, caused upregulation and cell-membrane translocation of glucose transporters GLUT4 and 1 in L6 myotubes but downregulation and cytoplasmic localization of GLUT4 expression in 3T3-L1 adipocytes and was related to the p-Akt/Akt ratio in both cells, especially at 100 μg/mL. Moreover, ECR (25–100 μg/mL) significantly inhibited lipid accumulation via Adenosine Monophosphate-Activated Protein Kinase (AMPK), Acetyl CoA Carboxylase (ACC), and Glycogen Synthase Kinase (GSK) pathways. Its immunoblot showed increased expression of p-AMPKα/AMPKα and p-ACC/ACC but decreased expression of p-Akt/Akt and p-GSK3β/GSK3β in 3T3-L1 adipocytes. Moreover, the decreased expression of the adipogenic effectors, perilipin1 and lipoprotein lipase, in ECR-incubated adipocytes (50 and 100 μg/mL) indicated reduced de novo lipogenesis. Our study elucidated mechanisms of *C. rotundus* that help attenuate glucose tolerance in skeletal muscle and inhibit lipid droplet accumulation in adipose tissue.

## Introduction

Metabolic syndrome (MetS) with Type 2 diabetes (T2DM) increases the risk of cardiovascular and cerebrovascular diseases, which can be induced by insulin resistance^1^. Impaired glucose tolerance, a consequence of insulin resistance, promotes development of T2DM. Additionally, obesity is a risk factor for T2DM, and insulin resistance is related to body mass index^2^. Glucose and lipid metabolism are critical processes in energy homeostasis. Especially, glucose is a starting material in glycolysis, and its product, acetyl CoA, enters the de novo lipogenesis pathway and is catalyzed by acetyl CoA carboxylase (ACC), generating fatty acid (FA) and triglyceride (TG), subsequently forming lipid droplets in adipocytes and liver cells^3^. Glucose metabolism in skeletal muscle, adipose tissue, and liver is controlled by insulin via the phosphoinositide 3-kinase (PI3K)/protein kinase B (Akt) pathway. Glucose transporters (GLUTs), especially the facilitative GLUT family, mediate cellular glucose uptake^4^. Thus, increasing glucose utilization by peripheral tissues, especially body muscles, and limiting glucose and lipid uptake in adipose tissue would benefit individuals who are pre-diabetic or diabetic. Additionally, suppression of adipocytes in lipogenesis can have a therapeutic benefit in obesity. Thus, mechanistic proteins in energy homeostasis pathways and inter-organ crosstalk were targeted and investigated.

To search for a cure, traditional practitioners have used herbal remedies to treat hyperglycemia. However, evidence is needed to prove their therapeutic outcomes, mechanism of action, and quality control. Much research has focused on plant compounds as leads in drug development. In previous studies on adipogenesis, we reported pinostrobin and 4,5,4′-trihydroxy-3,3′-dimethoxybibenzyl as adipogenic suppressors^5,6^. PI3K/Akt, AMP kinase (AMPK), and glycogen synthase kinase-3 β (GSK3β) pathways are our targets of interest because many natural compounds, such as maackiain, ononin, pinostrobin, and 4,5,4′-trihydroxy-3,3′-dimethoxybibenzyl, modulate energy homeostasis in these pathways, resulting in inhibition of adipogenic differentiation and lipid-droplet accumulation^7^.

*Cyperus rotundus* L. (Cyperaceae) is a weedy plant distributed in tropical and subtropical Afro-Eurasia, America, and Australia. A characteristic of *C. rotundus* is its aromatic rhizome, which has been used in traditional medicine; for example, in India, it has been used to treat dysentery and pain^8^; in China, it relieves pain and dysmenorrhea and regulates liver qi^9^; and in Thai traditional medicine, it is used to treat gastrointestinal disturbance. The *C. rotundus* rhizome is also used in longevity remedies for increasing physical strength and nourishing the body^10^. For, pharmacological studies, it exhibited antidiabetic activity and anti-obesity effects. For example, *C. rotundus* rhizome extract alleviated hyperglycemia in alloxan-induced diabetic rats^8^ and streptozotocin-induced diabetic mice^11^. Additionally, the extract improved the lipid profile in diabetic mice^11^. An extract of *C. rotundus* rihizome suppressed adipocyte differentiation through lowering the expression of peroxisome proliferators-activated receptor γ (PPARγ) and adipocyte protein 2 (aP2) genes, lowered weight gain in obese mice, and improved lipid profile and body mass index (BMI) in obese and dyslipidemia volunteers^12^.

As many studies of *C. rotundus* reported the effect on metabolic changes in animals and anti-adipogenic activity in pre-adipocytes, our study aim was to investigate the effect of *C. rotundus* extract on modulating glucose uptake in myotubes and mature adipocytes, which is a strategic action for decreasing glucose tolerance and more related to T2DM. In addition, To expand the therapeutic application of *C. rotundus* for T2DM caused mainly by obesity, we evaluated the effect of *C. rotundus* extract on lipid accumulation in mature adipocytes in this study.

## Results

### Anti-radical activities of *C. rotundus* extracts determined using DPPH radical scavenging assay

Among the tested extracts of *C. rotundus*, the ethyl acetate extract of *C. rotundus* rhizomes (ECR) exhibited the highest anti-radical scavenging activity, with a half-maximal concentration (IC_50_) of 17.76 ± 0.01 μg/mL. The crude methanol, aqueous, and hexane extracts had lesser activities, with IC_50_ values of 98.24 ± 1.09, 214.02 ± 0.91, and 1032.03 ± 8.00 μg/mL, respectively. Ascorbic acid was used as a positive control (IC_50_ of 3.20 ± 0.01 μg/mL).

### Quantification of piceatannol in ECR using HPLC and phytochemical analysis using tandem mass spectrometry

**Figure 1:**
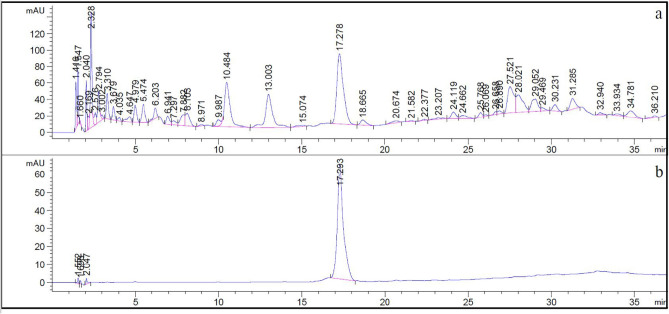
LC-DAD chromatograms of ECR (5 mg/mL) (**a**) and piceatannol (60 μg/mL, *t*_r_ = 17.293 min) (**b**) at 300 nm.

To standardize the ECR extract, we used our isolated compound, piceatannol, as a chemical marker. The ECR extract contained 1.61 ± 0.01%w/w piceatannol. The chromatograms of ECR and piceatannol (*t*_r_ = 17.29 min) are shown in Fig. 1. The regression equation was established in a range of 40–120 μg/mL as follows: $Y = 32.332x - 115.98$ (*R*^*2*^ = 0.9998), where Y is the peak area, x is the concentration (μg/mL), and *R*^*2*^ is the coefficient of determination (Figs. S1.1–S1.2).

In addition, the accuracy and precision were reported as %recovery and %CV (coefficient of variation), which were 98.67–107.97%, and < 1.1%, respectively. The limit of detection (LOD) and limit of quantitation (LOQ) in the piceatannol analytical method were 1.7 and 5.6 μg/mL, respectively.

In gas chromatography tandem mass spectrometry (GC–MS/MS) analysis, the volatile substances were mostly sesquiterpenoids, such as cis-Z-α-bisabolene epoxide, caryophyllene epoxide, corymbolone, cadelene, and lactaropallidin were revealed (Figs. S2, S2.1–S2.15, and Table S1). Additionally, the negative-ionization mode of liquid chromatography tandem mass spectrometry (LC–ESI–MS/QTOF-MS) analysis (Figs. S3a, S3A.1–S3A.37, and Table S2) disclosed common metabolites in plants, such as hexose, quinic acid, glyceric acid, and oxoglutaric acid, were identified. In addition, hydroxybenzene derivatives, i.e., gallic acid, isovanillic acid, vanillic acid, protocatechuic acid, syringic acid, and 3,4-dihydroxybenzaldehyde, were identified in the extract. Phenylpropanoid derivatives [caffeoylquinic acid, feruloylquinic acid, and *p*-coumaric acid], flavonoids [tricetin and kaempferol], a chalcone glycoside [nothofagin], and stilbenoids [piceatannol, scirpusins A and B] were detected in the ECR. Piceatannol, a 3′-hydroxy resveratrol, showed the characteristic fragmentation ion peaks [225.0551, 201.0553, 173.0607, and 159.0455] caused by the collision induced-deprotonation reaction of the catechol group^13^. Scirpusin A, *m/z* 515.1346 [M + HCOO]^−^, was assigned on the basis of the proposed fragmentation ions of 385.1054 (C_24_H_17_O_5_^−^), 359.0985 (C_22_H_15_O_5_^−^), 346.0822 (C_21_H_14_O_5_^•−^), and 241.0523 (C_14_H_9_O_4_^−^) (see Supplementary Information, S3A.30). At 16 mass units more than scirpusin A, the scirpusin B signal was observed at the retention time (*t*_r_) of 16.49 min with MS/MS fragments of 375.0868 (C_22_H_15_O_6_^−^), 241.0504 (C_14_H_9_O_4_^2•−^), 135.0451 (C_8_H_7_O_2_^−^), and 109.0296 (C_6_H_5_O_2_^−^) (see Supplementary Information, S3A.27). For the positive-ionization detection (Figs. S3b, S3B.1–S3B.30, and Table S3), small nitrogen-containing compounds, such as (*R*)-5-aminopentan-2-ol, 3-aminobenzene-1,2-diol, 1-methyl-2-pyrrole carboxaldehyde, and 4-acetamidobutanoic acid, were identified. Rotundine A, a sesquiterpene alkaloid isolated from this plant^14^, was detected. In addition, sesquiterpenes, such as procurcumenol and curcumene, were proposed. Moreover, some phenolic compounds, such as maltol and kaempferol, were also identified.

### Effect of ECR on cell viability and glucose consumption in L6 myotubes 3T3-L1 adipocytes

**Figure 2:**
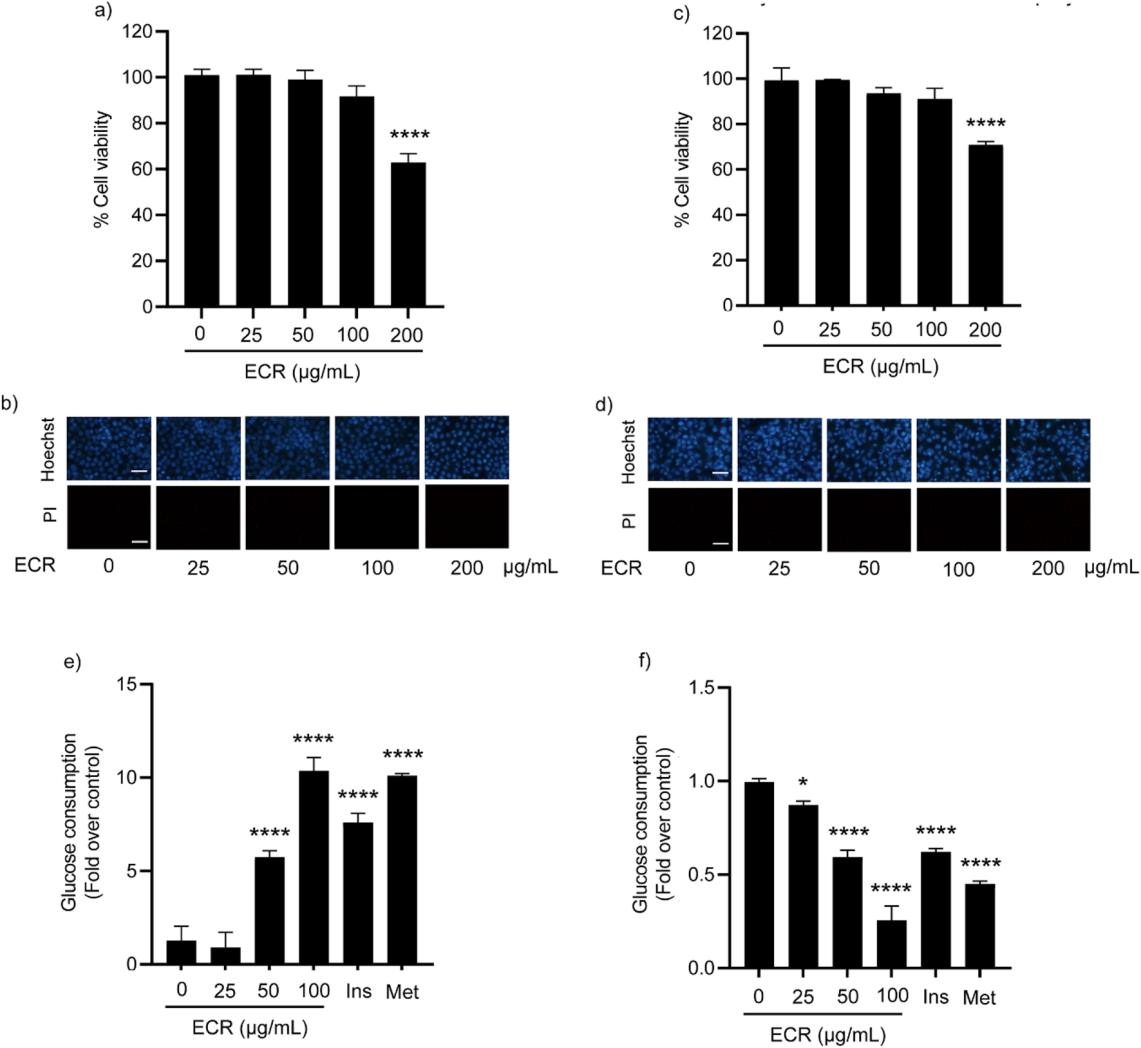
Effects of ECR on cell viability and glucose consumption in L6 and 3T3-L1 cells. MTT viability studies of ECR in L6 myotubes (**a**) and 3T3-L1 adipocytes (**c**) after 48 h of exposure. L6 myotubes (**b)** and 3T3-L1 adipocytes (**d**) were stained with Hoechst33342 and propidium iodide (PI) and photographed using a fluorescence microscope. Glucose consumption in L6 myotubes (**e**) and 3T3-L1 adipocytes (**f**) was incubated with non-toxic concentrations of ECR (25–100 μg/mL). Insulin (Ins, 100 nM) and metformin (Met, 1 mM) were used as positive controls. The error bars represented ± SEM of triplicate data. *p < 0.05, **p < 0.01, ***p < 0.001, ****p < 0.0001; statistical significance compared to the control. ECR; ethyl acetate extract of *C. rotundus* rhizomes.

L6 myotubes and 3T3-L1 adipocytes were incubated with ECR at concentrations from 25 to 200 μg/mL for 48 h; cell viability was subsequently determined by MTT assay. The cell viability was significantly decreased at 200 μg/mL in both cells relative to the untreated control group (Fig. 2a and c). Hoechst33342/PI co-staining revealed no cell death in the range of 25–100 μg/mL ECR (Fig. 2b and d). In contrast, 200 μg/mL ECR-treated cells showed bright blue Hoechst33342 fluorescence due to condensed chromatin and/or DNA fragmentation, indicating a toxic dose at 200 μg/mL. Cell necrosis was not suggested, as no red PI fluorescence was observed. Thus, the non-toxic concentrations of 25–100 μg/mL were used in further experiments.

A glucose assay kit was used to measure the glucose consumption in the ECR-treated cells. After treating the L6 myotubes either with ECR (50–100 μg/mL), insulin (100 nM), or metformin (1 mM) for 48 h, the remaining glucose in the culture media was decreased relative to that in the untreated control, suggesting that ECR from 50 and 100 μg/mL stimulated glucose uptake in L6 myotubes. Notably, the ECR effect at 100 μg/mL on stimulating glucose uptake in L6 cells was comparable to that of 1 mM metformin (Fig. 2e). The enhancement of glucose uptake activity of ECR in L6 myotubes depended on the extract concentration.

The effect of ECR on glucose consumption was diverse in 3T3-L1 adipocytes from in L6 myotubes. The glucose uptake in 3T3-L1 adipocyte cells showed a significant dose-dependent decrease with increases in the remaining glucose in the media. On the other hand, in 3T3-L1 cells, ECR at 100 μg/mL had a higher inhibitory effect on glucose consumption than insulin (100 nM), and metformin (1 mM).

### Effect of ECR on the expression and localization of glucose transporters in L6 myotubes and 3T3-L1 adipocytes

**Figure 3:**
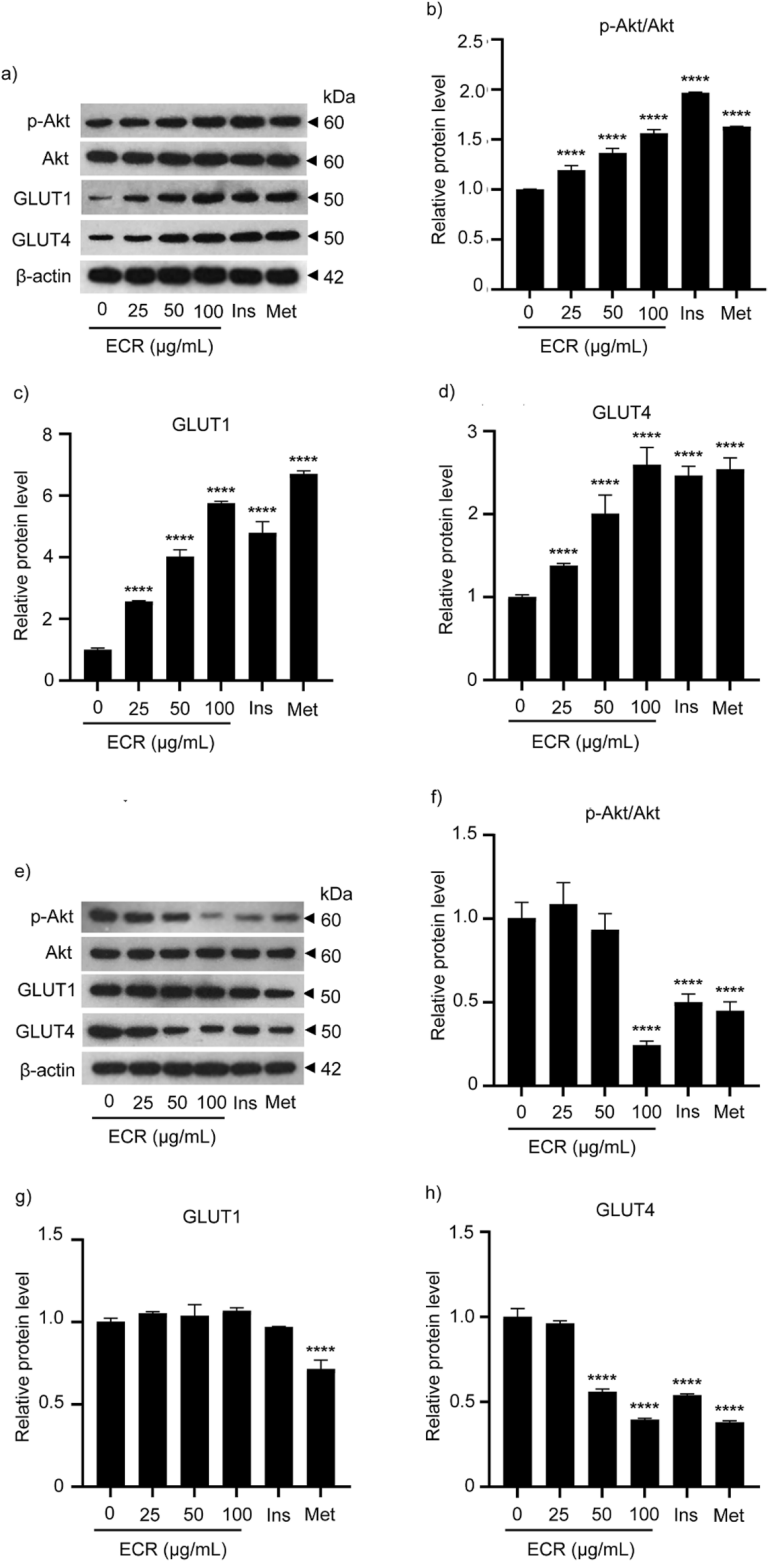
Effect of ECR on the expression of glucose transporters and Akt signaling protein in L6 and 3T3-L1 cells. Western blotting demonstrated the expression of p-Akt, Akt, GLUT1, GLUT4, and β-actin in L6 myotubes (**a**) and 3T3-L1 adipocytes (**e**) incubated with ECR at concentrations of 25, 50, and 100 μg/mL for 48 h. The relative protein levels of p-Akt/Akt (**b**, L6 cells; **f**, 3T3-L1 cells), GLUT1 (**c**, L6 cells; **g**, 3T3-L1 cells), and GLUT4 (**d**, L6 cells; **h**, 3T3-L1 cells) were determined using ImageJ program and normalized against β-actin level. Insulin (Ins, 100 nM) and metformin (Met, 1 mM) were used as positive controls. The error bars represented ± SEM of triplicate data. *p < 0.05, **p < 0.01, ***p < 0.001, ****p < 0.0001; statistical significance compared to the control.

To further investigate the effects of ECR on GLUTs in L6 and 3T3-L1 cells, western blot analysis was performed to examine the expression levels of p-Akt/Akt and facilitative GLUT1 and 4. The ECR-treated L6 myotubes showed a significant increase in GLUT1 and 4 expression in a dose-dependent manner. Interestingly, ECR at 100 μg/mL stimulated GLUT4 expression as potently as insulin and metformin (Fig. 3a, c, and d). The rise in the level of phosphorylated-Akt/Akt (p-Akt/Akt), a signaling element of GLUT4 translocation, in the ECR-treated cells was also observed in L6 cells (Fig. 3b). In contrast, GLUT4 expression in 3T3-L1 cells decreased, consistent with p-Akt/Akt, especially at 100 μg/mL ECR (Fig. 3e, f, and h). However, GLUT1 in 3T3-L1 adipocytes exhibited a relatively unchanged expression level even at 100 μg/mL ECR (Fig. 3g).

**Figure 4:**
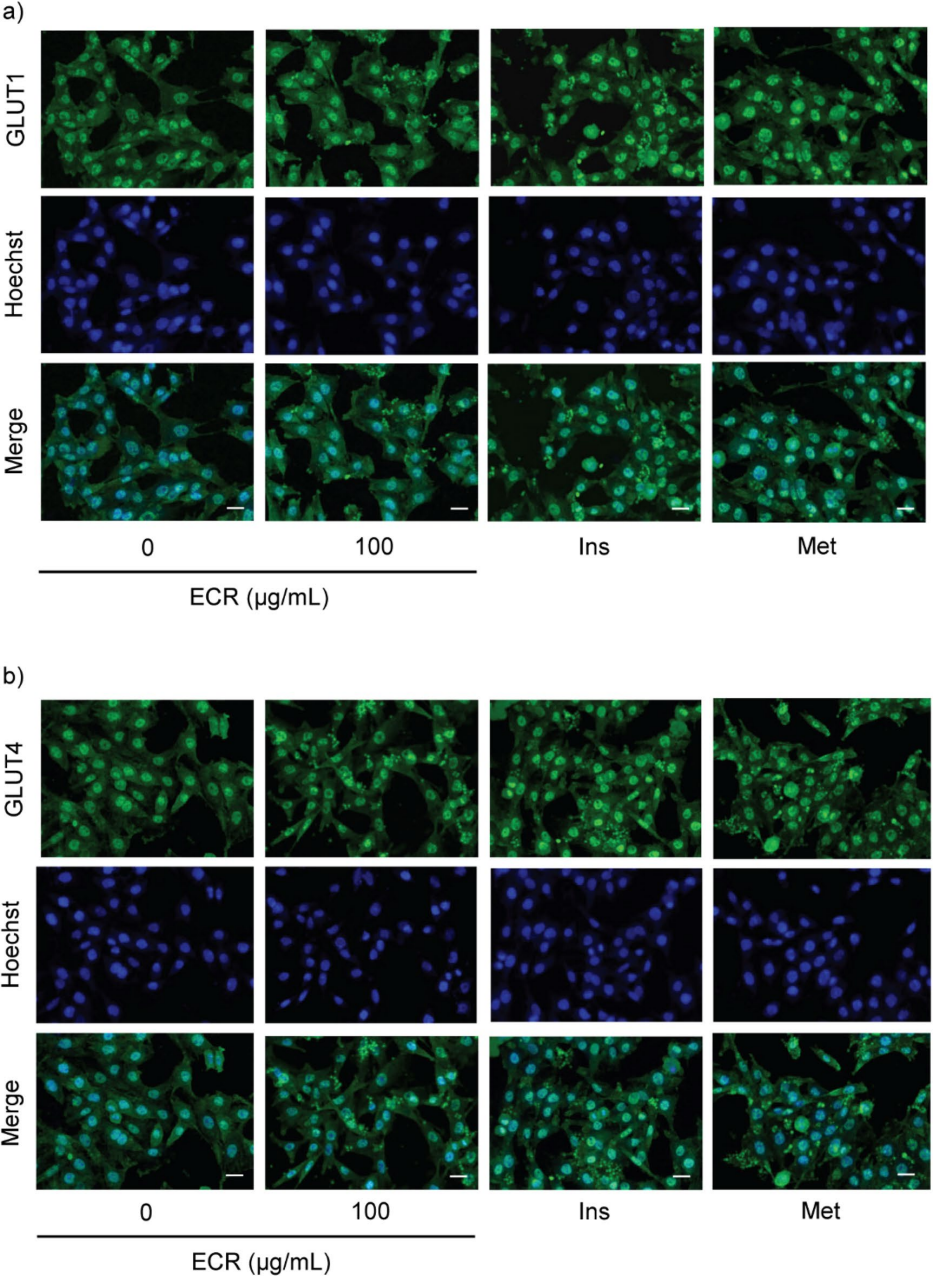
Effect of ECR on translocation of glucose transporters in L6 myotubes. Immunofluorescence represented GLUT1 and 4 on L6 myotubes (**a**, GLUT1; **b** GLUT4) incubated with ECR at a concentration of 100 μg/mL for 48 h. The GLUT1 and 4 were labeled with green fluorescence from specific secondary antibodies. The nucleus of the cells was labeled in blue using Hoechst33342. The merged picture illustrated the locations of the nucleus and GLUT. The images were magnified at × 20 under a confocal microscope (scale bar = 20 μm). Insulin (Ins, 100 nM) and metformin (Met, 1 mM) were used as positive controls.

**Figure 5:**
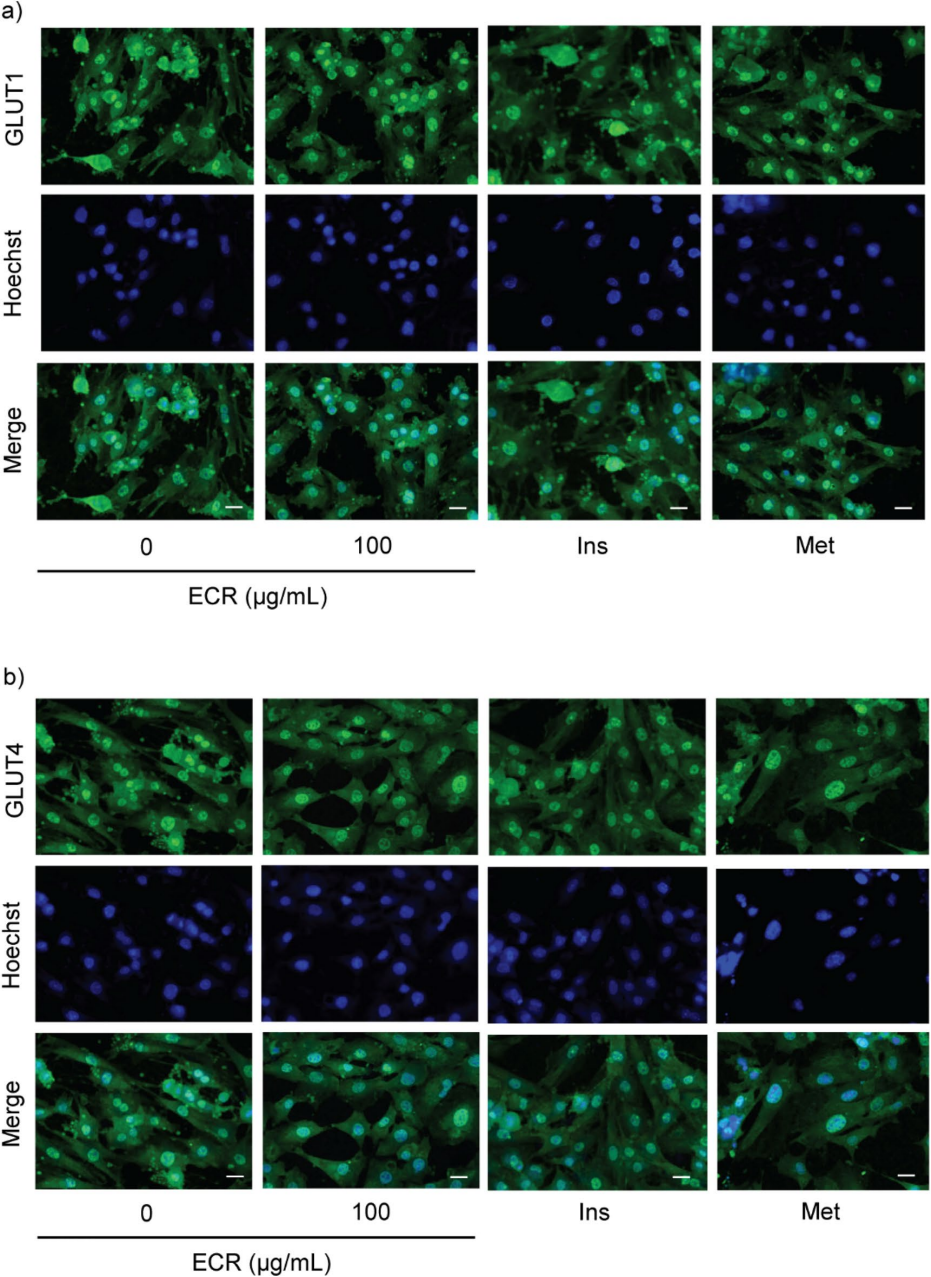
Effect of ECR on translocation of glucose transporters in 3T3-L1 adipocytes. Immunofluorescence represented GLUT1 and 4 on 3T3-L1 adipocytes (**a**, GLUT1; **b** GLUT4) incubated with ECR at a concentration of 100 μg/mL for 48 h. The GLUT1 and 4 were labeled with green fluorescence from specific secondary antibodies. The nucleus of the cells was labeled in blue using Hoechst33342. The merged picture illustrated the locations of the nucleus and GLUT. The images were magnified at × 20 under a confocal microscope (scale bar = 20 μm). Insulin (Ins, 100 nM) and metformin (Met, 1 mM) were used as positive controls.

To confirm the effect of ECR on GLUT translocation in both cell types, we performed an immunofluorescence-labeling assay. At 100 μg/mL, ECR enhanced the plasma membrane translocation of GLUT1 and 4 in L6 cells in a manner similar to insulin and metformin (Fig. 4a and b). Inversely, ECR (100 μg/mL) decreased the translocation to cell membranes of GLUT4 in 3T3-L1 cells (Fig. 5a and b). This effect was similar to those of insulin and metformin. However, compared with the control group, ECR did not have a significant effect on GLUT1 localization. Notably, metformin decreased GLUT1 translocation. In 3T3-L1 cells, GLUT4 showed a greater diminished translocation than GLUT1. The observed translocation patterns of GLUT were consistent with the expression of GLUT in previous experiments.

### Suppressive effect of ECR on lipid accumulation in 3T3-L1 adipocytes

**Figure 6:**
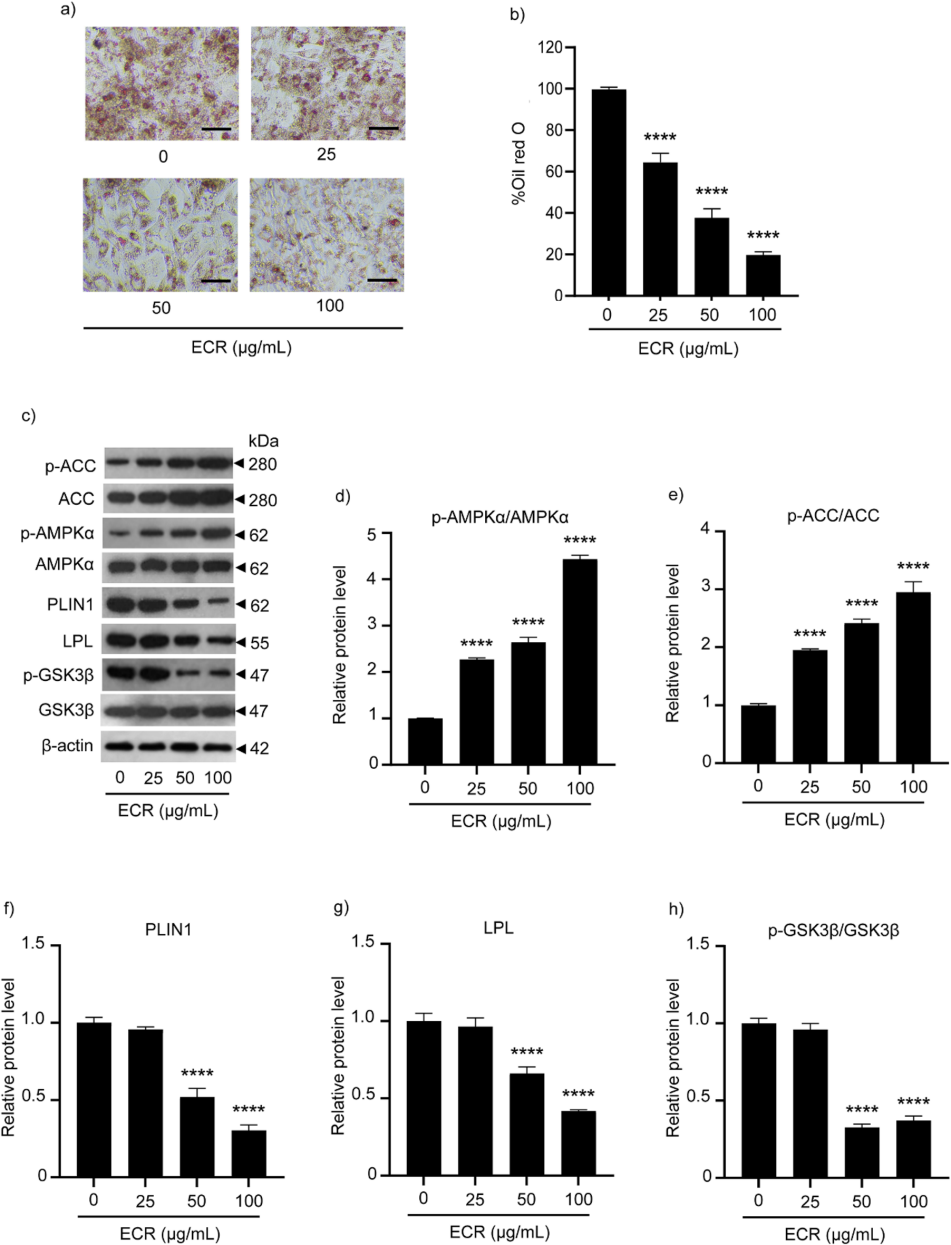
Suppressive effect of ECR on adipocyte maturation in 3T3-L1 cells. Images represented Oil Red O (ORO) staining for lipid droplets in 3T3-L1 adipocytes incubated with ECR at concentrations of 25, 50, and 100 μg/mL for 48 h. (**a**). Percentage of ORO staining existed in 3T3-L1 adipocytes (**b**). Western blotting demonstrated the expression of p-ACC, ACC, p-AMPKα, AMPKα, PLIN1, LPL, p-GSK3β, GSK3β in 3T3-L1 adipocytes (**c**). The relative protein levels of p-AMPKα/AMPKα (**d**), p-ACC/ACC (**e**), PLIN1 (**f**), LPL (**g**), p-GSK3β/GSK3β (**h**) were determined using ImageJ program and normalized against β-actin level. The error bars represented ± SEM of triplicate data. *p < 0.05, **p < 0.01, ***p < 0.001, ****p < 0.0001; statistical significance compared to the control.

The metabolic effect of ECR was further investigated in mature adipocytes. Oil Red O (ORO) staining was used in a lipid-accumulation study. The ECR (25–100 μg/mL) significantly inhibited the cellular lipid droplet accumulation dose-dependently (Fig. 6a). %Oil red O in ECR-treated cells was reduced relative to that in the untreated control group (Fig. 6b). The decreased accumulation of lipid droplets could result from inhibition of lipogenesis. Therefore, we determined the level of corresponding proteins in the lipogenesis pathway. Culture with ECR (25–100 μg/mL) increased the ratios of p-AMPKα/AMPKα and p-ACC/ACC in 3T3-L1 adipocytes (Fig. 6c–e). Additionally, the ECR at 50 and 100 μg/mL decreased the p-GSK3β/GSK3β level (Fig. 6h). On the basis of these findings, we hypothesized that ECR at 100 μg/mL suppresses the Akt/AMPK/ACC and Akt/GSK3β signaling pathways. Furthermore, ECR at concentrations of 50 and 100 μg/mL caused significant downregulation of two adipogenic effector proteins, perilipin1 (PLIN1) and lipoprotein lipase (LPL) (Fig. 6f and g), indicating reduced fatty acid uptake and accumulation.

## Discussion

People developing MetS are advised to modify their lifestyle, and most use dietary supplements or traditional medicine before standard medical treatment. In Thai traditional medicine, *C. rotundus* rhizomes have been used with other herbs in a longevity remedy to restore good health and maintain body strength^10^. As energy balance is involved in the pathogenesis of MetS and carbohydrate metabolism^15^, we thought that *C. rotundus* would maintain the body’s energy homeostasis consistent with its traditional use. Hydroethanolic extracts of *C. rotundus* showed an anti-hyperglycemia effect in rats with alloxan-induced diabetes and anti-protein glycoxidation properties by effectively suppressing oxidative processes. The observed antiglycation and anti-oxidant properties of *C. rotundus* may be attributed to its phenolic content^16^. Accordingly, it is evident that *C. rotundus* extract displaying the anti-oxidant activity can influence anti-diabetic activity and relieve its complications. In this paper, we reported the effects of anti-radical ECR extract on glucose uptake in glucose disposal cells—L6 myotubes and 3T3-L1 adipocytes—and its repression on adipocyte maturation.

The crude methanolic extract and its partitioned solvent extracts were tested for anti- DPPH radical activity. The ECR had the highest anti-radical activity among the extracts. Since anti-radical activity benefits metabolic processes against the overproduction of free radicals^17^, we selected ECR for further investigation. In addition, several studies have shown that anti-oxidant activity is related to anti-diabetic activity and anti-obesity^18^. The chemical constituents of ECR were revealed by GC–MS/MS and LC–MS/MS and showed that sesquiterpenes and phenolics (Tables S1–S3) were present. The ECR phenolics, such as piceatannol and scirpusins A and B, are members of stilbenoids, reported as standardized markers exhibiting anti-obesity activity in *C. rotundus* extracts^12^. In our research, piceatannol was successfully isolated from the ECR and used for quantification. The ECR used in our experiments contained 1.61%w/w piceatannol by HPLC. Unfortunately, due to its isolated amounts, the pure compound was not tested along with the ECR in this study. Piceatannol reportedly lowered blood glucose in diabetic mice and promoted GLUT4 translocation in L6 myotubes^19^. Moreover, piceatannol exhibited an anti-obesity effect in estrogen-deficient female mice and C57BL/6 mice^20^. Furthermore, piceatannol inhibited lipid accumulation, decreased glucose uptake, and suppressed adipocyte differentiation in human mesenchymal stem cell (hMSC)–adipocytes^21^. Additionally, other phenolics in the ECR emphasized the shikimate pathway influenced by the versatility of compounds in *C. rotundus* rhizomes, which may exert the anti-oxidant and anti-diabetic activities of the extract.

We hypothesized that the physical-strength-enhancing effect of *C. rotundus* rhizomes is related to the regulation of glucose consumption. Thus, we examined the effects of ECR on glucose uptake in myotubes and adipocytes. The glucose uptakes at 50 and 100 μg/mL ECR were 4.5- and eightfold higher, respectively, in L6 myotubes than the control, consistent with the response from insulin (100 nM) and metformin (1 mM) (Fig. 2e). Conversely, the glucose consumptions at 50 and 100 μg/mL ECR were 0.4- and 0.7-fold lower, respectively, in 3T3-L1 adipocytes than the control (Fig. 2f). These results imply that cell types had different modulatory effects on glucose uptake of ECR. Therefore, we further investigated the effect of ECR on various cellular GLUTs. Glucose metabolism in skeletal muscle and adipose tissue is influenced by insulin through the facilitative GLUT. Approximately 90% and 10% of postprandial glucose uptake is by skeletal muscle and adipose tissue, respectively^22^. Mechanistically, insulin initiates PI3K/Atk signaling pathway, leading to GLUT translocation to the cell membrane and raised glucose uptake. The increase in glucose consumption in ECR-treated L6 myotubes (Fig. 2e) was associated with the upregulated GLUT4 and 1 protein levels (Fig. 3c and d) and GLUT4 and 1 localization on cell membranes shown by immunofluorescence imaging (Fig. 4a and b). Interestingly, the expression was higher for GLUT1 than GLUT4 at ratios (GLUT1/GLUT4) of 1.87–2.22 for 25–100 μg/mL ECR, respectively. The ECR effect on GLUT4 and 1 expression in L6 cells corresponded to the metformin and insulin effects on GLUT expression, consistent with Sarabia et al.^23^. In contrast, diminishing glucose consumption in ECR-treated 3T3-L1 adipocytes was reflected in the decreased expression of GLUT4 with unchanged GLUT1 (Fig. 3g and h), corresponding to the reduced translocated GLUT4 on plasma membranes (Fig. 5b). It should be noted that the prolonged treatment (~ 24 to 48 h) of insulin and metformin could reduce p-Akt/Akt-mediated glucose transporter consequence with diminished glucose uptake in adipocytes^24,25^. In ECR-treated cells, expression of phosphorylated-Akt/Akt (p-Akt/Akt) showed the same trend as the expression of GLUT4 and 1 in L6 myotubes, whereas only suppression of GLUT4 expression in 3T3-L1 at 100 μg/mL ECR corresponded to the decreasing p-Akt/Akt protein, albeit with unchanged GLUT1 (Fig. 3f–h). This finding suggested that expression and translocation of GLUT4 and 1 in myotubes are facilitated by signaling via the insulin/Akt pathway^26^, whereas only GLUT4 in adipocytes is mainly regulated by the insulin/Akt pathway^4^. Recent research by Beg et al.^27^ reported that lipolysis regulated by adipose triglyceride lipase (ATGL)–cAMP–thioredoxin-interacting protein (TXN1P) axis most influenced the glucose uptake and surface localization of GLUT1 in 3T3-L1 adipocytes, supporting our result of unchanged GLUT1 expression and translocation in ECR-treated adipocytes (Figs. 3g and 5a). Our results suggested that ECR enhanced glucose utilization in myotubes and diminished glucose uptake in adipocytes, which would promote either glycolysis or glycogen synthesis in myotubes and reduce fatty acid accumulation and lipogenesis in adipocytes.

Downregulation of GLUT4 in ECR-treated 3T3-L1 cells prompted us to investigate lipogenesis. Cellular lipid accumulation assessed by ORO staining was observed to dose-dependently decrease in ECR-treated 3T3-L1 cells. By 80% reduction of ORO content in 100 μg/mL ECR-incubated cells, adipocyte maturation was suppressed by ECR (Fig. 6a and b). Mechanistically, protein expression in pathways related to lipogenesis was determined. The relative protein level of pT172-AMPKα/AMPKα was increased in 3T3-L1 adipocytes incubated with ECR, simultaneously increasing the ratio of p-ACC/ACC (Fig. 6d and e). In addition, ECR influenced the Akt/GS3Kβ signaling pathway leading to a significant decrease in p-GSK3β/GSK3β relative protein levels with a consistently diminishing ratio of p-Atk/Atk, especially at 100 μg/mL ECR (Fig. 6h). ECR activated the AMPK pathway by phosphorylation at Thr172 of its α-subunit along with promoting S79 phosphorylation of ACC. AMPK is activated when energy deprivation occurs, as shown by the ratio of AMP/ATP or ADP/ATP. The phosphorylated T172 of AMPK inhibits fatty acid synthesis by phosphorylation of the ACC residue, and the rate-limiting enzyme converts acetyl CoA to malonyl CoA, resulting in prevention of FA synthesis^28^. Additionally, AMPK is inhibited by insulin for FA synthesis. In this case, insulin-mediated Akt phosphorylates S485 of AMPK, subsequently increasing de-phosphorylation at S79 of ACC, leading to FA synthesis^29^. On the basis of our results in ECR-incubated 3T3-L1 cells, decreasing glucose consumption and GLUT4 expression resulted in a cellular fuel shortage. Susequently, AMPK is activated, and p-ACC is upregulated to inhibit FA synthesis, the ATP-consuming process. Moreover, the two adipocyte effectors, PLIN1 and LPL, decreased expression in ECR-treated 3T3-L1 cells (Fig. 6f and g), suggesting suppression of adipocyte maturation. PLIN1 is an integument protein in lipid droplets and regulates TG storage and lipolysis, whereas LPL is an enzyme synthesized by adipocytes and mediates lipoprotein hydrolysis and lipid uptake. PLIN1 and LPL are upregulated and encoded during adipocyte development^30,31^. We found that ECR influenced the Akt/GS3Kβ signaling pathway leading to a significant decrease in the p-GSK3β/GSK3β ratio with diminishing p-Atk/Atk, especially at 100 μg/mL ECR (Figs. 3f and 6h). GSK3β modulated various cellular processes, including adipogenesis. In adipocytes, GSK3β contrarily regulated sterol regulatory element binding protein 1c (SREBP1c). As previously shown, inactivation of GSK3β by phosphorylation at Ser9 by phosphorylated Akt led to SREBP1c activation and subsequent lipogenesis^32^. Our results showed that decreasing p-GSK3β/GSK3β might benefit anti-obesity. The GSK3 pathway plays crosstalk with the AMPK pathway, and both are influenced by PI3K/Akt signaling, mediated by insulin^33^. In addition, the previous studies also revealed the suppressive effect of *C. rotundus* extract and its’ constituents on adipocyte differentiation through modulating PPARγ and CCAAT/enhanced binding protein α (C/EBPα) expression^12,34–36^. Together with the results presented in this study, *C. rotundus* extract is potentially a novel anti-obesity therapy that inhibits both adipogenesis in pre-adipocytes and lipid accumulation in mature adipocytes.

## Materials and methods

### Plant material

The rhizomes of *Cyperus rotundus* were obtained from a farm in Kamphaeng Saen district, Nakhon Pathom Province, Thailand in September 2021, and identified by the one of authors (C. Chansriniyom). The herbarium specimen (CC-CR-050921) was deposited at the Department of Pharmacognosy and Pharmaceutical Botany, Faculty of Pharmaceutical Sciences, Chulalongkorn University. In addition, the handling and notification of *C. rotundus* rhizomes followed the Plant Variety Protection Act B.E. 2542 (1999): section 53, the Kingdom of Thailand. The study complied with the Thailand regulation mentioned above.

### Chemicals and reagents

Dulbecco’s Modified Eagle Medium (DMEM), fetal bovine serum (FBS), l-glutamine, antibiotic solution (Penicillin–Streptomycin; 10,000 U/mL), horse serum (HS), and trypsin were purchased from Gibco (Gaithersburg, MA, USA). Additionally, bovine serum albumin (BSA), dexamethasone, dimethyl sulfoxide (DMSO), ORO, isobutylmethylxanthine (IBMX), isopropanol, 2,2-diphenyl-1-picrylhydrazyl (DPPH), Glucose (GO) Assay Kit, Triton X-100, Hoechst33342, and propidium iodide (PI) were procured from Sigma-Aldrich (St. Louis, MO, USA). Insulin was sourced from Himedia (Mumbai, India), while bicinchoninic acid (BCA) protein assay kit, chemiluminescent western blot reagent (ECL substrates), radio-immunoprecipitation assay (RIPA) buffer, and 3-(4,5-dimethylthiazol-2-yl)-2,5-diphenyltetrazolium bromide (MTT) solution were acquired from Thermo-Fisher (Rockford, IL, USA). The protease inhibitor cocktail was obtained from Roche Applied Science (Indianapolis, IN, USA). Furthermore, primary antibodies against β-actin (Cat. #4970), Akt (Cat. #4691), p-Akt (Ser473) (Cat. #4060), GLUT1 (Cat. #12939), GLUT4 (Cat. #2213), GSK3β (Cat. #12456), p-GSK3β (Ser9) (Cat. #9322), AMPKα (Cat. #5831), p-AMPKα (Thr172) (Cat. #2535), ACC (Cat. #676), p-ACC (Ser79) (Cat. #11818), PLIN1 (Cat. #9349), and horseradish peroxidase (HRP)-linked secondary antibodies (Cat. #7074) were purchased from Cell Signaling Technology (Danvers, MA, USA), meanwhile LPL (Cat. #PA5-85126) was acquired from Invitrogen (Waltham, MA, USA).

### Extraction

The dried rhizomes (2.0 kg) were ground into small pieces and macerated with methanol (MeOH) to obtain the MeOH extract (286.8 g). Subsequently, the MeOH extract was partitioned with hexane to obtain a hexane extract (37.1 g). The MeOH extract was then added with distilled water and partitioned with ethyl acetate to yield an ethyl acetate extract (EtOAc, 22.9 g) and an aqueous extract (103.7 g).

The ethyl acetate extract (ECR) underwent separation using Medium-pressure liquid chromatography (MPLC) with silica gel as the stationary phase. The mobile phase consisted of varying ratios of hexane and EtOAc (80:20 to 0:100), resulting in 11 fractions (Fr.1-11). Fr.6 (4.26 g) was further purified in two more steps [silica gel, hexane–Me_2_CO (3:2 to 0:1); silica gel, CH_2_Cl_2_/Me_2_CO (9:1 to 8:2)], resulting in a pure compound (piceatannol, 4.0 mg). The spectroscopic data of isolated piceatannol was as follows. ^1^H NMR (MeOD, 400 MHz) δ: 6.16 (1H, t, *J* = 2 Hz, H-12), 6.43 (2H, d, *J* = 2 Hz, H-10, H-14), 6.74 (1H, d, *J* = 8 Hz, H-5), 6.74 (1H, d, *J* = 16 Hz, H-8), 6.83 (1H, dd, *J* = 8, 2 Hz, H-6), 6.89 (1H, d, *J* = 16 Hz, H-7), 6.97 (1H, d, *J* = 2 Hz, H-2). ^13^C NMR (MeOD, 400 MHz) δ: 102.8 (C-12), 105.9 (C-10, C-14), 113.9 (C-2), 116.6 (C-5), 120.3 (C-6), 127.1 (C-8), 129.8 (C-7), 131.2 (C-1), 141.4 (C-9), 146.6 (C-3, C-4), 159.8 (C-11, C-13). HR-ESI-MS m/z: 243.0664 [M-H]^−^ (Calculated for C_14_H_11_O_4_, 243.0663), Figs. S4–S10.

### DPPH radical scavenging assay

Anti-DPPH radical activity was conducted as described in Chansriniyom et al.^37^. In brief, 100 μL of sample was incubated with 100 μL of DPPH (100 μM) in the dark at 25 °C for 30 min. Then, the absorbance was measured at 517 nm by a microplate reader (Anthros, Durham, NC, USA). The DPPH scavenging activity (%) was determined as follows:

% DPPH scavenging activity = [(A_control_ − A_sample_)/ A_control_] × 100; where A_sample_ and A_control_ were the absorbances in the presence and absence of sample, respectively.

The 50% effective concentration (EC_50_) was calculated using a linear regression equation from the graph plotted between concentration and % scavenging activity.

### Quantification of piceatannol in ECR by high performance liquid chromatography (HPLC)

The quantification of piceatannol was performed on the OpenLab ChemStation system (Agilent Technology, CA, USA). The ECR was separated on ZORBAX Eclipse Plus C18, 5 μm, 4.6 × 150 mm column (Agilent Technology, CA, USA) at 30 °C. The HPLC condition, gradient elution programming, and operation setting were followed a study of Shao et al.^38^. Isolated piceatannol (purity > 95.0%) was used in this experiment. The analytical method parameters (linearity and range, accuracy, precision, LOD, and LOQ) were determined according to the ICH Q2(R1) guidelines.

### Cell cultures

#### L6 muscle-cell culture

L6 myoblasts were obtained from ATCC (Manassas, VA, USA) and cultured in DMEM supplemented with 10% FBS, 1% l-glutamine (200 mM), and 1% antibiotic solution under humidity and 5% CO_2_ at 37 °C. The L6 cells used were harvested after 70–80% confluence. For differentiation of L6 myoblasts to myotubes, the cells were cultured in DMEM supplemented with 2% HS, 1% l-glutamine (200 mM), and 1% antibiotic solution, with the media changed every 2 days until myotubes formation.

#### 3T3-L1 cell culture

Pre-adipocyte 3T3-L1 cells were obtained from ATCC (Manassas, VA, USA), cultured in DMEM supplemented with 10% FBS, 1% l-glutamine (200 mM), and 1% antibiotic solution (complete media), then incubated under humidity and 5% CO_2_ at 37 °C for 2 days. For differentiation of 3T3-L1 cells, cells were incubated with culture media containing 0.5 mM IBMX, 1 µM dexamethasone, and 5 µg/mL insulin under humidity and 5% CO_2_ at 37 °C for 2 days. The differentiation media were changed to culture media containing 5 µg/mL insulin and further incubated for 2 days. Then, the cells were maintained in complete media, which was changed every 2 days until adipocytes containing lipid droplets were seen.

### Determination of cell viability

An MTT assay was used to assess cell viability of L6 myotubes and 3T3-L1 adipocytes. First, L6 myoblasts and 3T3-L1 pre-adipocytes were differentiated into L6 myotubes and 3T3-L1 adipocytes as described above. Then, the differentiated cells were treated with ECR at various concentrations for 48 h. Subsequently, the cells were incubated with 100 µL of MTT solution (0.45 mg/mL) for 3 h at 37 °C in a humidified atmosphere of air and 5% CO_2_. The MTT solution was carefully removed, and 100 µL of DMSO was added. Untreated cells were the negative control. Absorbance (A) was measured by a microplate reader (Anthros, Durham, NC, USA) at 570 nm. Cell viability was determined as the following equation: % Cell viability = (A_sample_/A_control_) × 100, where A_sample_ and A_control_ were the absorbances in the presence and absence of sample, respectively.

### Hoechst33342/PI staining

A nuclear staining assay was performed to characterize cell death. After treating the cells with ECR for 48 h, the medium was replaced with 100 µL of PBS containing Hoechst33342 (2 µg/mL) and PI (1 µg/mL) and incubated in a dark place at 37 °C for 30 min. The fluorescent-stained cells were examined under a fluorescence microscope (Olympus IX51 with DP70, Tokyo, Japan).

### Glucose uptake assay in L6 myotubes and 3T3-L1 adipocytes

To determine the glucose uptake activity, a GO assay kit was used to determine the glucose levels in the media. The differentiated cells were treated either with ECR, insulin, or metformin and then incubated under humidity and 5% CO_2_ at 37 °C for 48 h. Next, a GO assay kit was used to analyze the media according to the manufacturer’s instruction. Briefly, the collected media were diluted with deionized water at a ratio of 1:150. Then, the diluted solution (25 μL) was mixed with 50 μL of glucose level-detecting reagent and incubated for 30 min. Next, 50 μL of 6M sulfuric acids was added to produce a stable, pink-colored product proportioned to the concentration of glucose. Absorbance was measured by a microplate reader at 540 nm and calculated for glucose that remained in the culture media.

#### Oil Red O staining

The content of lipid droplets in mature adipocytes was measured by ORO staining. After differentiation, as mentioned above, the cells were exposed to ECR for 48 h. Then, the cells were washed with PBS and fixed with 10% w/v formalin for 15 min at room temperature (RT). After fixing, the cells were incubated with ORO staining solution for 1 h and then washed with distilled water and 60%v/v isopropanol. Stained cells were photographed under a light microscope. The ORO staining cellular lipid was extracted into absolute isopropanol, and the absorbance at 510 nm was measured by a microplate reader. The level of extracted ORO was normalized to the total protein content measured with a BCA protein assay kit, following the manufacturer’s instructions. The data were reported as %ORO relative to the untreated control group^5^.

### Western blotting analyses

After exposure to ECR for 48 h, the cells were washed with PBS. The cell membranes were disrupted by adding RIPA buffer supplemented with a protease inhibitor cocktail and then incubated on ice for 45 min. The cell lysates were centrifuged at 12,000 rpm at 4 °C for 15 min, and a BCA assay kit was used to measure the total protein content. Protein samples (45 μg) were run on 10% sodium dodecyl sulfate–polyacrylamide gel electrophoresis (SDS-PAGE). The separated proteins were transferred onto nitrocellulose membranes and blocked with 5% BSA in TBST buffer for 1 h. The membranes were immunoblotted with specific primary antibodies at 4 °C overnight. After the membranes were washed with TBST for 5 min three times, the membranes were incubated with horseradish peroxidase-conjugated secondary antibody at RT for 2 min. Next, the membranes were washed with TBST for 5 min three times, and the reactive protein signals exposed with chemiluminescent substrates were detected^5^. The ImageJ program was used to determine the band intensity of the target protein and calculated relative to β-actin. The uncropped gels were displayed in Figs. S11–S13.

### Immunofluorescent labeling assay for translocation of glucose transporter

To study the GLUT translocation in L6 myotubes and 3T3-L1 adipocytes, we treated the cells either with ECR (100 μg/mL), insulin (100 nM), or metformin (1 mM) for 48 h at 37 °C. Subsequently, the cells were washed with ice-cold PBS three times and fixed with 4% formalin for 10 min at RT. Next, the cells were rinsed with PBS three times and incubated with 0.1% Triton X-100 for 10 min at RT. The cells were blocked with 3% BSA and incubated overnight at 4 °C with the primary antibodies (GLUT1, D3J3A Rabbit mAb; GLUT4, 1F8 Mouse mAb). After washing with PBS three times, the cells were incubated with their respective fluorescent secondary antibodies, Alexa Fluor™ 594 goat anti-rabbit IgG (H + L) for GLUT1 and Alexa Fluor™ 488 goat anti-mouse IgG (H + L) for GLUT4 for 2 h at RT in the dark. After incubation, the cells were washed with PBS three times and incubated with Hoechst33342 for 30 min at RT in the dark. After three additional PBS washes, a confocal microscope (Zeiss LSM 900 with Airyscan 2, Jena, Germany) was used to image the cells mounted in a medium^39^.

### Statistical analysis

The results were expressed as mean ± standard error of means (SEM) of the three independent experiments. Analysis of variance (ANOVA) was conducted using the GraphPad Prism Version 7.0 (GraphPad Software Inc., San Diego, CA, USA). A significant difference was addressed when the *p*-value was below 0.05.

## Conclusion

**Figure 7:**
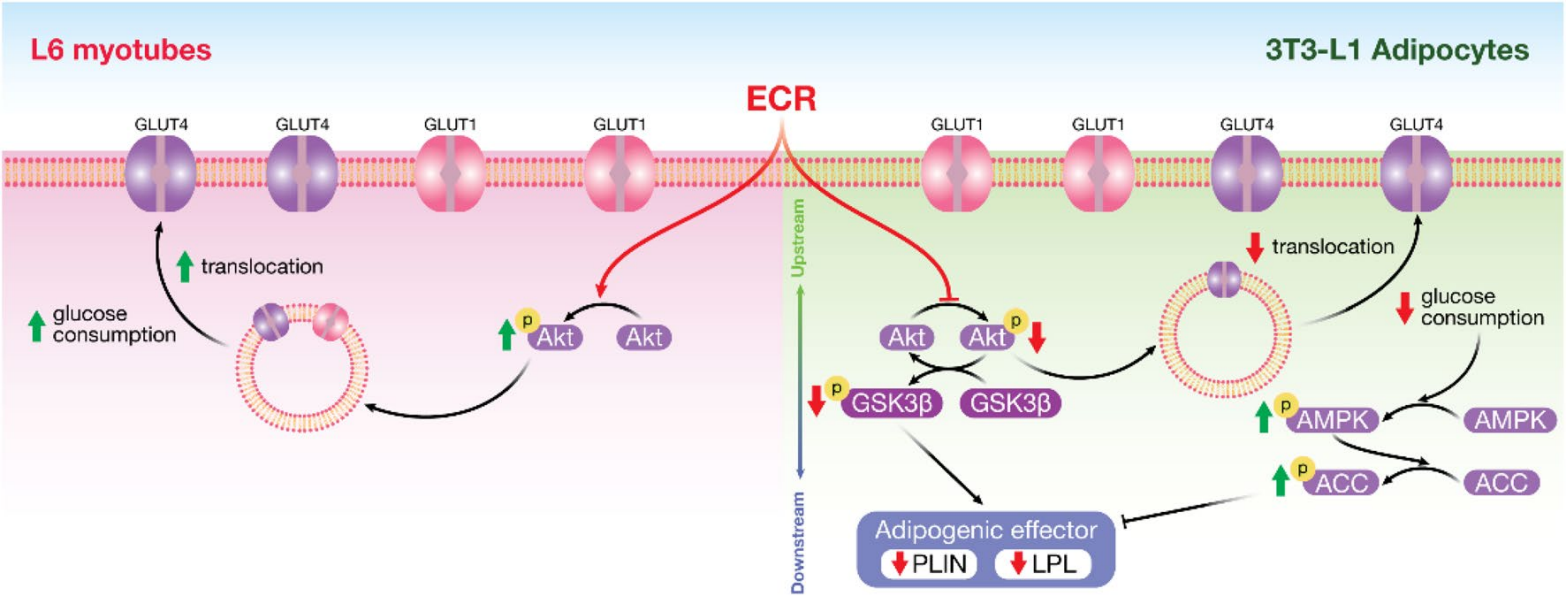
Elucidated mechanisms of ECR on enhancing glucose uptake in L6 myotubes and diminishing glucose uptake and adipocyte maturation in 3T3-L1 adipocytes.

The anti-radical ECR extract containing 1.61%w/w piceatannol used in this study dose-dependently stimulated significant expression and translocation of GLUT1 and 4 in ECR-incubated L6 myotubes. Additionally, GLUT4 expression and translocation decreased in 3T3-L1 adipocytes, especially at 100 μg/mL ECR. In the adipogenesis study, ECR suppressed lipid accumulation by activating the AMPK–ACC pathway and deactivating the Akt/GSK3β pathway. Moreover, ECR inhibited PLIN1 and LPL expression, indicating de novo lipogenesis pathway interruption (Fig. 7). The overall effect of ECR stimulates skeletal-muscle glucose consumption and inhibits lipid accumulation, which promotes good health.

### Supplementary Information

Supplementary Information.

## Data Availability

Experimental data are available. Please contact C. Chansriniyom (Chaisak.ch@chula.ac.th).
