# Supplementary material for: Diverse effects of a Cyperus rotundus extract on glucose uptake in myotubes and adipocytes and its suppression on adipocyte maturation
Source: Sci Rep. 2024 Apr 19;14:9018. doi: 10.1038/s41598-024-59357-0 (PMC11031566; doi:10.1038/s41598-024-59357-0)
Supplement: Supplementary file 1 — Supplementary Information. [file 41598_2024_59357_MOESM1_ESM.pdf]

Supplementary Information

Diverse effects of a *Cyperus rotundus* extract on glucose uptake in myotubes and adipocytes and its suppression on adipocyte maturation

Vipawee Pichetkun[1,2], Hnin Ei Ei Khine[3,4], Suchada Srifa[3,4], Sasiwimon Nukulkit[2], Nitra Nuengchamnong[5], Supakarn Hansapaiboon[6], Rattaporn Saenmuangchin[7], Chatchai Chaotham[3,4,*], Chaisak Chansriniyom[2,8,*]

[1]Pharmaceutical Sciences and Technology Program, Faculty of Pharmaceutical Sciences, Chulalongkorn University, Bangkok 10330, Thailand
[2]Natural Products and Nanoparticles Research Unit, Faculty of Pharmaceutical Sciences, Chulalongkorn University, Bangkok 10330, Thailand
[3]Department of Biochemistry and Microbiology, Faculty of Pharmaceutical Sciences, Chulalongkorn University, Bangkok 10330, Thailand
[4]Preclinical Toxicity and Efficacy Assessment of Medicines and Chemicals Research Unit, Faculty of Pharmaceutical Sciences, Chulalongkorn University, Bangkok 10330, Thailand [5]Science Laboratory Center, Faculty of Science, Naresuan University, Phitsanulok 65000, Thailand
[6]Pharmaceutical Research Instrument Center of the Faculty of Pharmaceutical Sciences, Chulalongkorn University, Bangkok 10330, Thailand
[7]National Nanotechnology Center, National Science and Technology Development Agency, 111 Phahonyothin Rd., Klongluang, Pathumthani, 12120, Thailand
[8]Department of Pharmacognosy and Pharmaceutical Botany, Faculty of Pharmaceutical Sciences, Chulalongkorn University, Bangkok 10330, Thailand
[*]Corresponding authors: Chaisak Chansriniyom, Tel.: +66-2218-8347, E-mail address: chaisak.ch@chula.ac.th and Chatchai Chaotham, Tel.: +66-2218-8372, E-mail address: chatchai.c@chula.ac.th

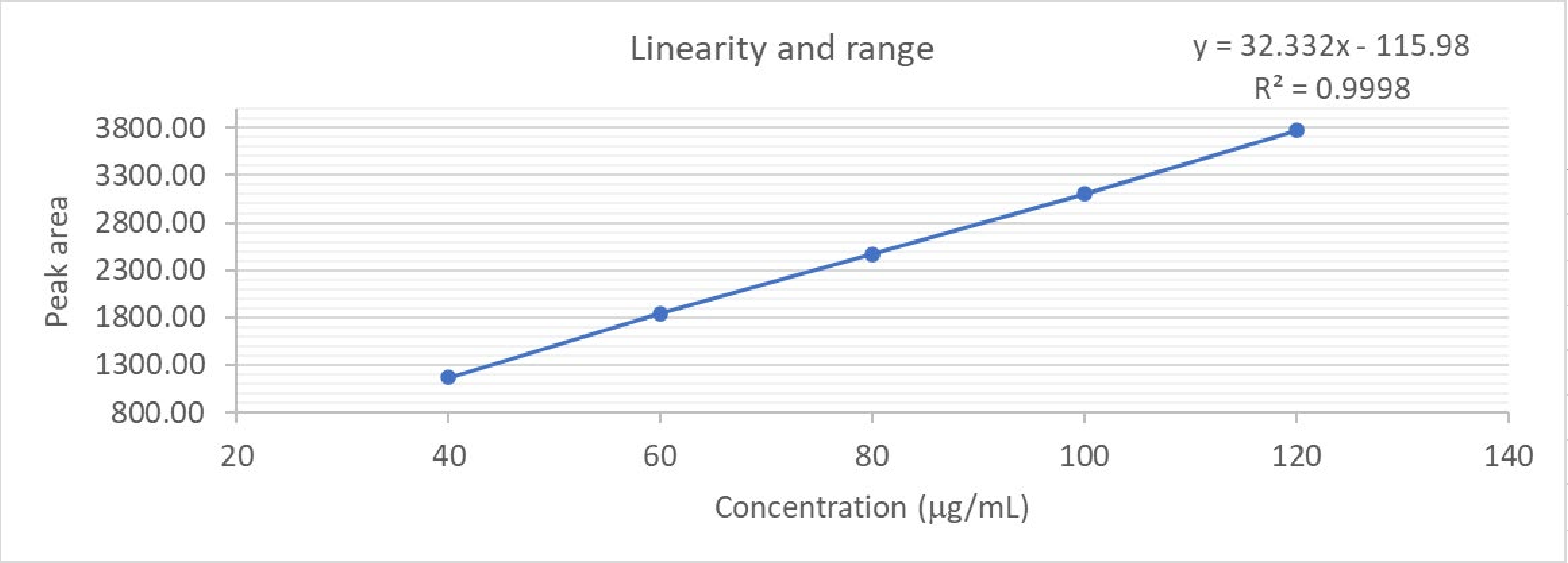

SUMMARY OUTPUT

Regression Statistics	
Multiple R	0.999883578
R Square	0.99976717
Adjusted R Square	0.99968956
Standard Error	18.01649958
Observations	5

ANOVA

	df	*SS*	*MS*	*F*	*Significance F*
Regression	1	4181408.877	4181408.9	12881.956	1.50791E-06
Residual	3	973.7827709	324.59426		
Total	4	4182382.66			

	Coefficients	*Standard Error*	*t Stat*	*P-value*	*Lower 95%*	*Upper 95%*	*Lower 95.0%*	*Upper 95.0%*
Intercept	-115.9767522	24.17167066	-4.7980445	0.0172272	-192.9017962	-39.051708	-192.901796	-39.0517082
Conc. (ug/mL)	32.33190714	0.284865871	113.4987	1.508E-06	31.4253368	33.2384775	31.4253368	33.23847748

Figure S1.1. Linearity and range of piceatannol in a range of concentrations from 4 to 12 µg/mL and regression analysis.

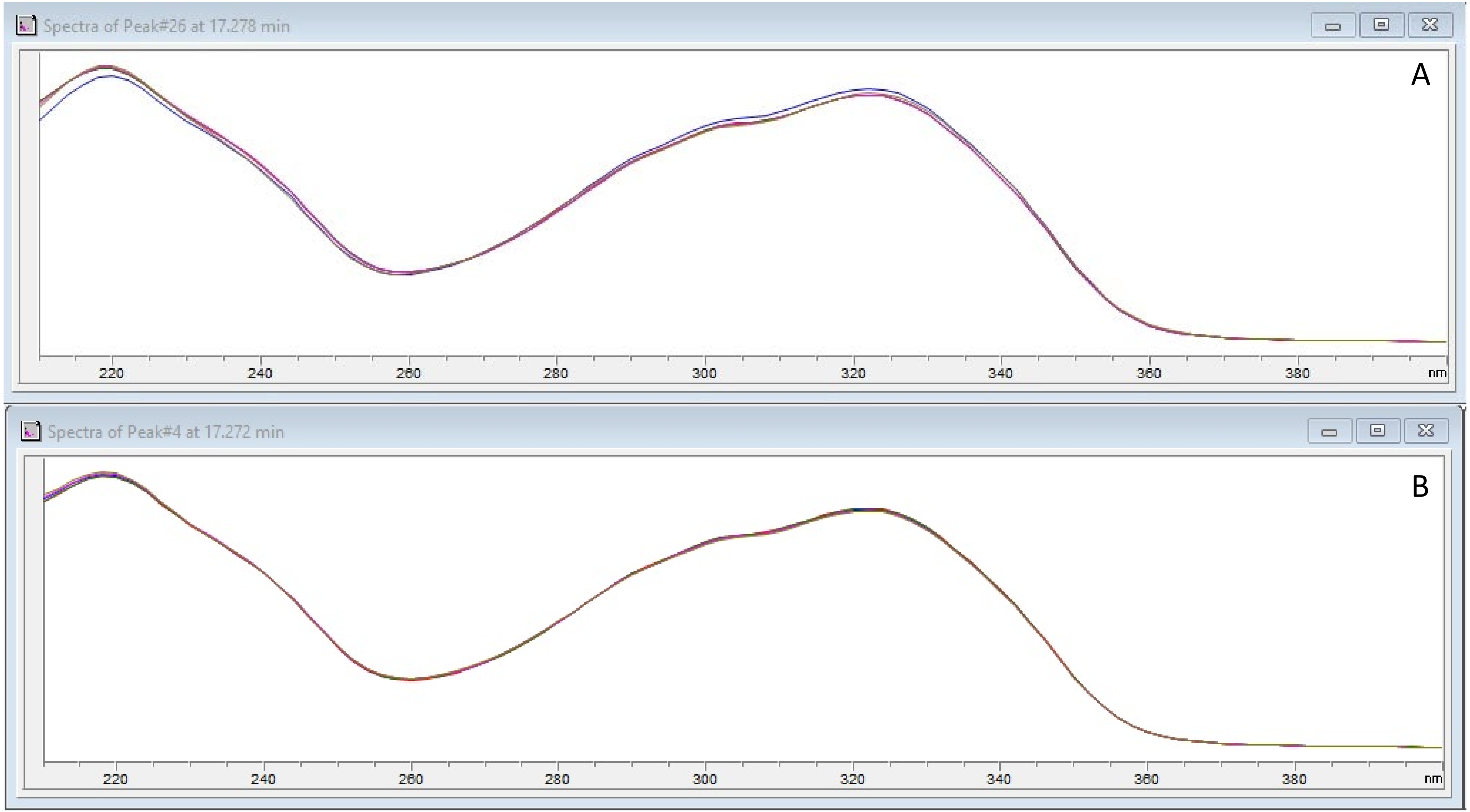

Figure S1.2. UV absorption spectra of the peak at t_r = 17.278 min of ECR (A) and the peak at t_r = 17.272 min of piceatannol (B), displaying the specificity for analytical method validation.

Materials and methods: additional details

Characterization of chemical constituents in ECR: Liquid chromatography-tandem mass spectrometry (LC–MS/MS) analysis

The LC–MS/MS analysis was carried out using an Agilent 6540 Q-TOF-MS spectrometer (Agilent Technologies, Singapore) equipped with an Agilent 1260 Infinity Series High-performance liquid chromatography system (Agilent Technologies, Waldbronn, Germany). Five µL of ECR in MeOH (40 mg/mL) was injected into a Luna C18(2) column (150 × 4.6 mm, 5 µm, Phenomenex, USA). The mobile phase was 0.1% v/v formic acid in type 1 water (solvent A) and 0.1% v/v formic acid in acetonitrile (solvent B). The gradient elution started at 5% to 95% solvent B within 30 min and held on for 10 min. The flow rate was 0.5 mL/min, and the column temperature was 35°C. The electrospray ionization (ESI) was performed in both positive and negative modes with parameters as follows: N_2 (drying gas) flow rate, 10.0 L/min; temperature, 350°C; nebulizer pressure, 30 psig; capillary voltage, 3500 V; skimmer voltage, 65 V; octapole RFV, 750 V; fragment voltage, 250 V for negative and 100 V for positive mode. The mass ranged from 100 to 1000 *m/z,* and scan rate was 4 spectra/s. The collision energies of 10, 20, and 40 V were applied with high-purity nitrogen gas. MassHunter Qualitative Analysis Software B 06.0 (Agilent Technologies, CA, USA) was used for data analysis. The databases such as Human Metabolome, Lipid maps, and METLIN were used to identify the compounds.

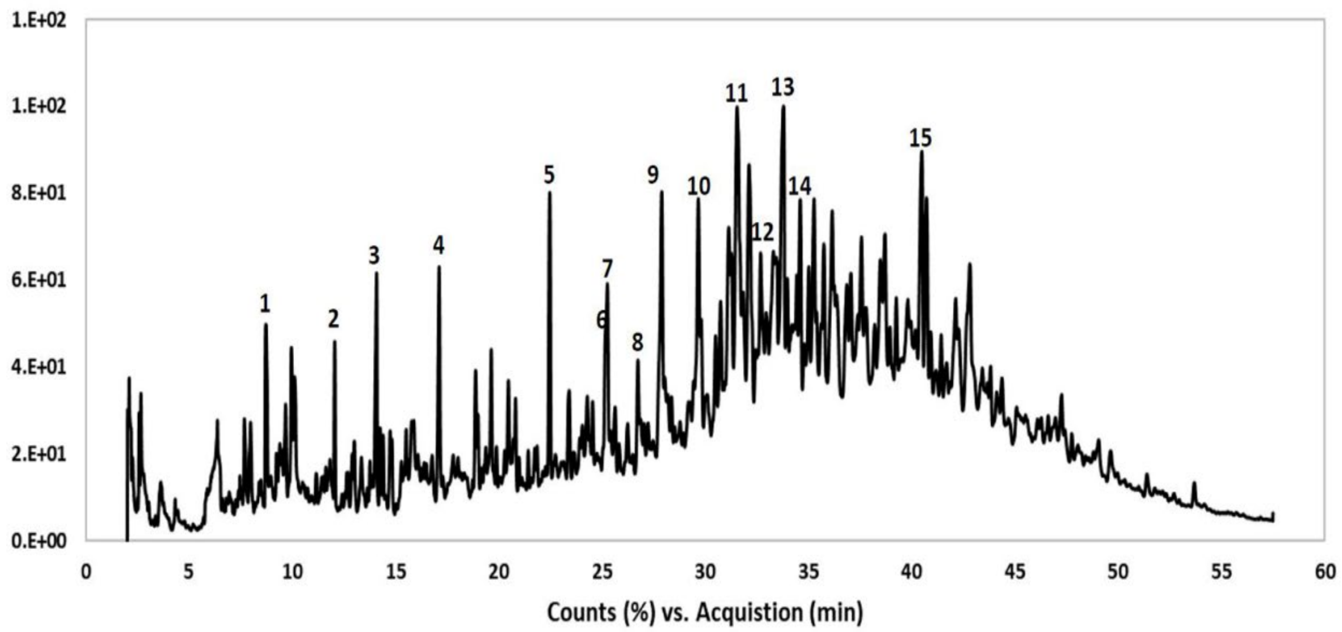

Figure S2. GC–MS chromatogram of ECR (10 mg/mL) operated in positive ionization mode. The peaks were labeled corresponding to the number in Tables S1.

Table S1 Tentative identification of phytochemicals in ECR identified by GC–MS/MS

No.	t_r (min)[a]	Mass (DB)[b]	Base peak	MS/MS fragmentation	Identification	Formula	Score
1	8.729	144.0	144.0	144.0, 101.0, 73.0, 43.2	3,5-dihydroxy-6-methyl-2,3-dihydropyran-4-one	$C_6H_8O_4$	87.43
2	12.063	150.0	135.0	135.0, 107.0, 77.0, 55.0	2-*tert*-butylphenol	$C_{10}H_{14}O$	87.89
3	14.09	170.0	59.1	137.0, 109.0, 79.1, 59.1	5-(2-hydroxypropan-2-yl)-2-methylcyclohex-2-en-1-ol	$C_{10}H_{18}O_2$	88.97
4	17.12	220.0	79.1	152.0, 109.0, 79.1, 59.1	cis-*Z*-α-Bisabolene epoxide	$C_{15}H_{24}O$	81.08
5	22.478	220.0	109.1	138.0, 109.1, 96.1, 67.1	Caryophyllene epoxide	$C_{15}H_{24}O$	89.21
6	25.184	198.0	183.0	183.0, 168.0, 91.0, 43.1	Cadalene	$C_{15}H_{18}$	83.26
7	25.266	190.0	175.0	175.0, 119.0, 91.0, 43.1	Dehydroionone	$C_{13}H_{18}O$	82.8
8	26.746	236.0	91.0	133.0, 91.0, 43.1	6-(3-hydroxyprop-1-en-2-yl)-4,8a-dimethyl-2,4a,5,6,7,8-hexahydro-1H-naphthalen-2-ol	$C_{15}H_{24}O_2$	89.78
9	27.901	236.0	43.1	135.0, 93.1, 43.1	Corymbolone	$C_{15}H_{24}O_2$	84.98
10	29.669	252.0	43.1	164.0, 133.0, 91.0, 43.1	6'-methyl-3'-propan-2-ylspiro[oxirane-2,7'-tricyclo[4.4.0.0^{2,8}]decane]-9',10'-diol	$C_{15}H_{24}O_3$	88.29
11	31.548	238.0	109.1	147.1, 109.1, 107.1, 55.2	7-(1-hydroxypropan-2-yl)-1,4a-dimethyl-3,4,5,6,7,8-hexahydro-2H-naphthalen-2-ol	$C_{15}H_{26}O_2$	87.48
12	32.685	252.0	43.1	234.0, 159.0, 91.0, 43.1	Deoxyuvidin B	$C_{15}H_{24}O_3$	88.35
13	33.797	238.0	107.1	220.1, 159.1, 109.1, 107.1, 55.2	7-(1-hydroxypropan-2-yl)-1,4a-dimethyl-3,4,5,6,7,8-hexahydro-2H-naphthalen-2-ol	$C_{15}H_{26}O_2$	88.31
14	34.61	238.0	164.0	220.1, 164.0, 135.0, 107.0, 43.1	Clovane diol	$C_{15}H_{26}O_2$	91.25
15	40.499	252.0	222.1	222.1, 107.1, 95.1, 81.1, 43.1	Lactaropallidin	$C_{15}H_{24}O_3$	86.73

[a]retention time; [b]database

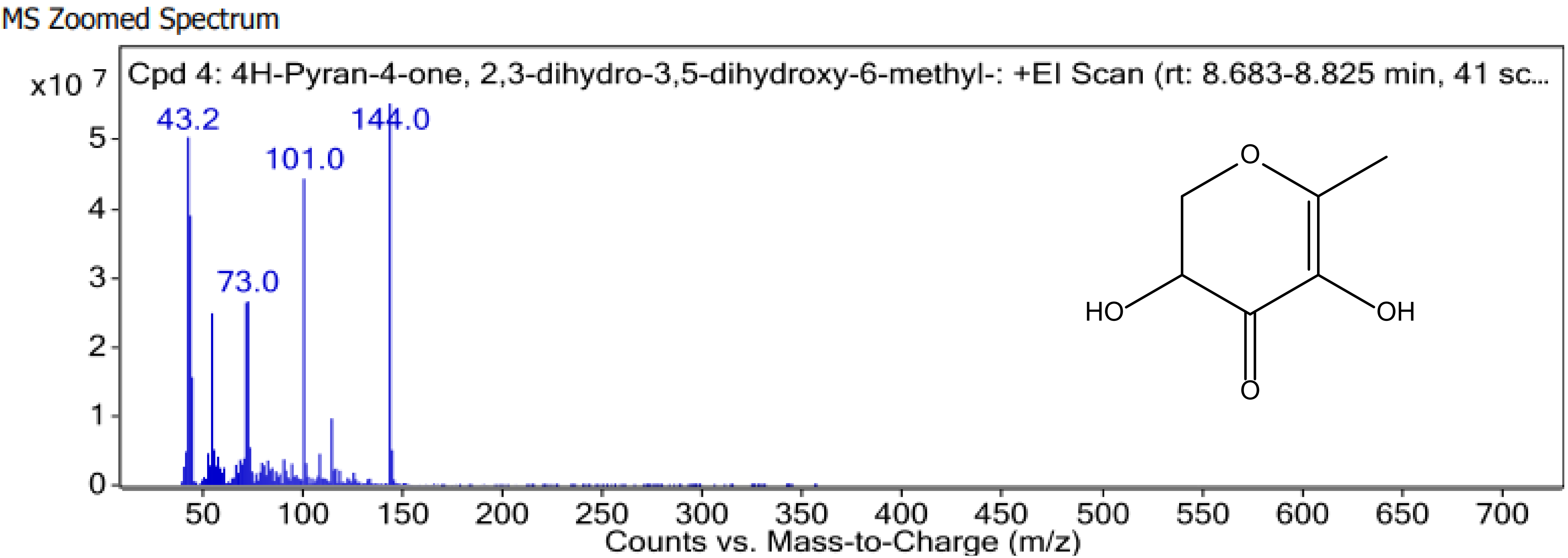

Figure S2.1. GC-MS/MS fragmentation of compound No 1 at m/z 144.0.

MS Zoomed Spectrum

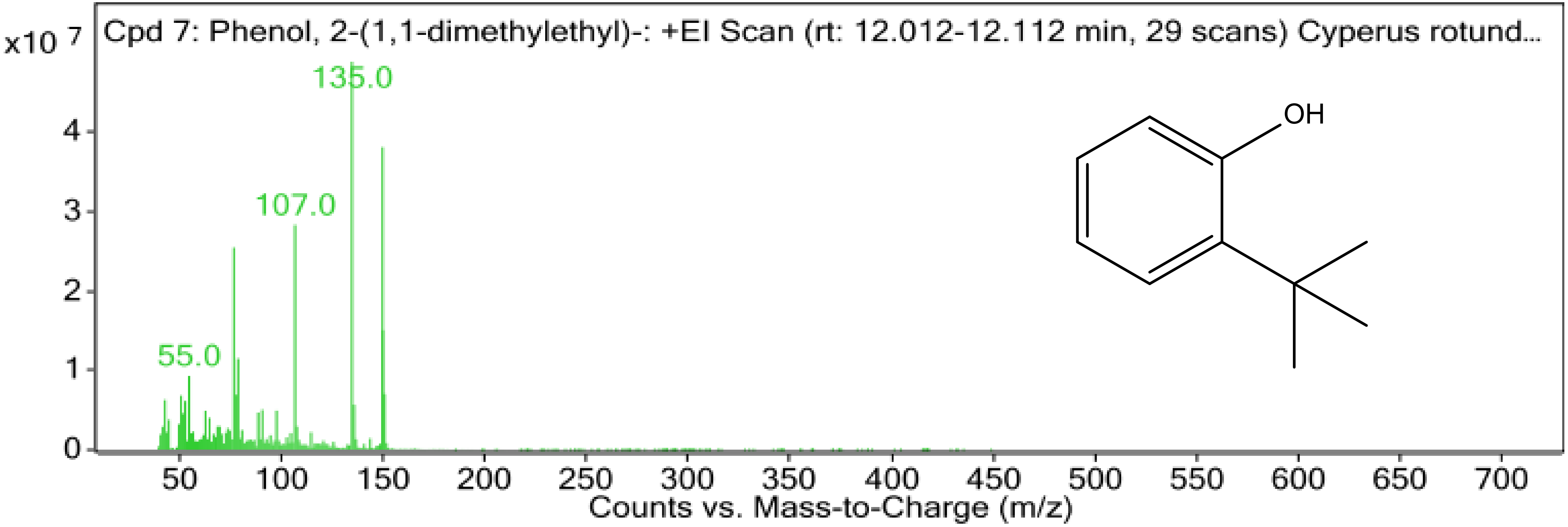

Figure S2.2. GC-MS/MS fragmentation of compound No 2 at m/z 150.0.

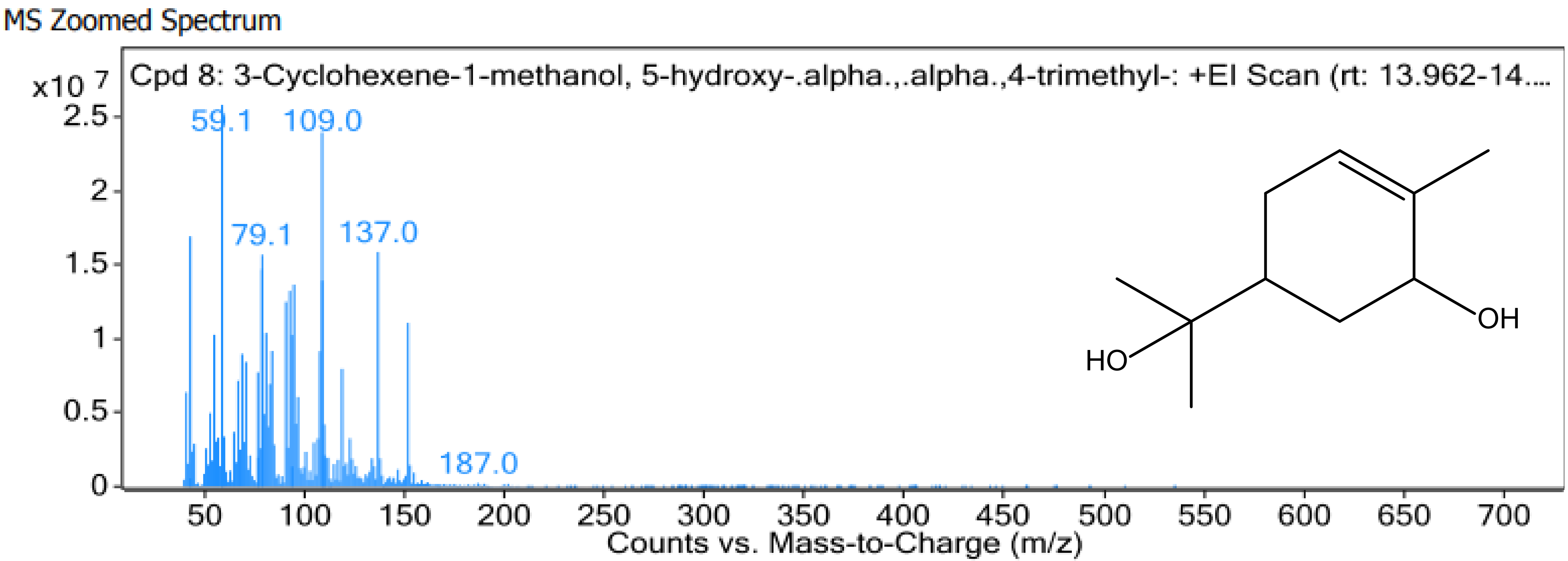

Figure S2.3. GC-MS/MS fragmentation of compound No 3 at m/z 170.0.

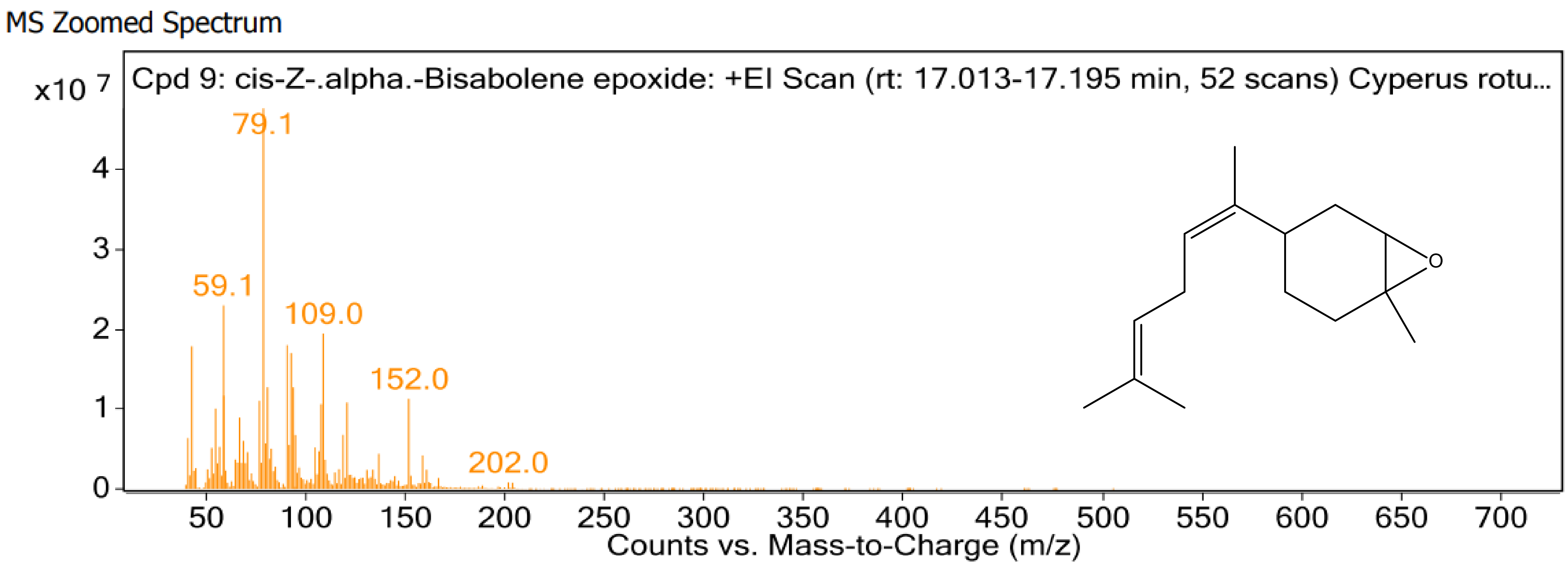

Figure S2.4. GC-MS/MS fragmentation of compound No 4 at m/z 220.0.

MS Zoomed Spectrum

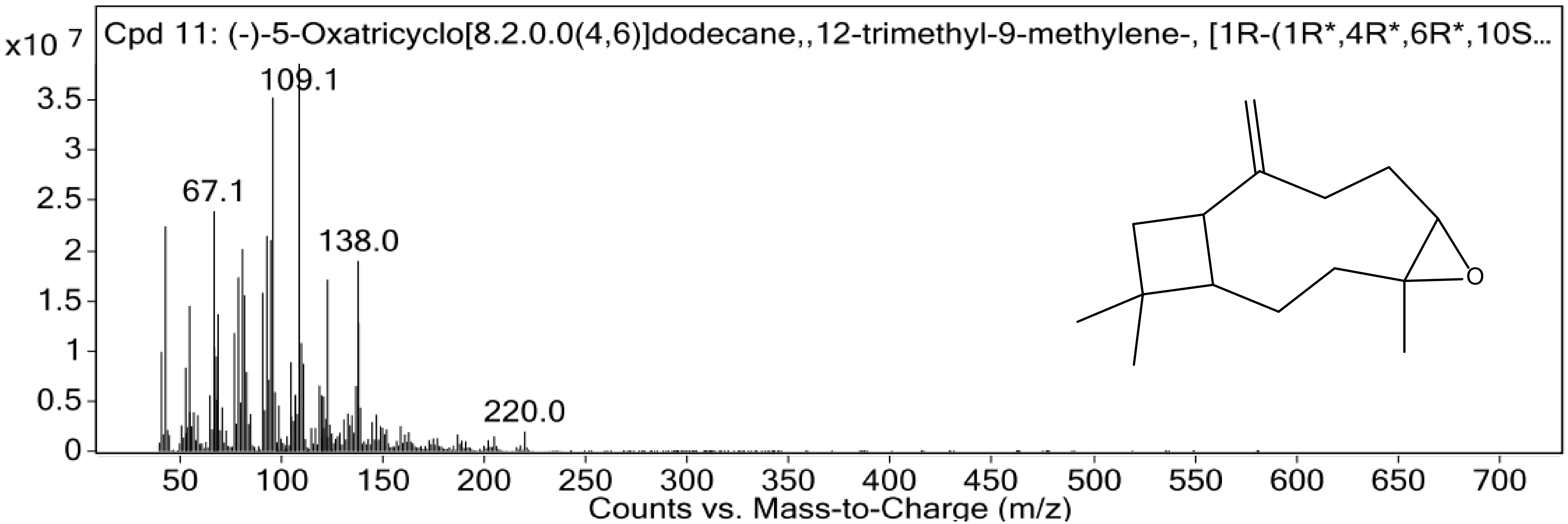

Figure S2.5. GC-MS/MS fragmentation of compound No 5 at m/z 220.0.

MS Zoomed Spectrum

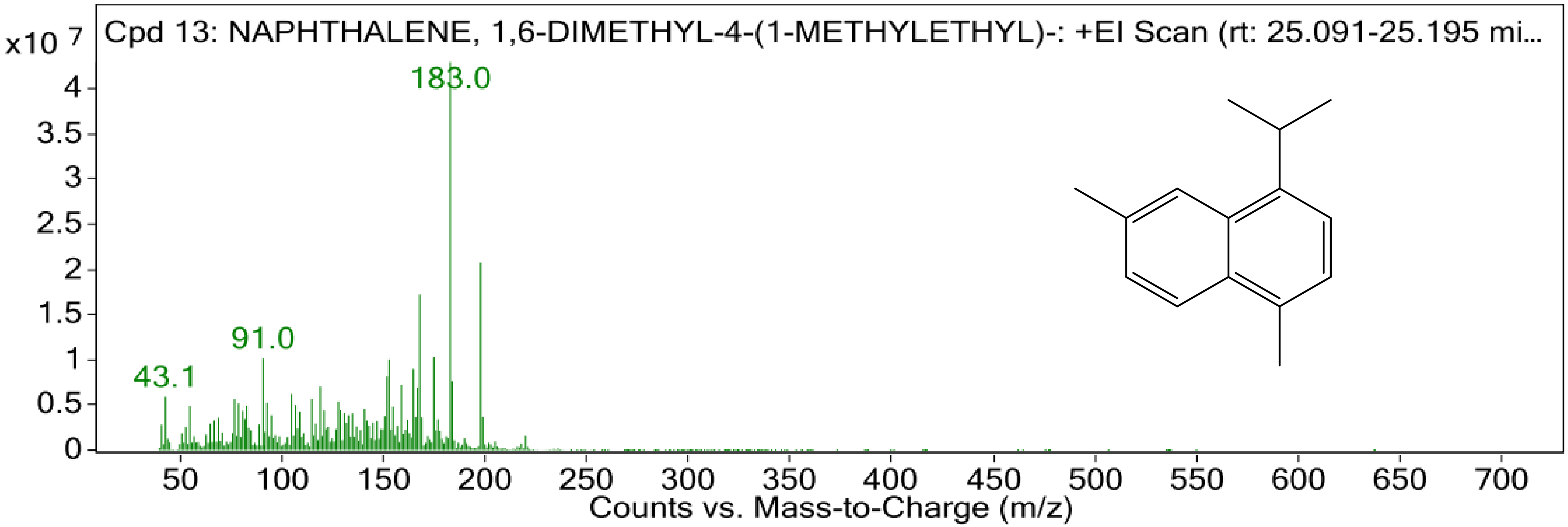

Figure S2.6. GC-MS/MS fragmentation of compound No 6 at m/z 198.0.

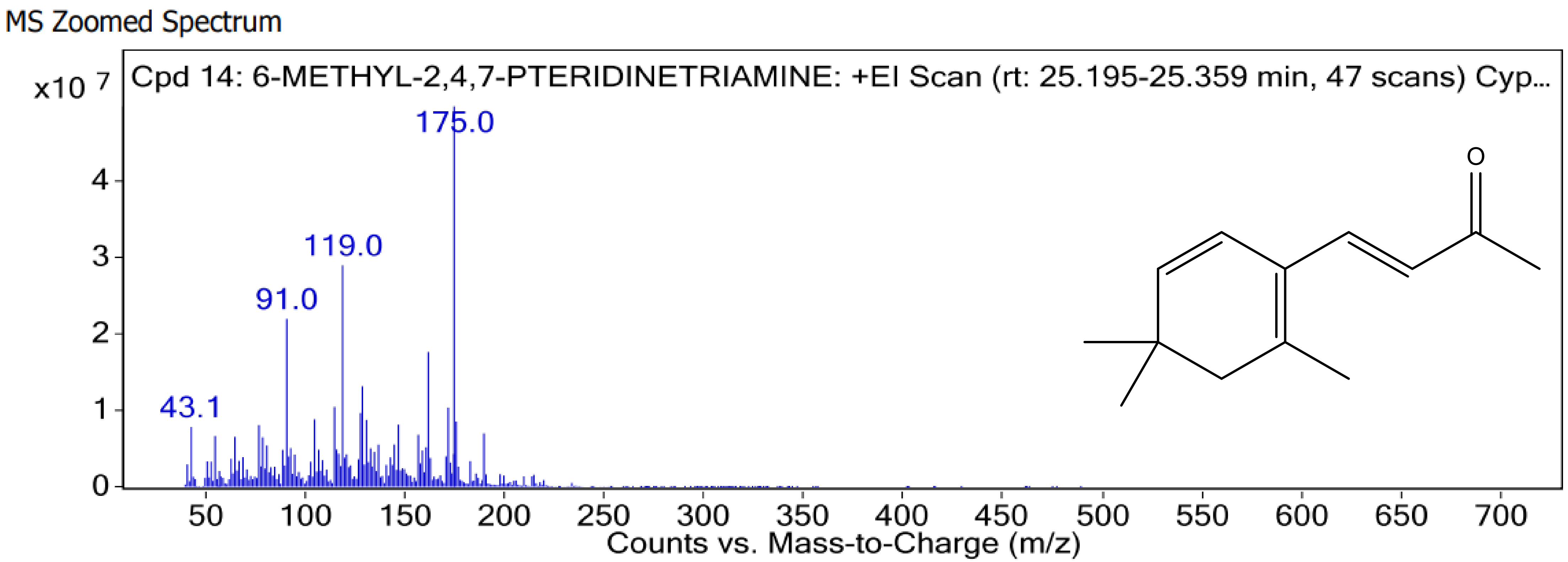

Figure S2.7. GC-MS/MS fragmentation of compound No 7 at m/z 190.0.

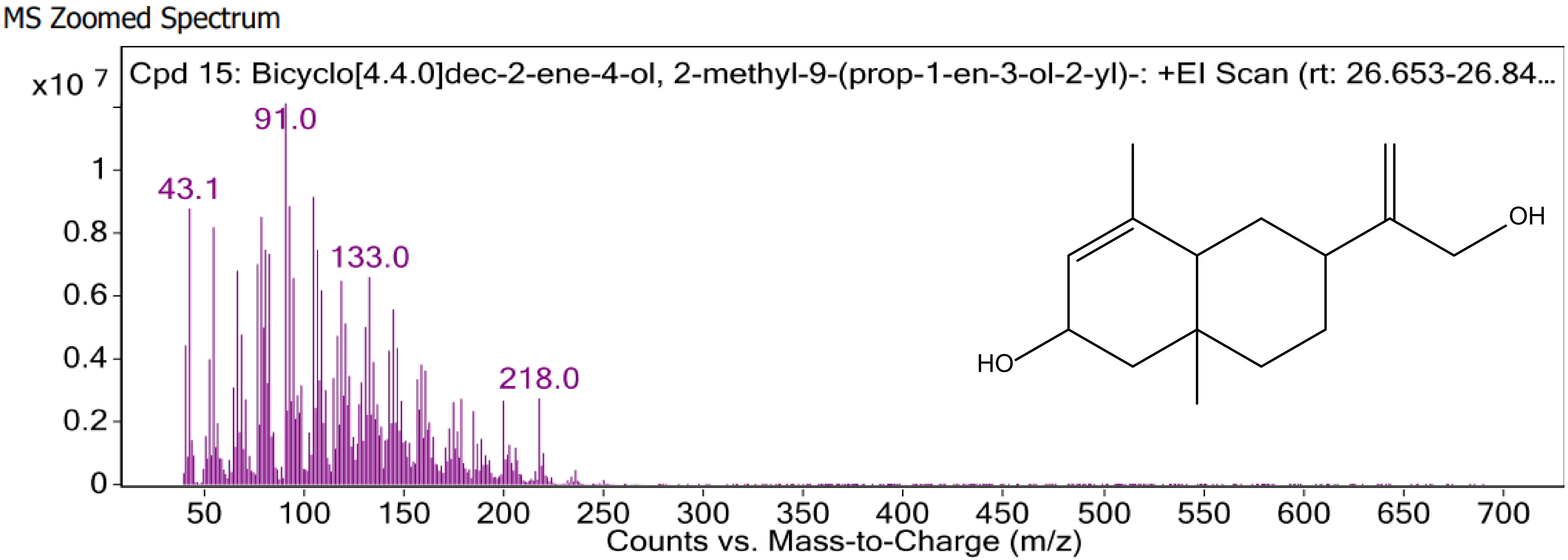

Figure S2.8. GC-MS/MS fragmentation of compound No 8 at m/z 236.0.

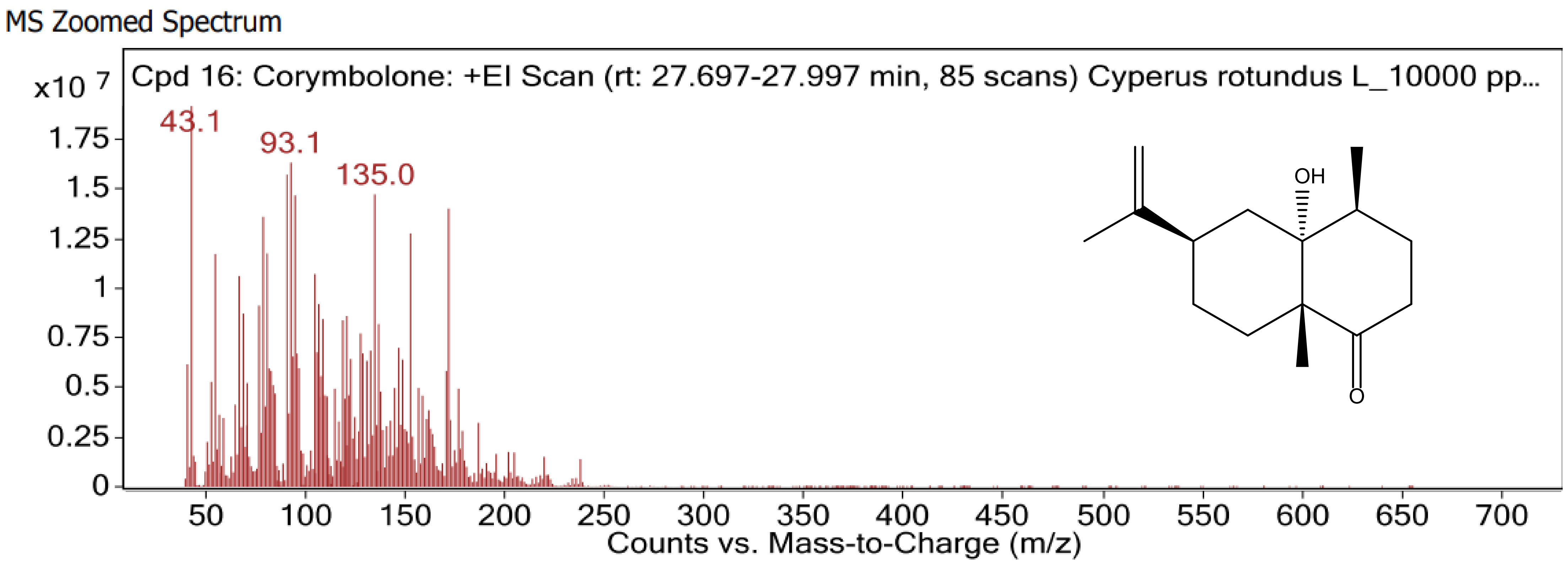

Figure S2.9. GC-MS/MS fragmentation of compound No 9 at m/z 236.0.

MS Zoomed Spectrum

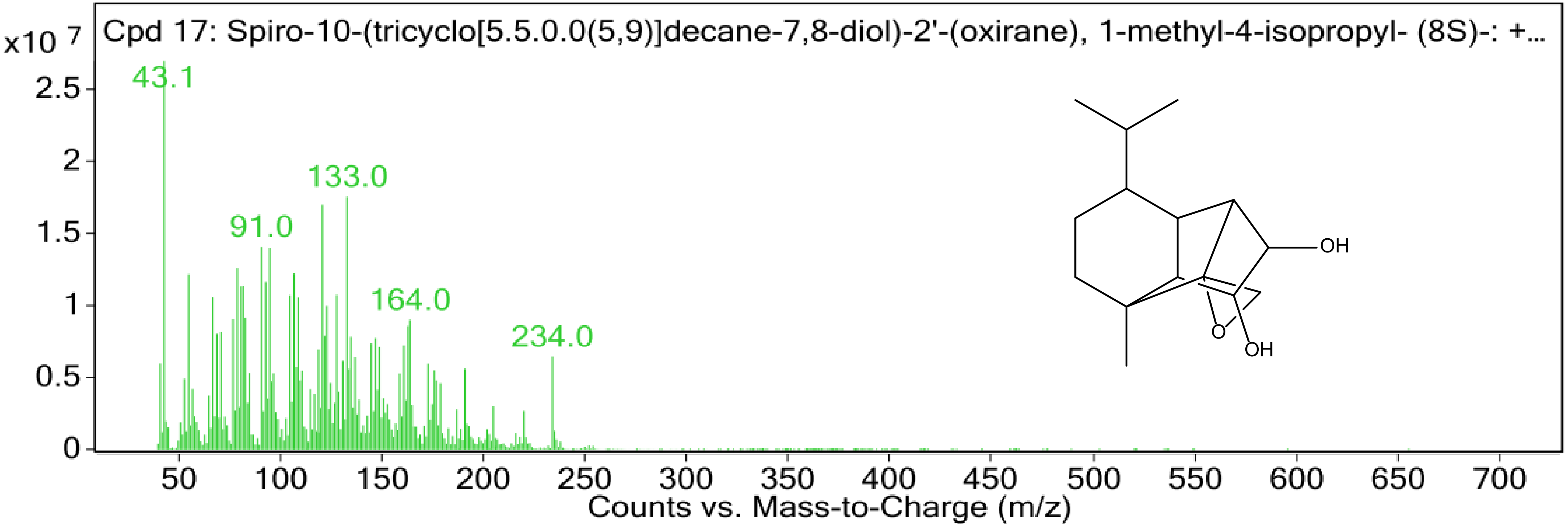

Figure S2.10. GC-MS/MS fragmentation of compound No 10 at m/z 252.0.

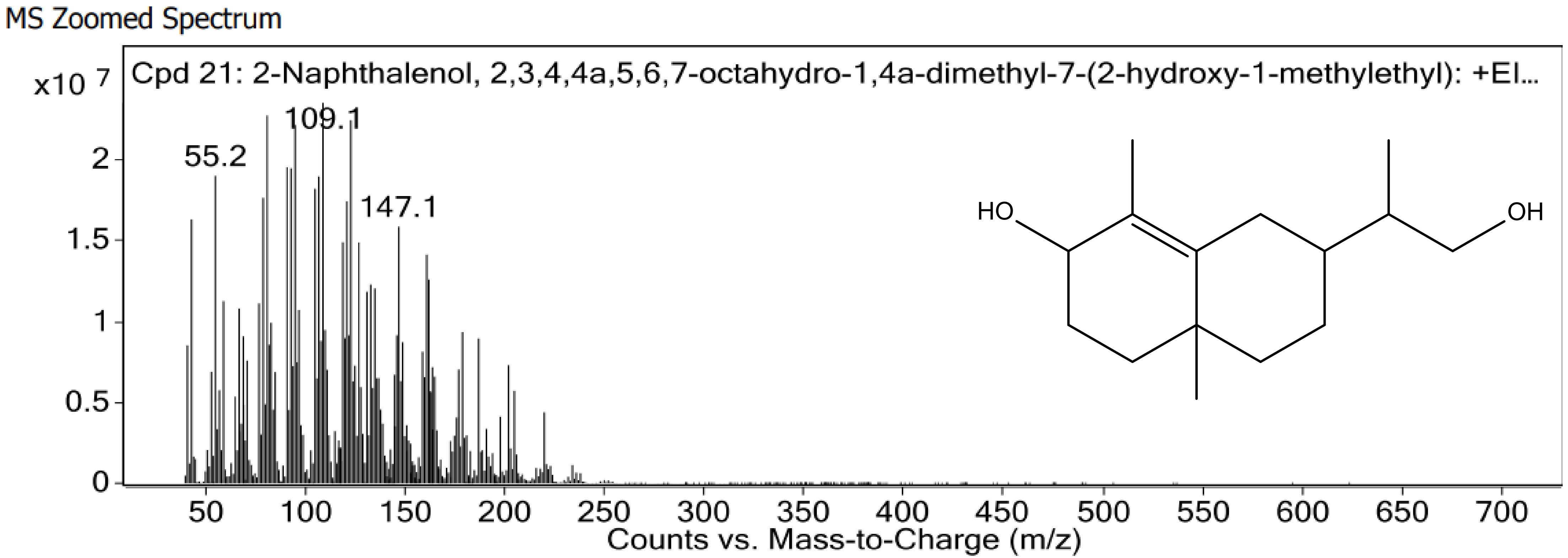

Figure S2.11. GC-MS/MS fragmentation of compound No 11 at m/z 238.0.

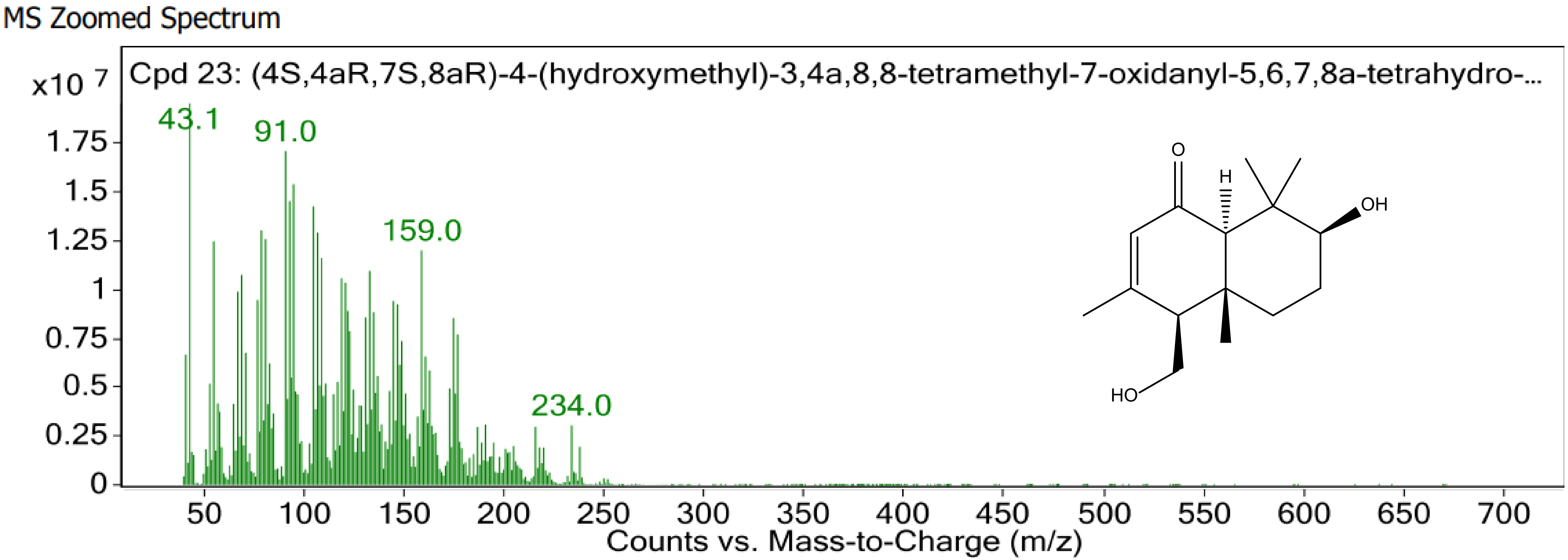

Figure S2.12. GC-MS/MS fragmentation of compound No 12 at m/z 252.0.

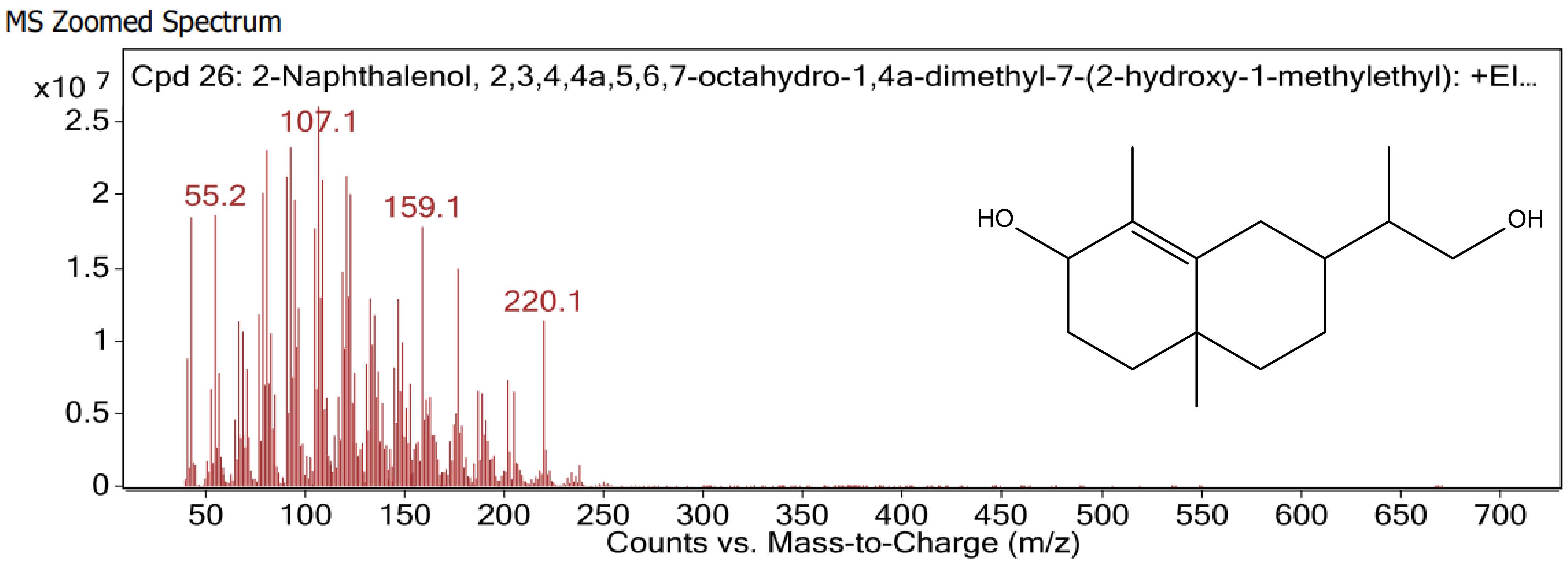

Figure S2.13. GC-MS/MS fragmentation of compound No 13 at m/z 238.0.

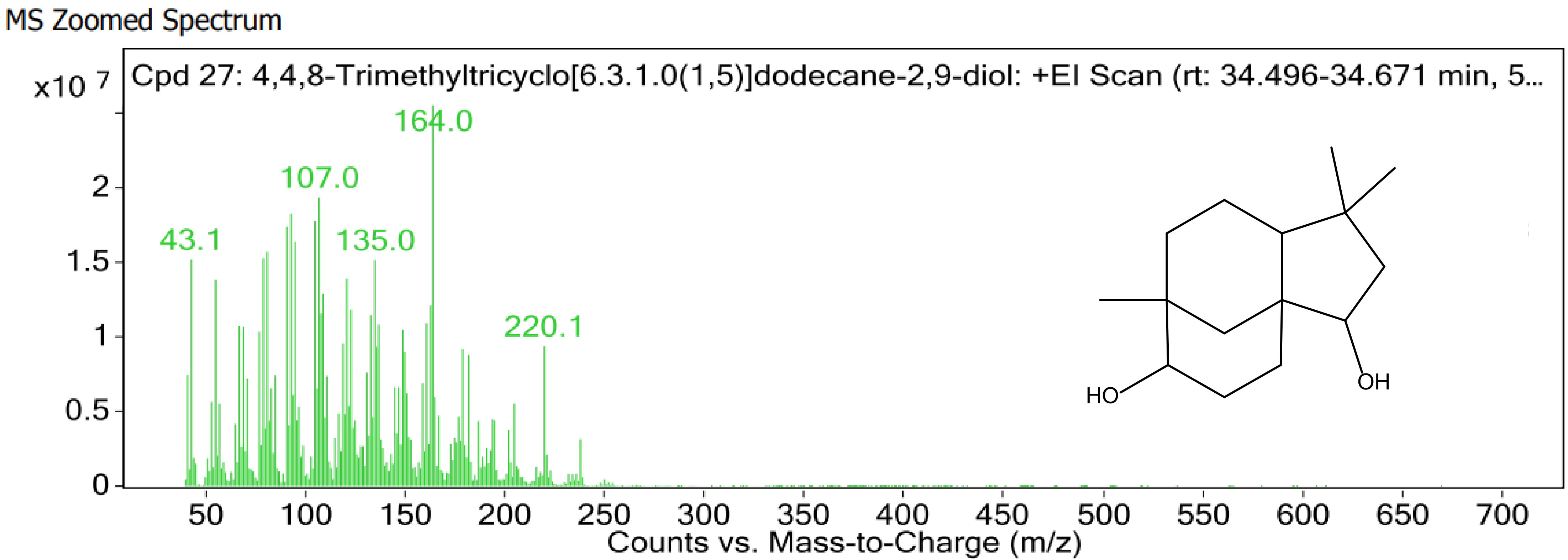

Figure S2.14. GC-MS/MS fragmentation of compound No 14 at m/z 238.0.

MS Zoomed Spectrum

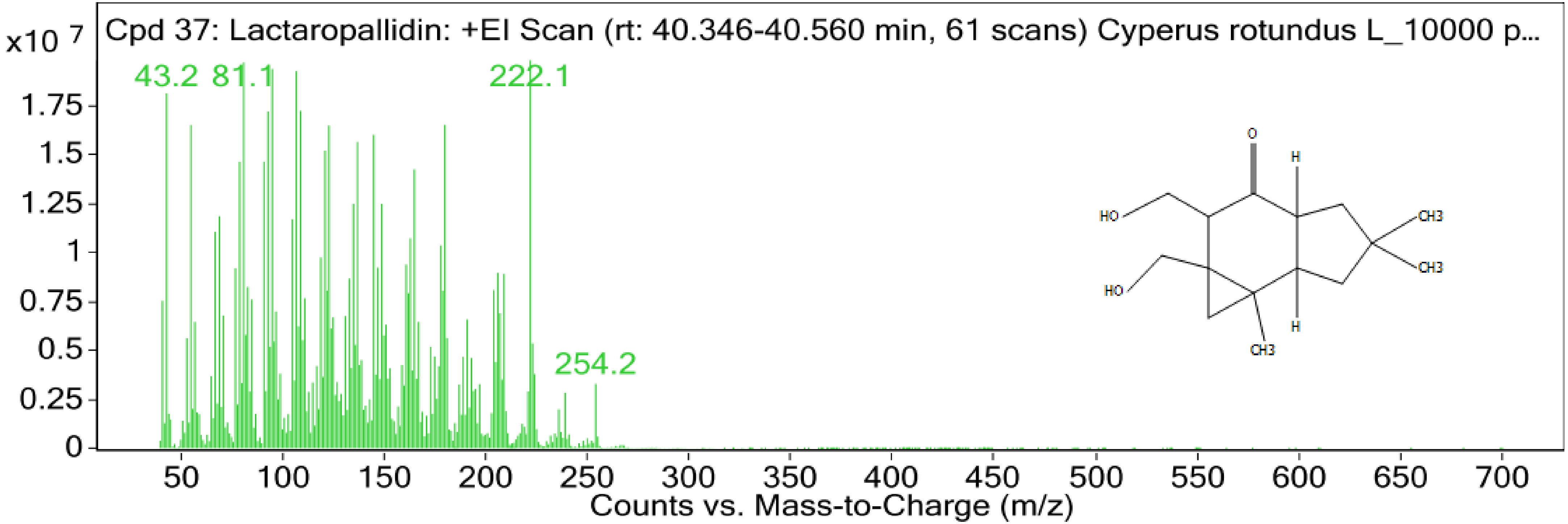

Figure S2.15. GC-MS/MS fragmentation of compound No 15 at m/z 252.0.

Materials and methods: additional details

Characterization of chemical constituents in ECR: Gas chromatography-tandem mass spectrometry (GC–MS/MS) analysis

The GC-MS/MS analysis was operated on GC–MS Triple Quadrupole (7890B-7010, Agilent Technologies, CA, USA). One microliter (μL) of ECR in MeOH (10 mg/mL) was injected into the HP-5ms Ultra Inert column (30 m × 250 μm × 0.25μm) in spitless mode with the constant helium flow of 1.2 mL/min. The injector temperature was kept at 250°C. The oven temperature was set at 70°C as the initial temperature and programmed to raise at a rate of 20°C/min until at 120°C and gradually changed at a rate of 2°C/min up to 220°C. The MS normal scanning was carried out with the following parameters: fragments ranging 40 – 700 *m/z*; threshold, 150; A/D samples, 4. The fragmentation pattern was analyzed and matched with MS spectral database of National Institute of Standards and Technology (NIST) library.

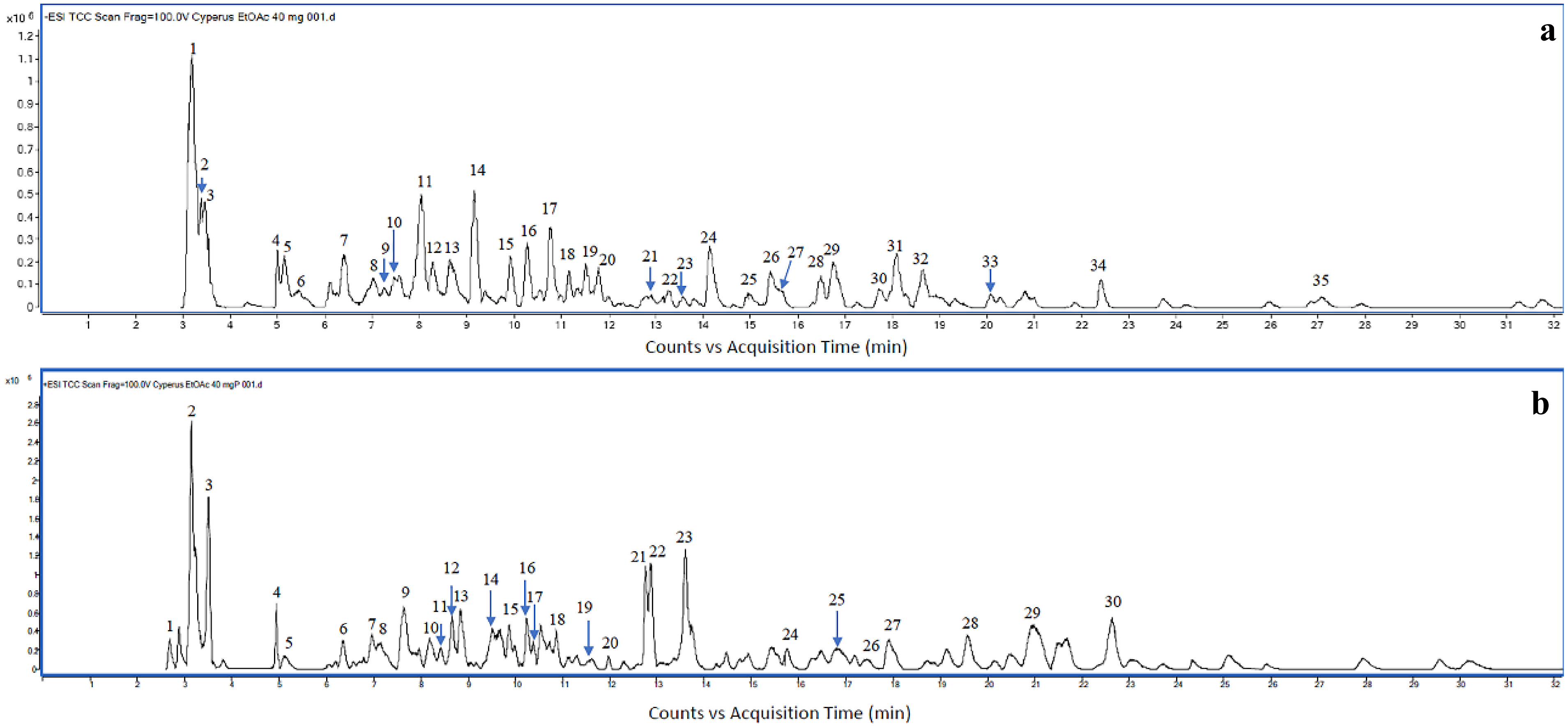

Figure S3. Total compound chromatogram of ECR (40 mg/mL) conducted by LC–ESI–MS/QTOF in negative (**a**) and positive (**b**) ionization modes. The peaks were labeled corresponding to the number in Tables S2 and S3.

Table S2. Tentative identification of phytochemicals in ECR identified by LC–MS/MS in negative mode

No.	t_r (min)[a]	m/z, ion: [M-H]⁻	MS/MS	Identification	Formula	Error (ppm)
1	3.133	179.057	89.0248, 59.0145	Hexose	$C_6H_{12}O_6$	-4.96
2	3.374	191.057	173.0453, 127.0427, 85.0298, 59.0144	Quinic acid	$C_7H_{12}O_6$	4.71
3	3.454	105.0197	83.6033, 59.0139	Glyceric acid	$C_3H_6O_4$	-3.5
4	4.98	145.0154	127.0033, 83.0142, 57.0348	Oxoglutaric acid	$C_5H_6O_5$	-7.95
5	5.145	128.0355	85.0303, 82.0316, 57.0347	4-Oxo-L-proline	$C_5H_7NO_3$	-1.43
6	5.468	147.0304	105.0191, 87.0191, 59.0143	Citramalic acid isomer	$C_5H_8O_5$	-3.42
7	6.406	147.0301	129.0195, 85.0298, 59.0141	2-Hydroxyglutaric acid	$C_5H_8O_5$	-1.38
8	7.066	169.0144	125.0244, 79.0188, 67.0181	Gallic acid	$C_7H_6O_5$	-0.91
9	7.262	131.0351	87.0456, 69.0359, 72.0002	2-Hydroxy-4-oxopentanoic acid	$C_5H_8O_4$	0.76
10	7.452	161.0456	93.0340, 89.0249, 71.0499	2-(2-Hydroxy-1-oxopropoxy)propionic acid	$C_6H_{10}O_5$	-0.33
11	8.03	129.0193	87.0452, 85.0295	unidentified		
12	8.267	131.0353	87.0454, 69.0339, 59.0125	Methylsuccinic acid	$C_5H_8O_4$	2.29
13	8.635	167.0352	123.0451, 93.0341, 65.0401	Isovanillic acid	$C_8H_8O_4$	-0.7
14	9.156	153.0194	109.0299, 108.0221, 65.0039	Protocatechuic acid	$C_7H_6O_4$	-0.44
15	9.914	353.0883	191.0564, 173.0460, 135.0456, 85.0299, 59.0144	Caffeoylquinic acid	$C_{16}H_{18}O_9$	-1.4
16	10.26	367.1039	193.0508, 149.0615, 134.0377, 117.0348, 89.0400	Feruloylquinic acid	$C_{17}H_{20}O_9$	-1.21
17	10.766	137.0246	119.0140, 108.0220, 81.0349, 65.0038, 53.0400	3,4-Dihydroxybenzaldehyde	$C_7H_6O_3$	-1.33
18	11.15	210.0777	124.0406, 94.0303, 66.0353	unidentified		
19	11.516	367.1044	193.0515, 191.0562, 173.0459, 134.0377, 111.0453, 93.0350	Feruloylquinic acid	$C_{17}H_{20}O_9$	-2.57
20	11.777	197.0458	153.0559, 121.0300, 78.0116	Syringic acid	$C_9H_{10}O_5$	-1.28
21	12.905	435.1294	345.0985, 315.0873, 273.0767, 209.0453, 167.0352, 125.0247	Nothofagin	$C_{21}H_{24}O_{10}$	0.62
22	13.27	163.0406	119.0507, 93.0351, 65.0399	*p*-Coumaric acid	$C_9H_8O_3$	-3.27
23	13.547	167.0341	152.0108, 123.0415, 108.0215, 91.0184, 65.0035, 51.0243	Vanillic acid	$C_8H_8O_4$	-5.39
24	14.144	243.0666	225.0551, 201.0553, 173.0607, 159.0455	Piceatannol	$C_{14}H_{12}O_4$	1.23
25	15.014	301.0357	227.0170, 149.0151, 64.9993	Tricetin	$C_{15}H_{10}O_7$	-1.08
26	15.426	485.1251	375.0868, 241.0504, 135.0451, 109.0296	Scirpusin B	$C_{28}H_{22}O_8$	1.86

No.	t_r (min)[a]	m/z, ion: [M-H]⁻	MS/MS	Identification	Formula	Error (ppm)
27	15.637	179.0715	135.0819, 65.0038	3-(3-Hydroxyphenyl)-2-methylpropionic acid	$C_{10}H_{12}O_3$	-0.74
28	16.49	469.1291	385.1054, 359.0985, 346.0822, 241.0523	Scirpusin A	$C_{28}H_{22}O_7$	-0.43
29	16.767	285.0408	133.0272, 65.0024	Kaempferol	$C_{15}H_{10}O_6$	-0.49
30	17.717	327.2171	291.1956, 211.1342, 171.1027, 137.0974	unidentified		
31	18.089	267.1601	233.1704, 205.1596, 179.1439, 97.0661	4,11,13,15-Tetrahydroridentin B	$C_{15}H_{24}O_4$	-0.37
32	18.642	329.2335	229.1447, 211.1341, 171.1025, 99.0816, 57.0345	(9*S*,10*R*,11*E*,13*S*)-9,10,13-trihydroxyoctadec-11-enoic acid	$C_{18}H_{34}O_5$	0.61
33	20.075	861.2446	759.2043, 685.1941, 601.1756, 175.0401	1-*O*-Acetyl-3-*O*,6-*O*-bis(3-methoxy-4-hydroxy-trans-cinnamoyl)-β-D-fructofuranosyl 3-*O*,4-*O*,6-*O*-triacetyl-α-D-glucopyranoside	$C_{40}H_{46}O_{21}$	1.49
34	22.413	423.1451	364.1304, 249.0399, 145.0294	Erythynone	$C_{24}H_{24}O_7$	-0.41
35	27.091	327.1814	239.2012, 124.0168	2-(((1-carboxy-6-methylheptyl)oxy)carbonyl)cyclohexane-1-carboxylic acid	$C_{17}H_{28}O_6$	-0.27

[a] retention time

Table S3. Tentative identification of phytochemicals in ECR identified by LC–MS/MS in positive mode

No.	t_r (min)[a]	m/z, ion: [M+H]+	MS/MS	Identification	Formula	Error (ppm)
1	2.688	104.1070	60.0804, 58.0647	(*R*)-5-aminopentan-2-ol	$C_5H_{13}NO$	-0.09
2	3.143	126.0549	108.0440, 53.0348	3-Aminobenzene-1,2-diol	$C_6H_7NO_2$	0.44
3	3.499	110.0602	94.0280, 82.0648, 67.0412, 55.0538	1-Methyl-2-pyrrolecarboxaldehyde	C_6H_7NO	-1.45
4	4.938	124.0427	106.0268, 96.0784, 80.0482, 78.0325, 53.0377	unidentified		
5	5.108	130.0496	84.0439, 56.0490	4-Oxo-L-proline	$C_5H_7NO_3$	2.07
6	6.359	146.0814	86.0596, 69.0330	4-Acetamidobutanoic acid	$C_6H_{11}NO_3$	1.37
7	6.981	199.1078	181.0960, 163.0857, 121.0755, 109.0752	unidentified		
8	7.145	145.0498	99.0429, 71.0487	(*E*)-2-Methylglutaconic acid	$C_6H_8O_4$	-1.83
9	7.649	144.0655	84.0439, 56.0491	unidentified		
10	8.178	127.0394	109.0279, 81.0330, 69.0331, 53.0384	1,2,3-Trihydroxybenzene	$C_6H_6O_3$	-3.38
11	8.42	208.1333	190.1220, 162.1270, 107.0849	Phenyl-Leucine	$C_{12}H_{17}NO_2$	-0.46
12	8.66	265.1295	162.0906, 112.0862, 70.0650	unidentified		
13	8.838	248.1645	174.1269, 144.0797, 120.0798	unidentified		
14	9.496	127.0391	53.0383	Maltol	$C_6H_6O_3$	-3.38
15	9.877	114.0914	96.0796, 69.0694, 55.0537	Cyclohexanone oxime	$C_6H_{11}NO$	-0.52
16	10.243	248.1646	230.1528, 146.0954	unidentified		
17	10.389	232.1695	214.1580, 158.0957	Rotundine A	$C_{15}H_{21}NO$	0.39
18	10.879	234.1852	216.1737, 160.1119	unidentified		
19	11.631	194.0812	98.0596, 70.0649	unidentified		
20	11.972	270.2062	252.1947, 234.1832, 189.1629, 133.0996	(2*E*,4*R*,5*R*)-4,5-Dihydroxy-1-(1-piperidinyl)-2-decen-1-one	$C_{15}H_{27}NO_3$	0.63
21	12.763	220.1694	203.1786, 177.1141, 81.0695	unidentified		
22	12.87	220.1694	204.1391, 177.1136, 162.0909, 107.0841, 81.0688	unidentified		
23	13.611	216.1744	160.1115, 146.0966, 132.0800	N-benzyl-N-but-3-enylbut-3-en-1-amine	$C_{15}H_{21}N$	1.28
24	15.755	302.1960, [M+NH4]+	285.1682, 123.0798	Dimethyl (3-oxo-2-pentylcyclopentyl)malonate	$C_{15}H_{24}O_5$	0.66
25	16.789	287.0548	207.1351, 153.0176, 68.9969	Kaempferol	$C_{15}H_{10}O_6$	0.75
26	17.401	153.1272	109.1007, 67.0539	Camphor	$C_{10}H_{16}O$	1.25

No.	t_r (min)[a]	m/z, ion: $[M+H]^+$	MS/MS	Identification	Formula	Error (ppm)
27	17.959	235.1692	217.1580, 175.1473, 157.1002, 119.0849, 81.0694	Procurcumenol	$C_{15}H_{22}O_2$	0.24
28	19.556	239.1641	221.1526, 193.1577, 175.1475, 123.0796, 69.0696	3-hydroxytetradeca-4,7,10-trienoic acid	$C_{14}H_{22}O_3$	0.3
29	20.94	237.1845	219.1733, 201.1629, 159.1160, 137.0957, 123.0797	unidentified		
30	22.642	203.1793	147.1161, 119.0850, 105.0693, 55.0536	Curcumene	$C_{15}H_{22}$	0.63

[a]retention time

MS/MS Fragmentation
Negative mode

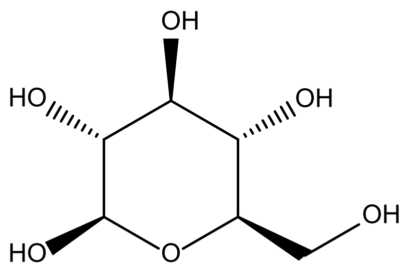

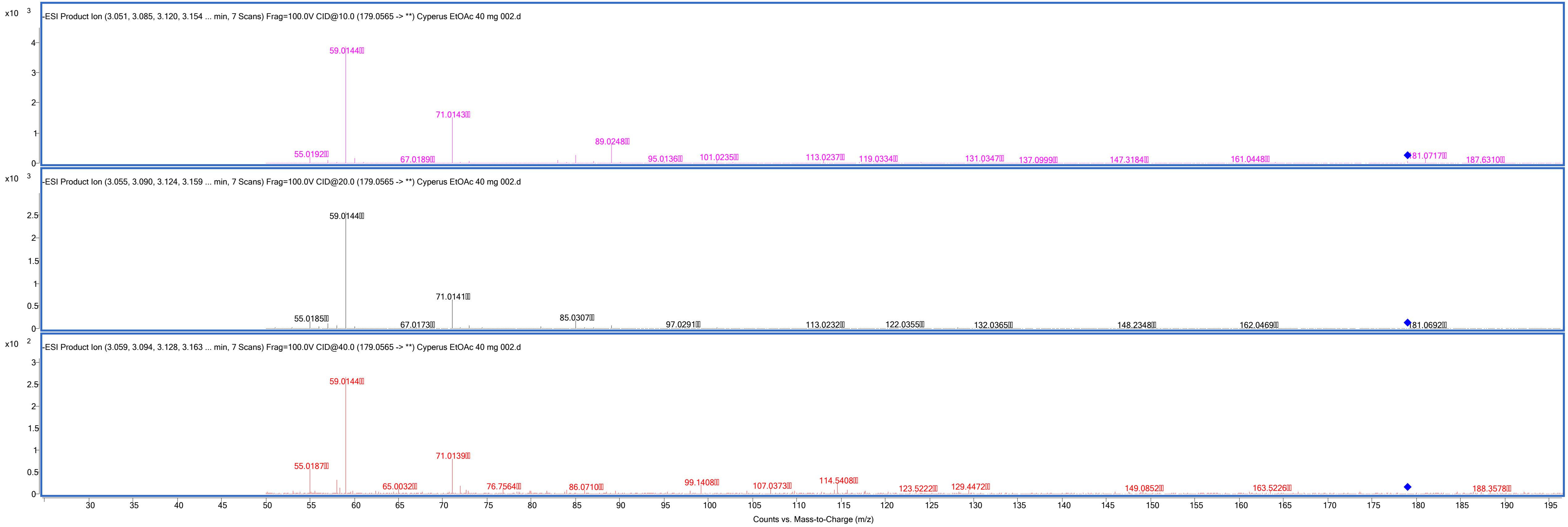

Figure S3A.1. The ESI-MS/MS fragmentation spectra of compound No 1 at m/z 179.0570 at various collision energies (10, 20, 40 eV) in the negative ionization mode.

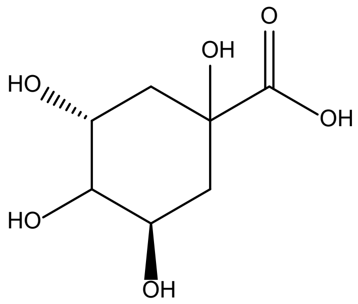

Quinic acid

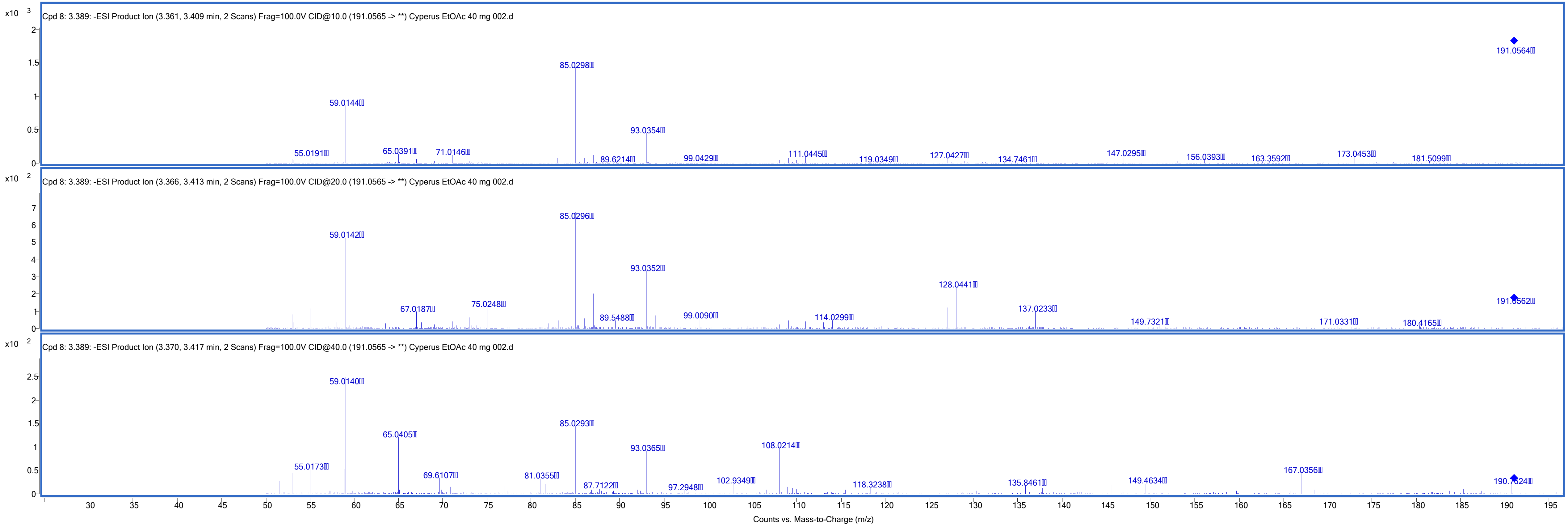

Figure S3A.2. The ESI-MS/MS fragmentation spectra of compound No 2 at m/z 191.0570 at various collision energies (10, 20, 40 eV) in the negative ionization mode.

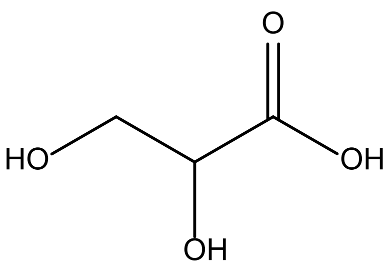

Glyceric acid

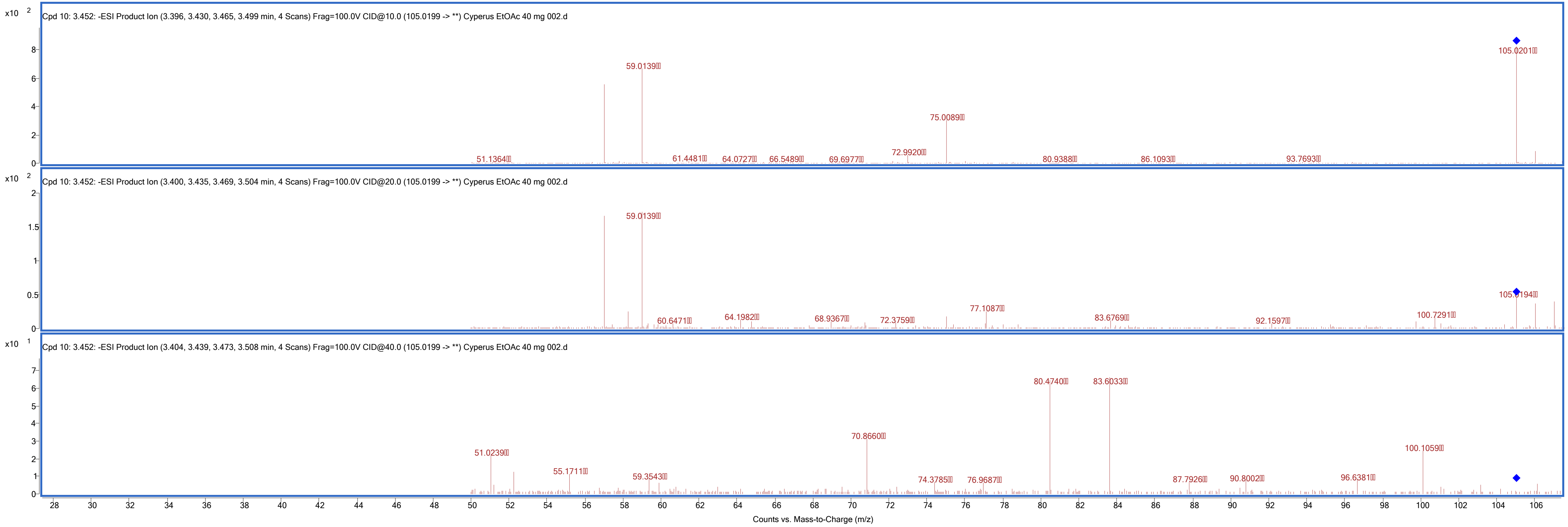

Figure S3A.3. The ESI-MS/MS fragmentation spectra of compound No 3 at m/z 105.0197 at various collision energies (10, 20, 40 eV) in the negative ionization mode.

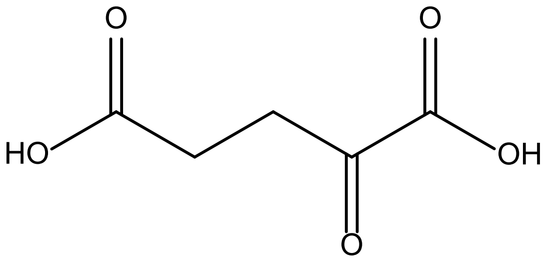

Oxoglutaric acid

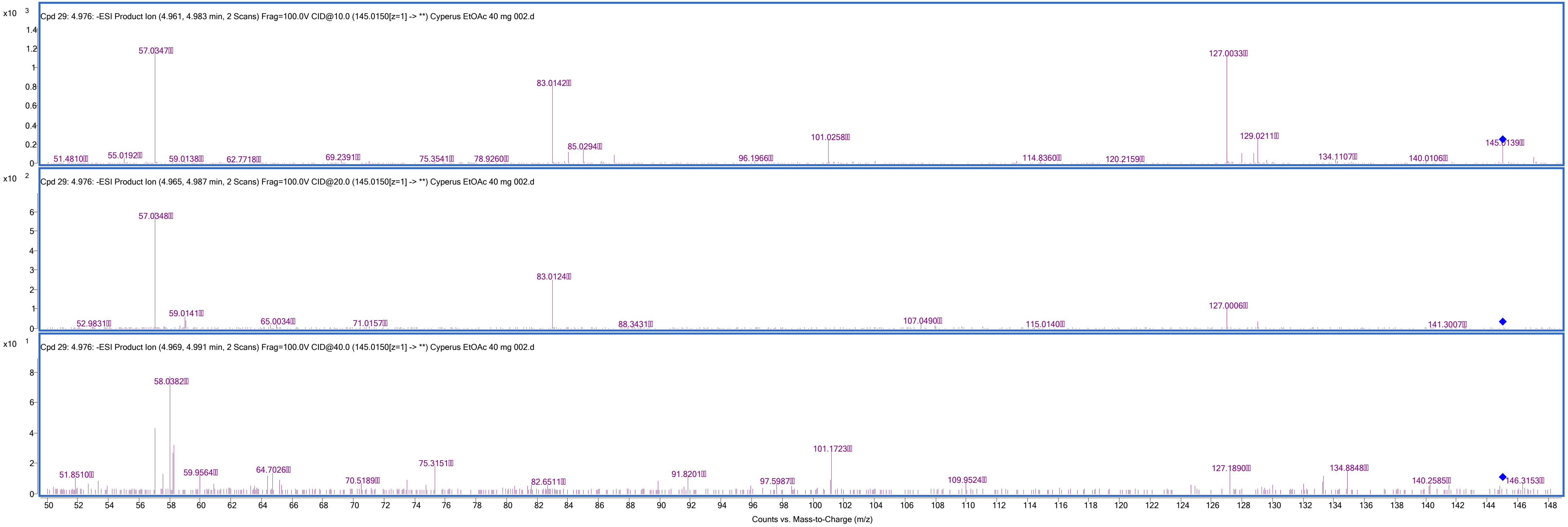

Figure S3A.4. The ESI-MS/MS fragmentation spectra of compound No 4 at m/z 145.0154 at various collision energies (10, 20, 40 eV) in the negative ionization mode.

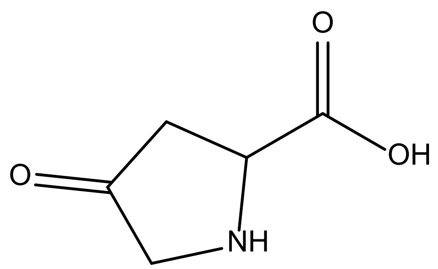

4-Oxo-L-proline

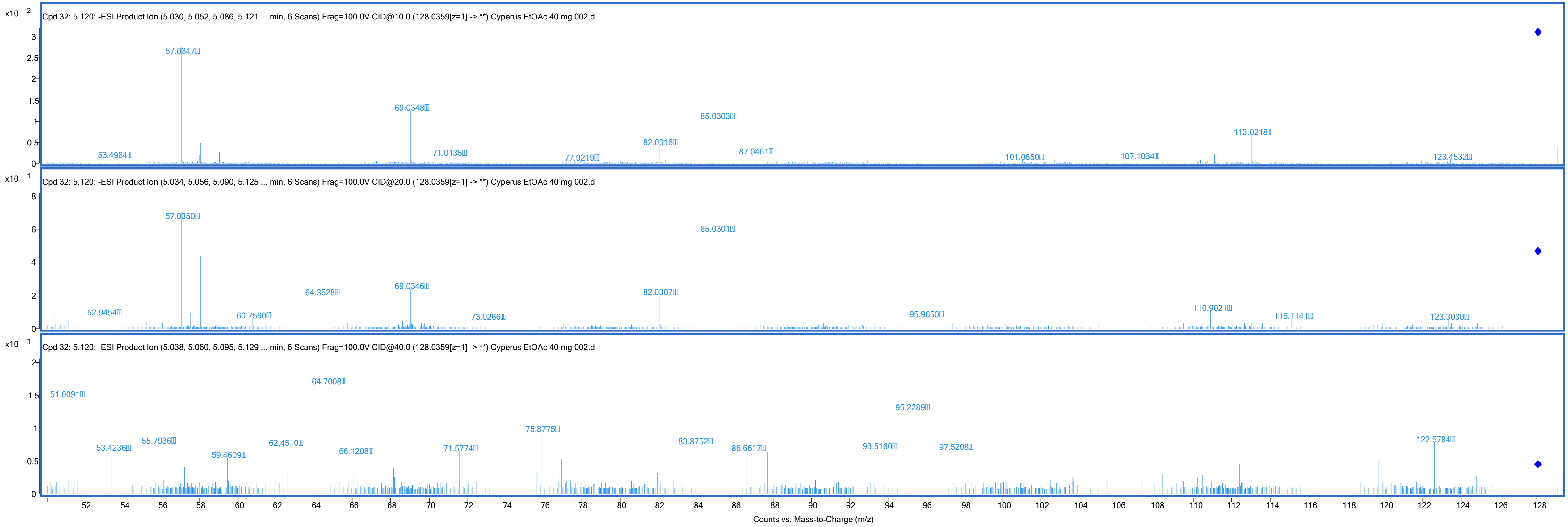

Figure S3A.5. The ESI-MS/MS fragmentation spectra of compound No 5 at m/z 128.0355 at various collision energies (10, 20, 40 eV) in the negative ionization mode.

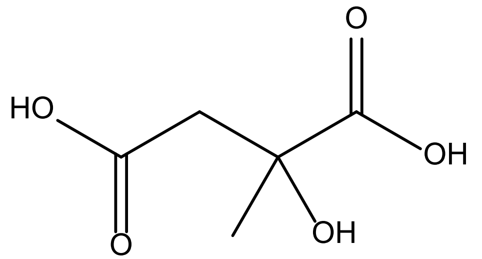

Citramalic acid isomer

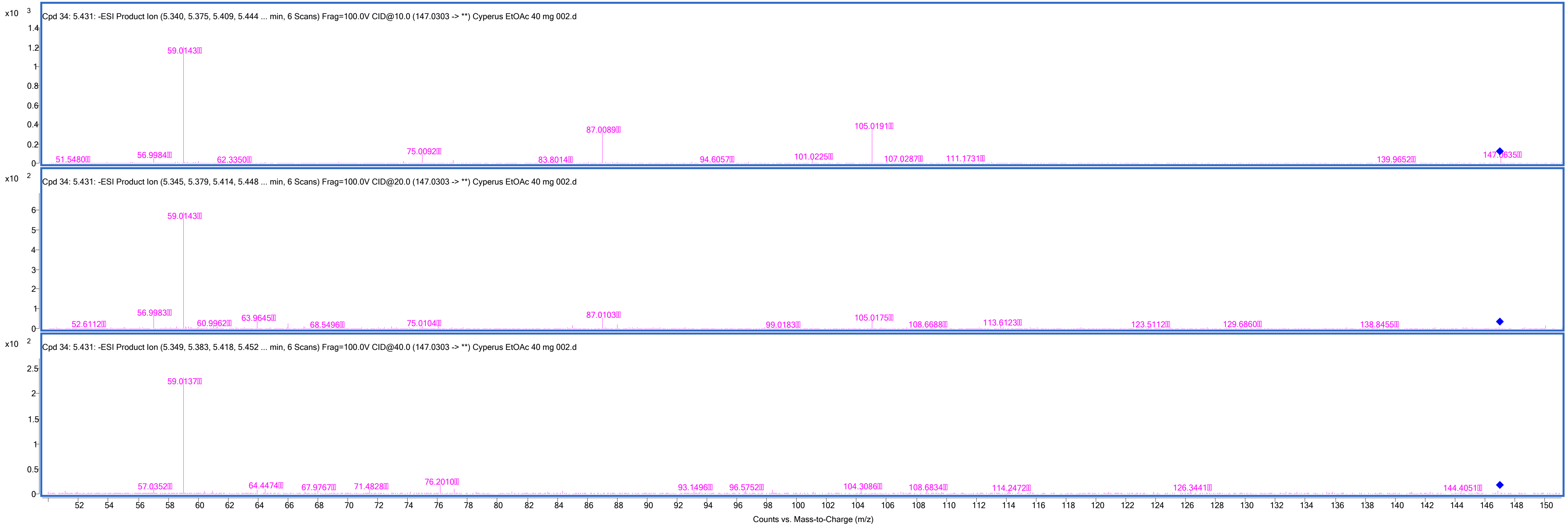

Figure S3A.6. The ESI-MS/MS fragmentation spectra of compound No 6 at m/z 147.0304 at various collision energies (10, 20, 40 eV) in the negative ionization mode.

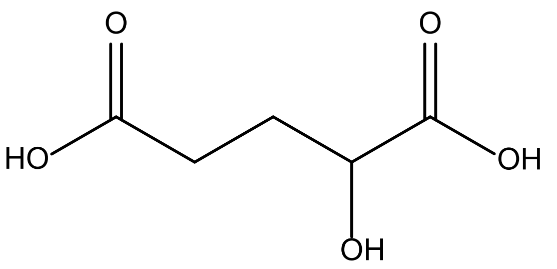

2-Hydroxyglutaric acid

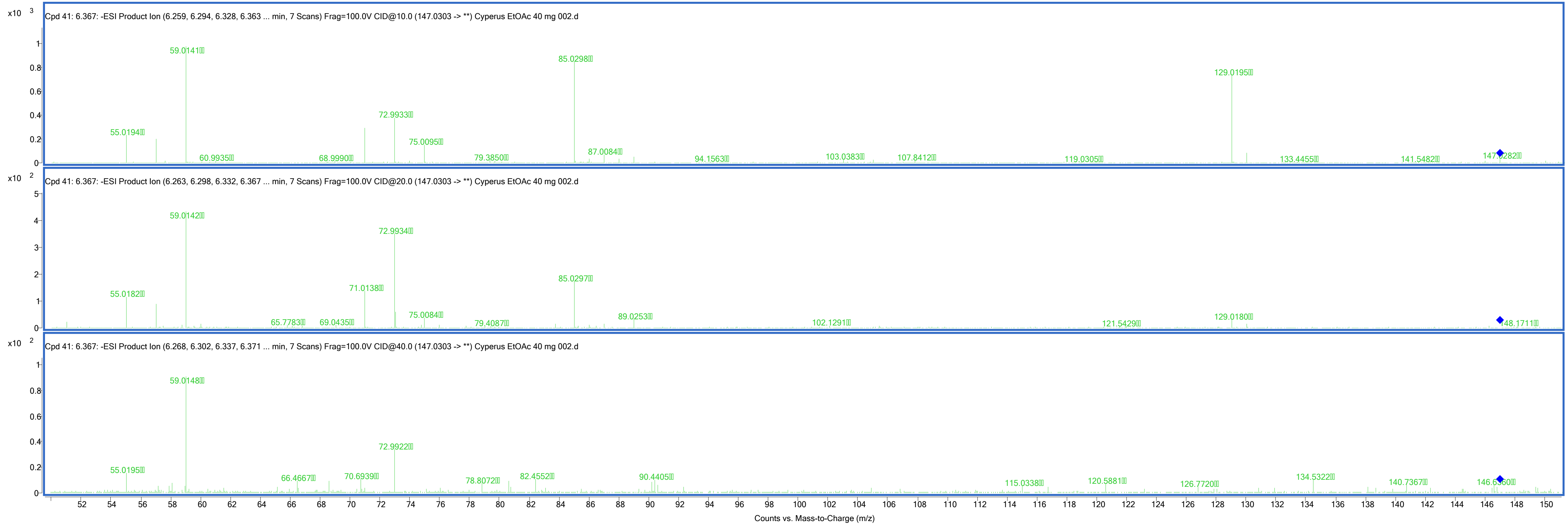

Figure S3A.7. The ESI-MS/MS fragmentation spectra of compound No 7 at m/z 147.0301 at various collision energies (10, 20, 40 eV) in the negative ionization mode.

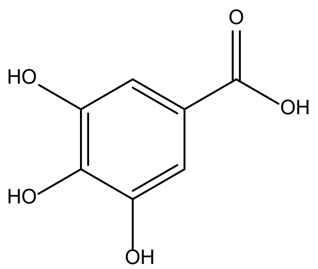

Gallic acid

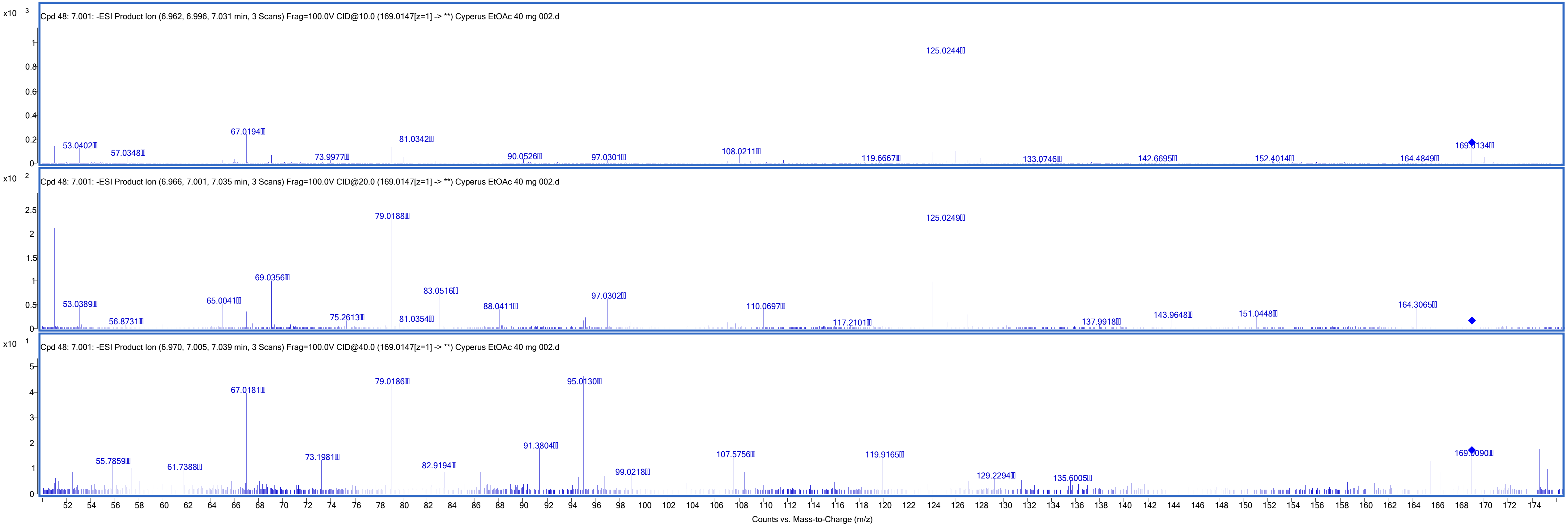

Figure S3A.8. The ESI-MS/MS fragmentation spectra of compound No 8 at m/z 169.0144 at various collision energies (10, 20, 40 eV) in the negative ionization mode.

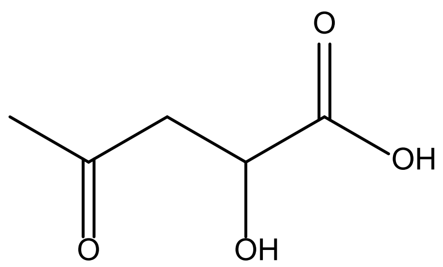

2-Hydroxy-4-oxopentanoic acid

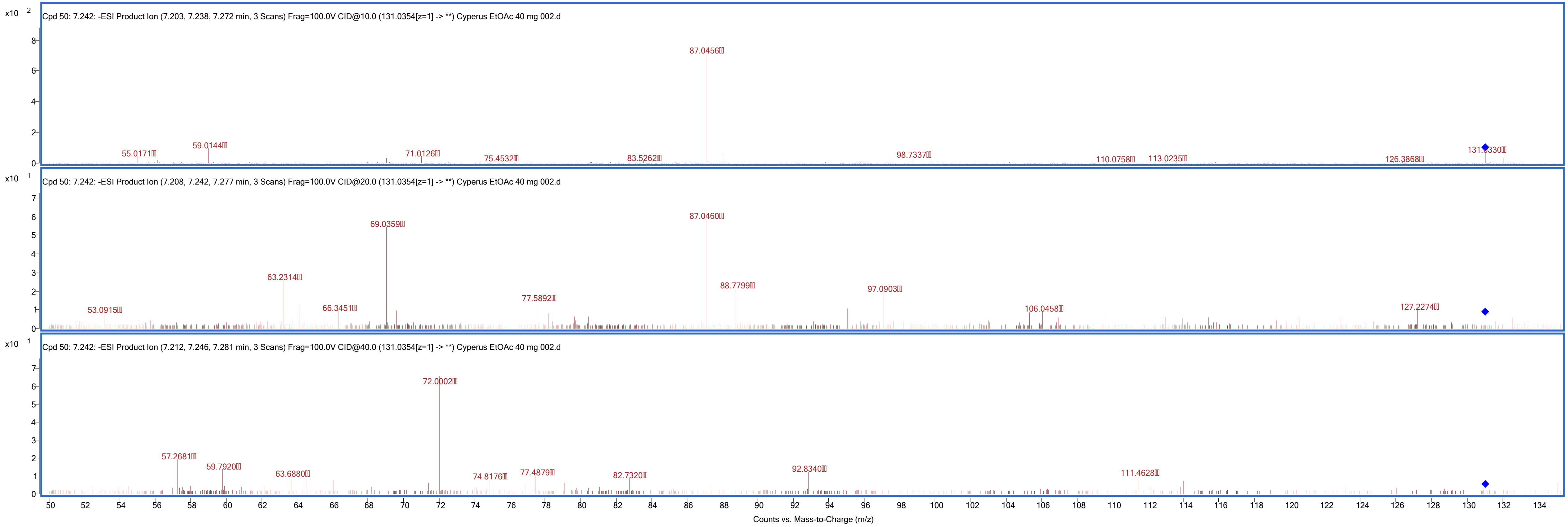

Figure S3A.9. The ESI-MS/MS fragmentation spectra of compound No 9 at m/z 131.0351 at various collision energies (10, 20, 40 eV) in the negative ionization mode.

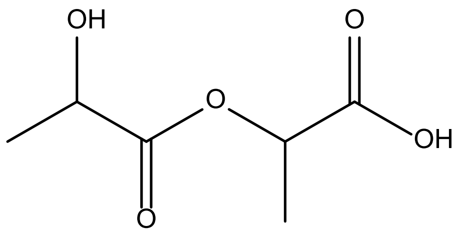

2-(2-Hydroxy-1-oxopropoxy)propionic acid

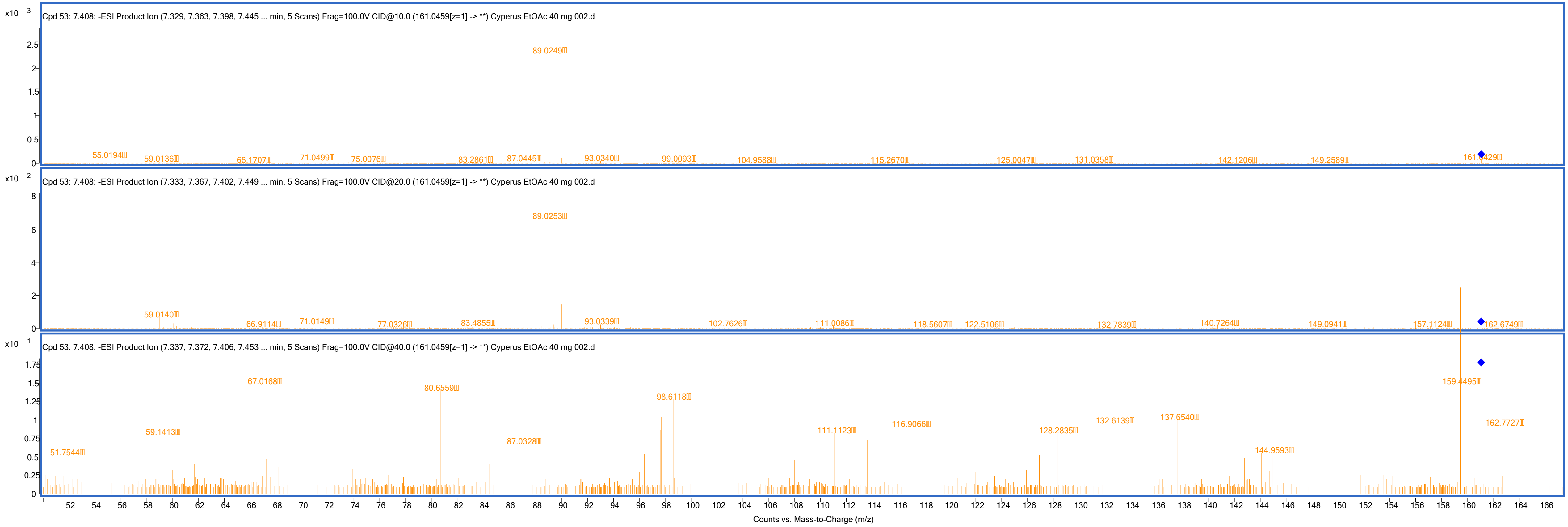

Figure S3A.10. The ESI-MS/MS fragmentation spectra of compound No 10 at m/z 161.0456 at various collision energies (10, 20, 40 eV) in the negative ionization mode.

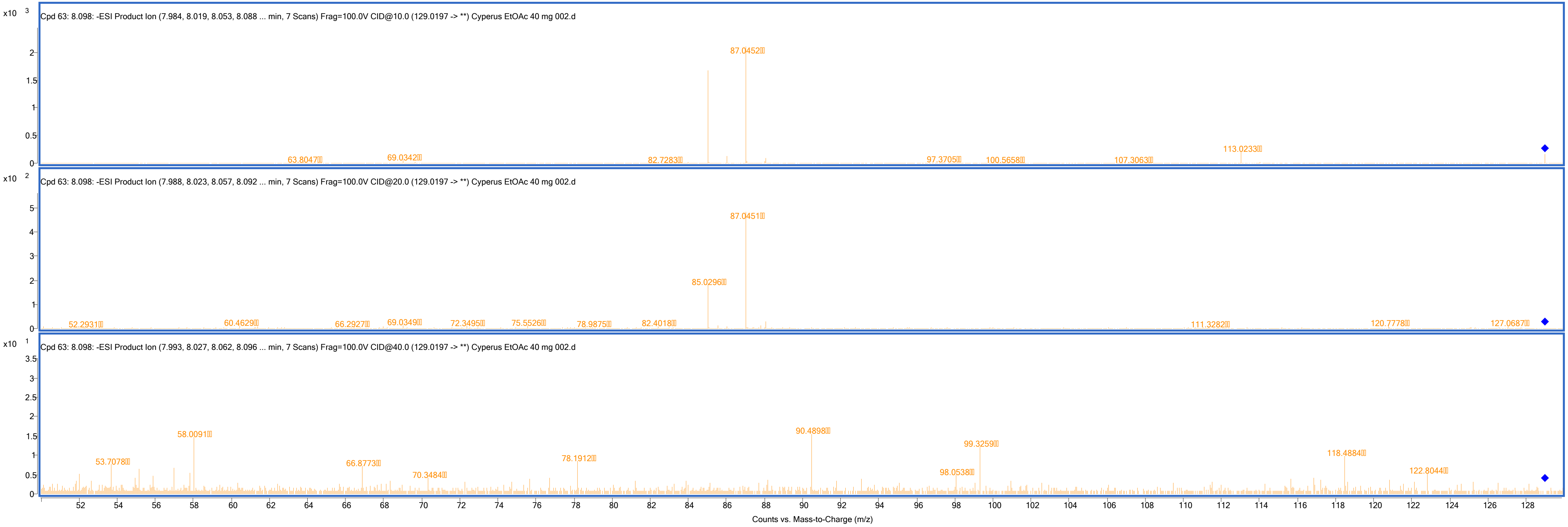

Figure S3A.11. The ESI-MS/MS fragmentation spectra of compound No 11 at m/z 129.0193 at various collision energies (10, 20, 40 eV) in the negative ionization mode.

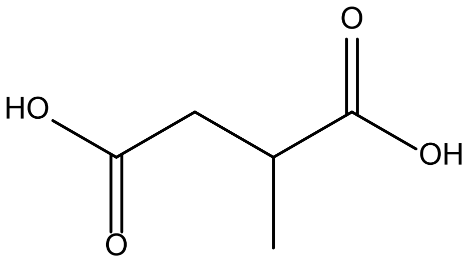

Methylsuccinic acid

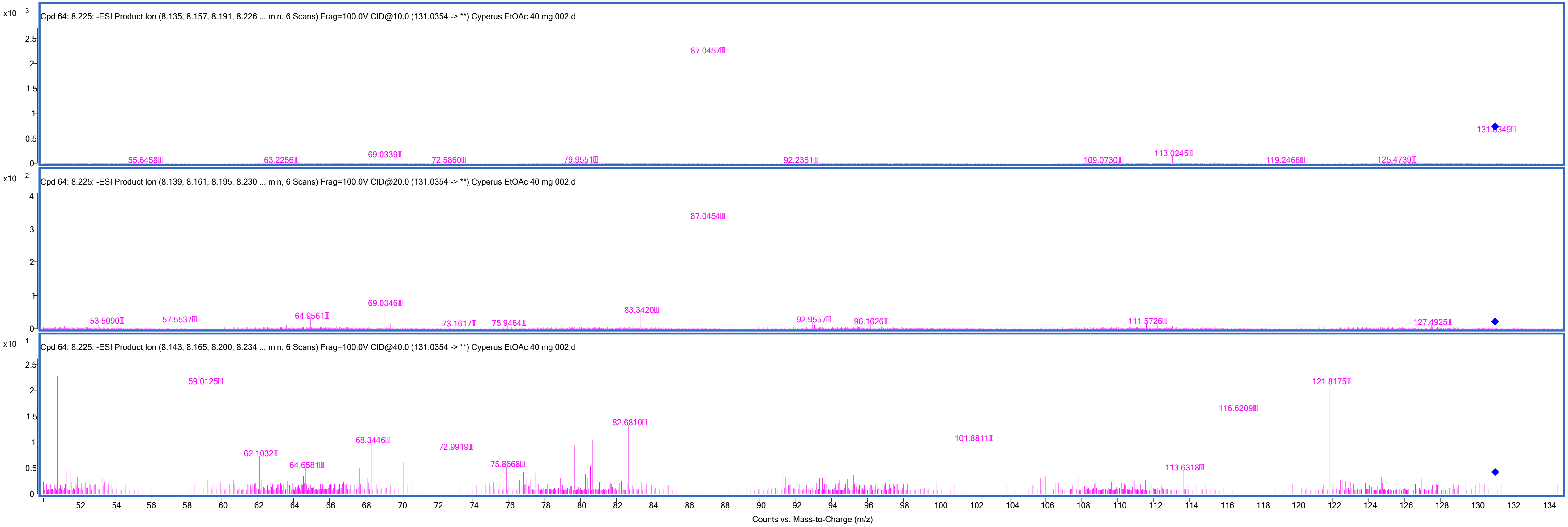

Figure S3A.12. The ESI-MS/MS fragmentation spectra of compound No 12 at m/z 131.0353 at various collision energies (10, 20, 40 eV) in the negative ionization mode.

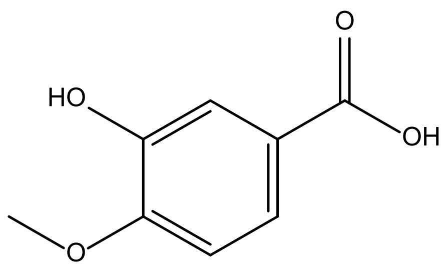

Isovanillic acid

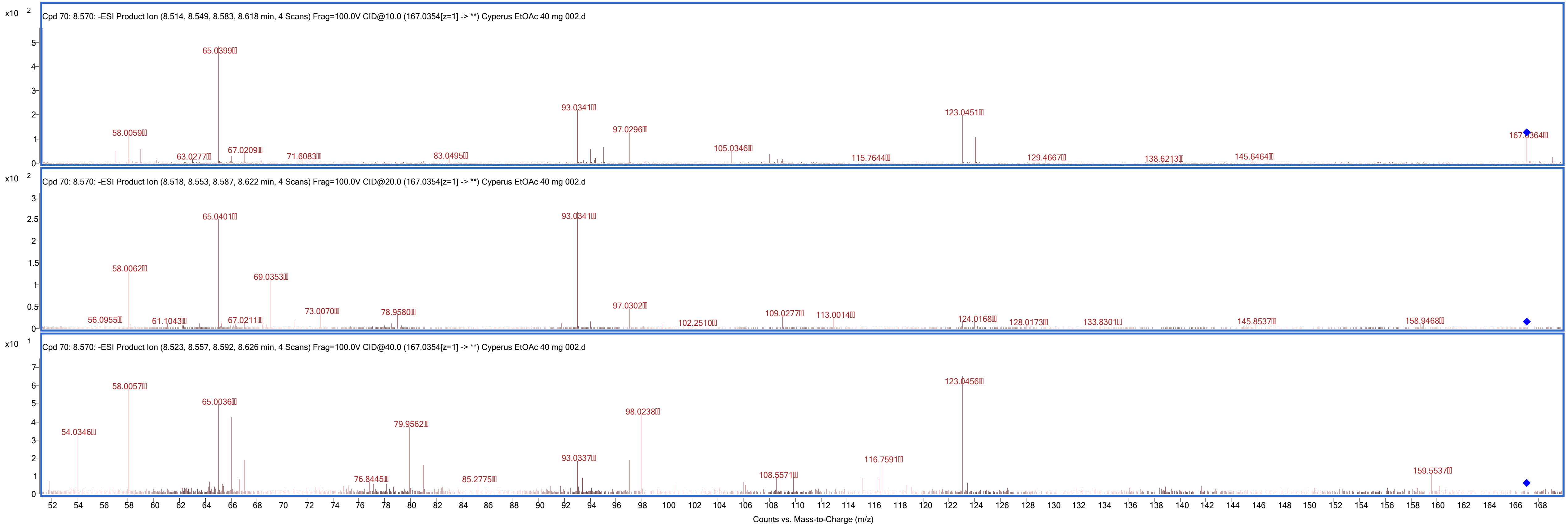

Figure S3A.13. The ESI-MS/MS fragmentation spectra of compound No 13 at m/z 167.0352 at various collision energies (10, 20, 40 eV) in the negative ionization mode.

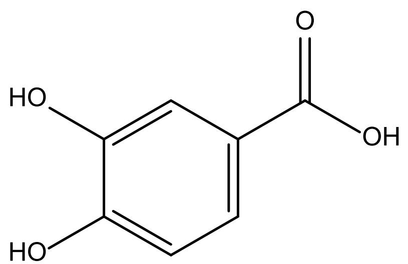

Protocatechuic acid

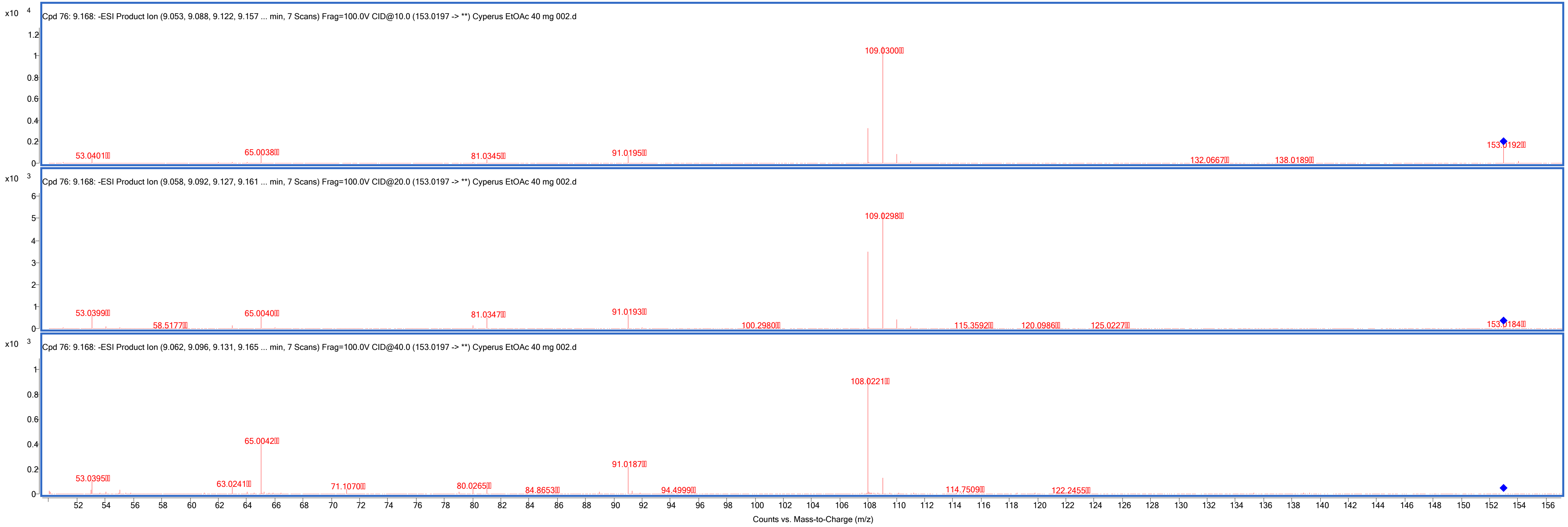

Figure S3A.14. The ESI-MS/MS fragmentation spectra of compound No 14 at m/z 153.0194 at various collision energies (10, 20, 40 eV) in the negative ionization mode.

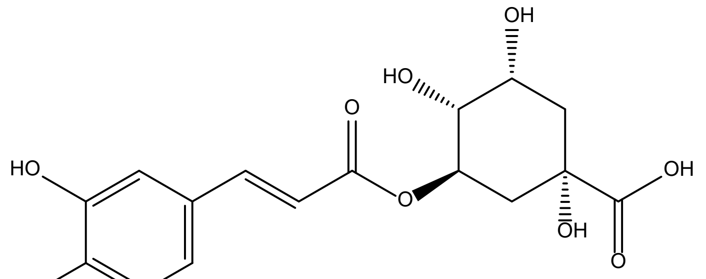

Caffeoylquinic acid

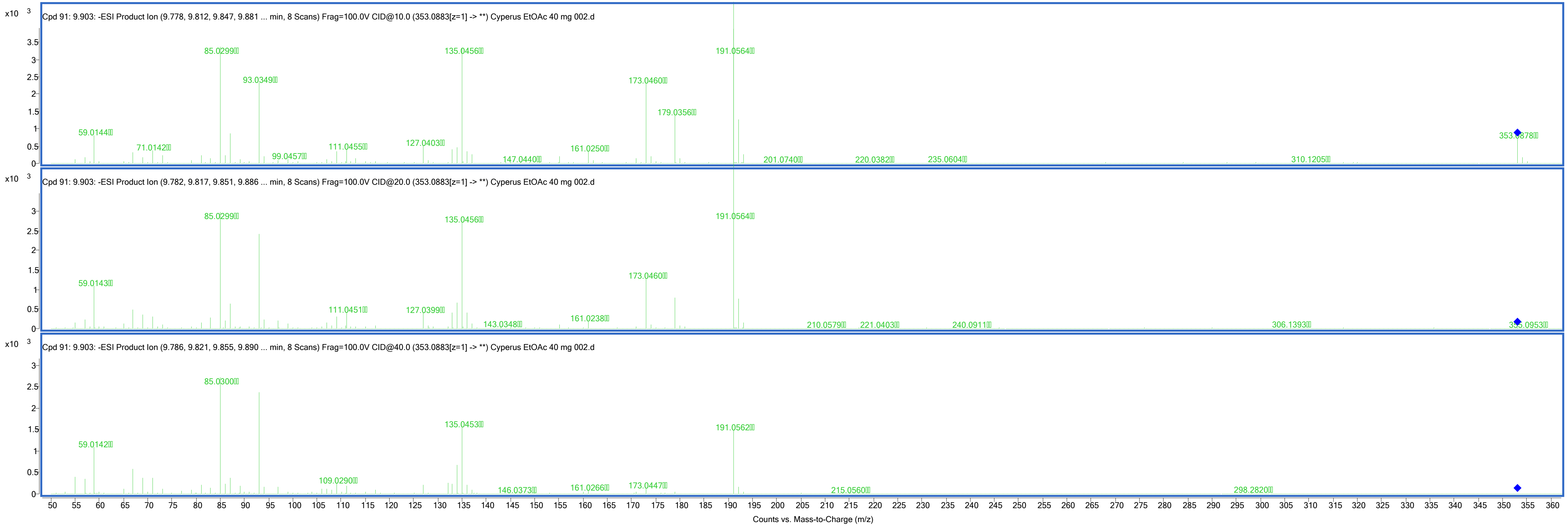

Figure S3A.15. The ESI-MS/MS fragmentation spectra of compound No 15 at m/z 353.0883 at various collision energies (10, 20, 40 eV) in the negative ionization mode.

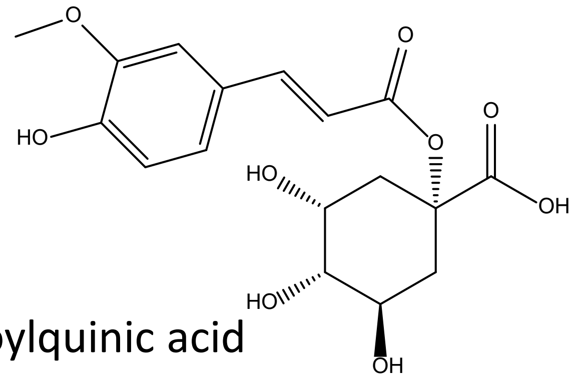

Feruloylquinic acid

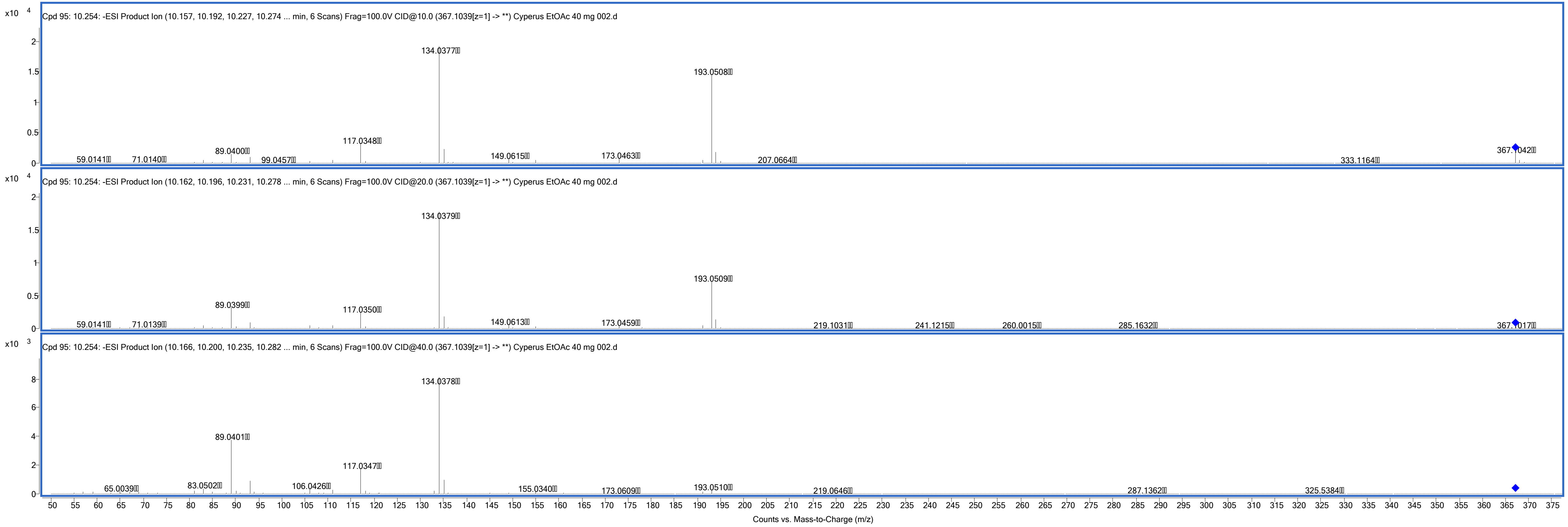

Figure S3A.16. The ESI-MS/MS fragmentation spectra of compound No 16 at m/z 367.1039 at various collision energies (10, 20, 40 eV) in the negative ionization mode.

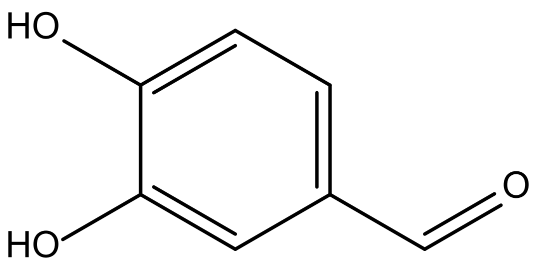

3,4-Dihydroxybenzaldehyde

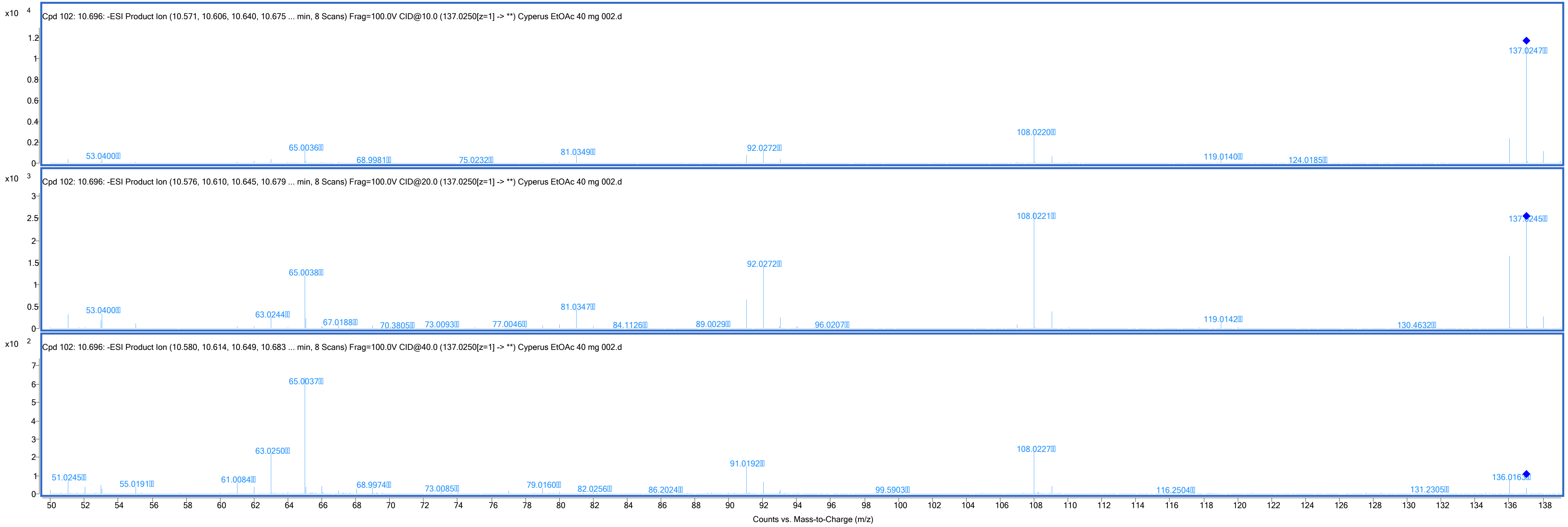

Figure S3A.17. The ESI-MS/MS fragmentation spectra of compound No 17 at m/z 137.0246 at various collision energies (10, 20, 40 eV) in the negative ionization mode.

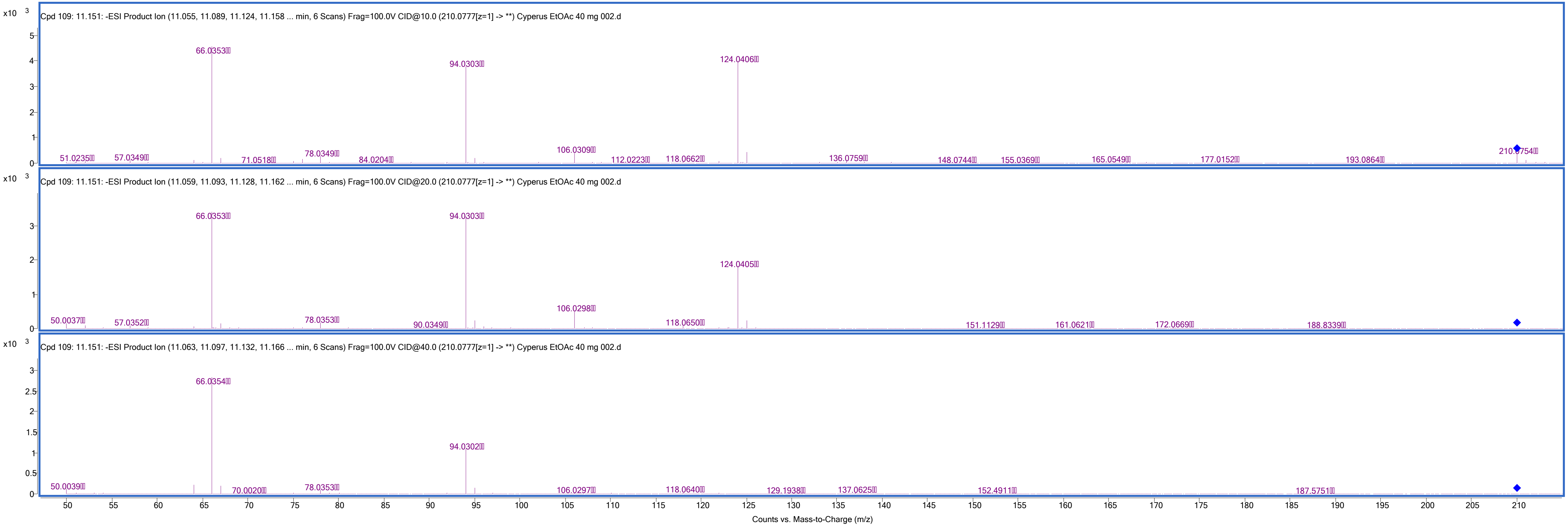

Figure S3A.18. The ESI-MS/MS fragmentation spectra of compound No 18 at m/z 210.0777 at various collision energies (10, 20, 40 eV) in the negative ionization mode.

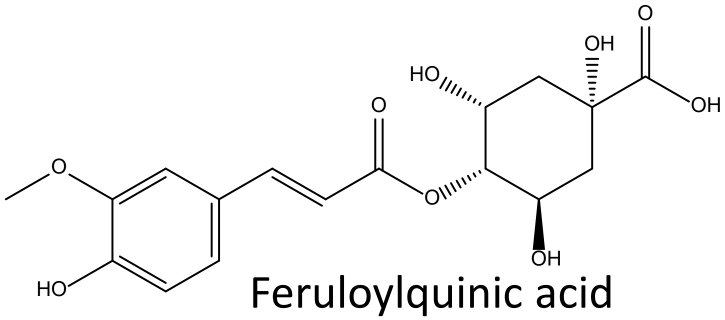

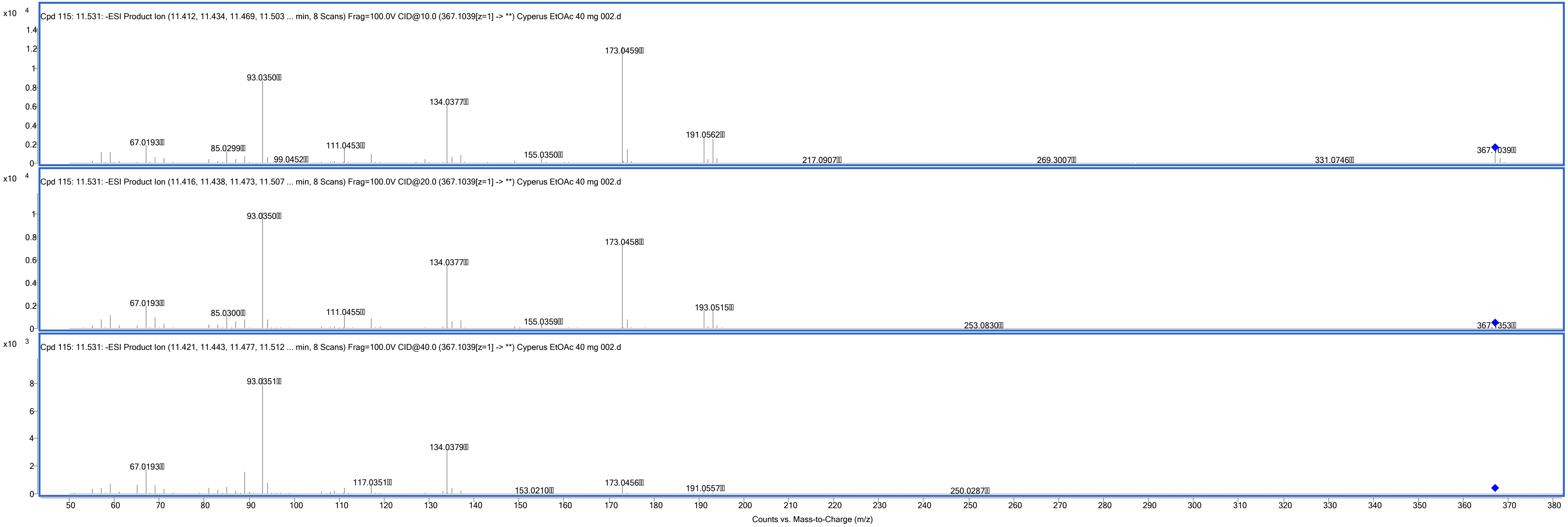

Figure S3A.19. The ESI-MS/MS fragmentation spectra of compound No 19 at m/z 367.1044 at various collision energies (10, 20, 40 eV) in the negative ionization mode.

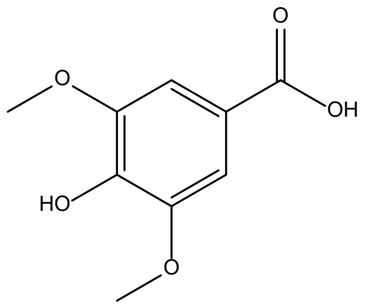

Syringic acid

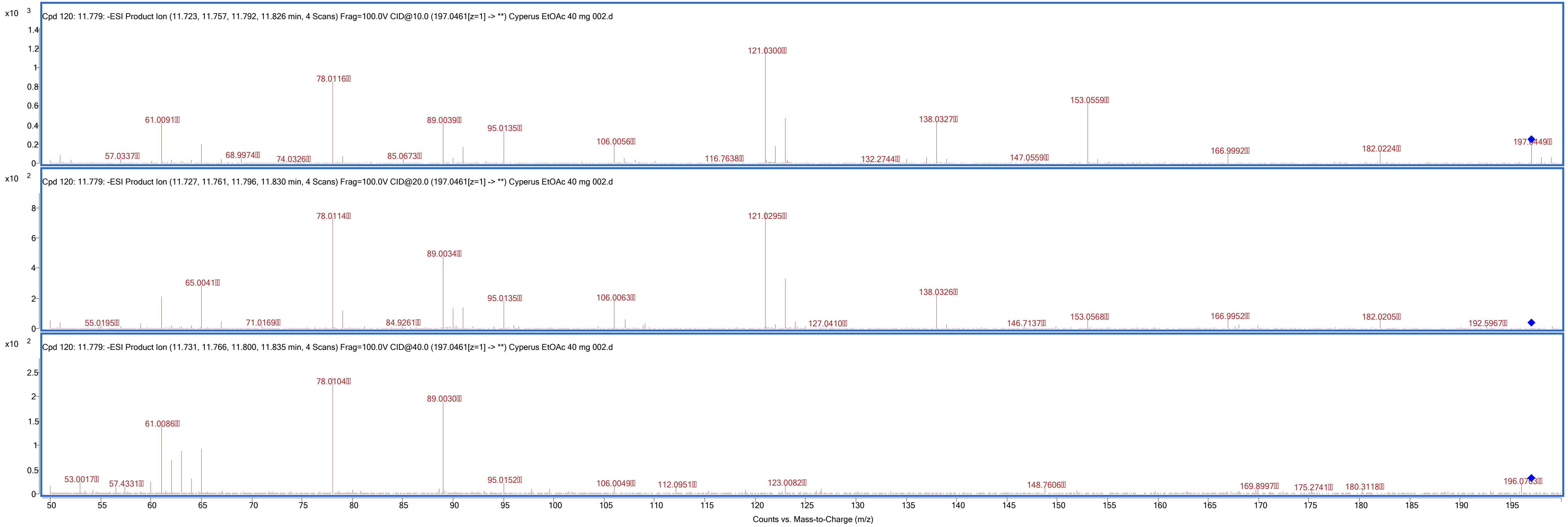

Figure S3A.20. The ESI-MS/MS fragmentation spectra of compound No 20 at m/z 197.0458 at various collision energies (10, 20, 40 eV) in the negative ionization mode.

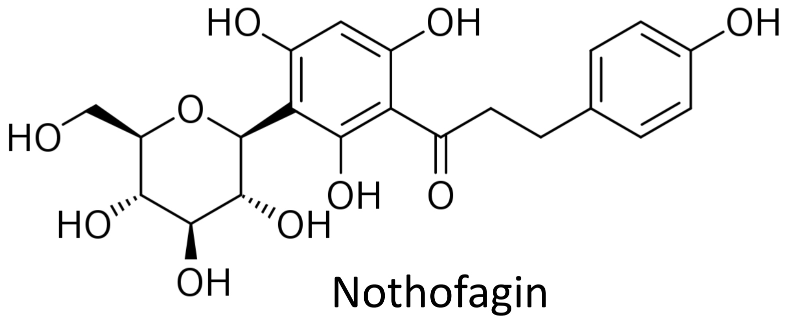

Nothofagin

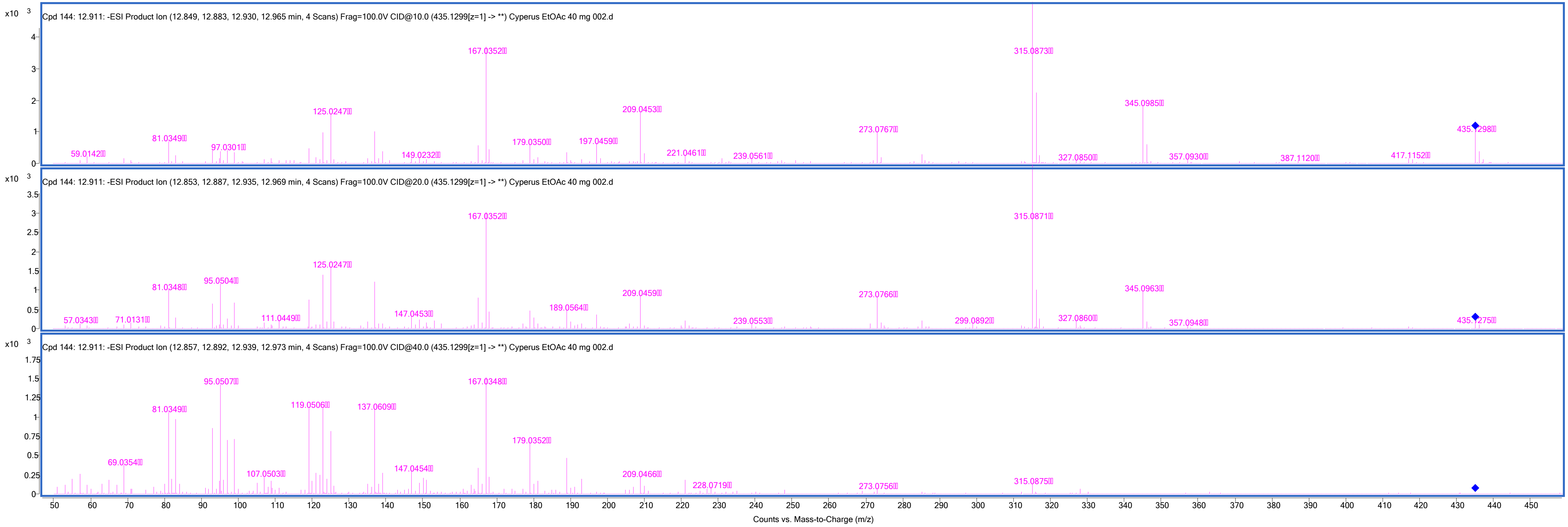

Figure S3A.21. The ESI-MS/MS fragmentation spectra of compound No 21 at m/z 435.1294 at various collision energies (10, 20, 40 eV) in the negative ionization mode.

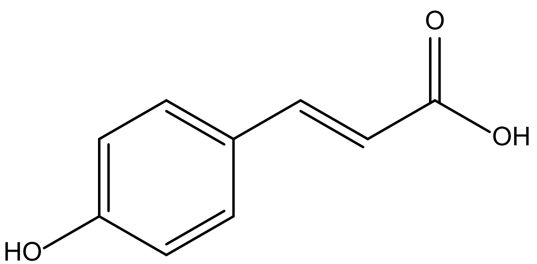

p-Coumaric acid

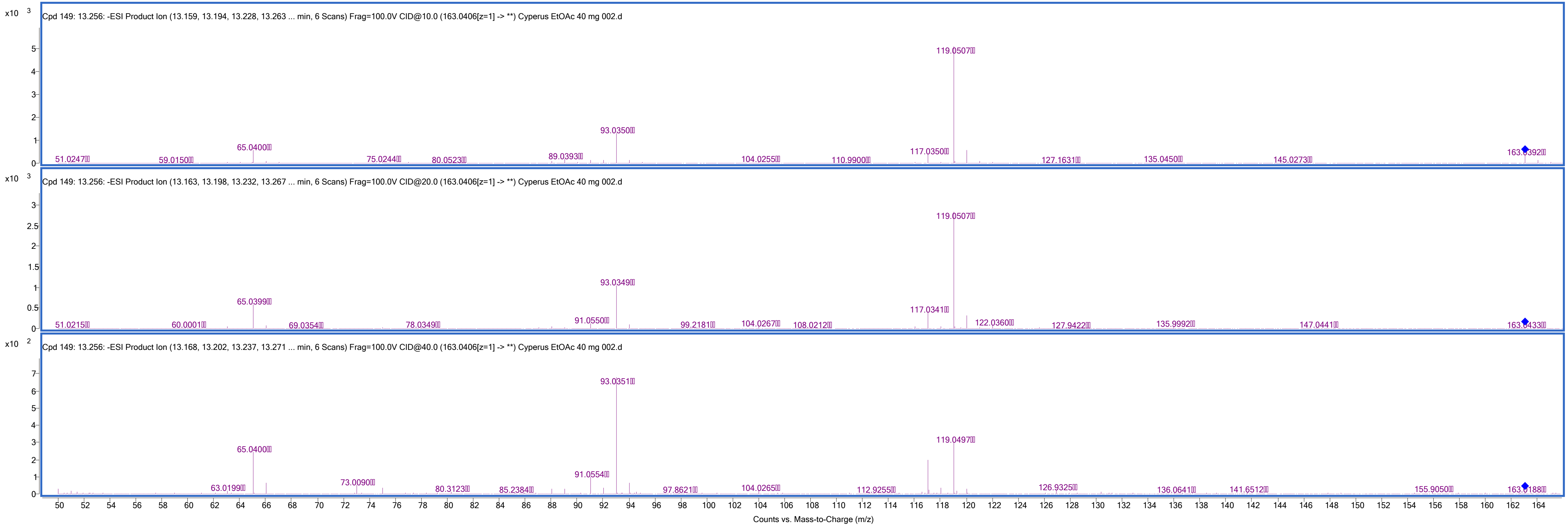

Figure S3A.22. The ESI-MS/MS fragmentation spectra of compound No 22 at m/z 163.0406 at various collision energies (10, 20, 40 eV) in the negative ionization mode.

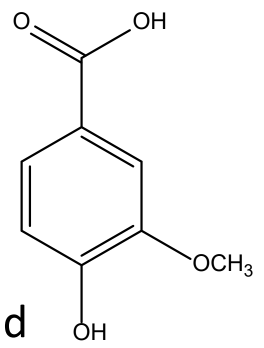

Vanillic acid

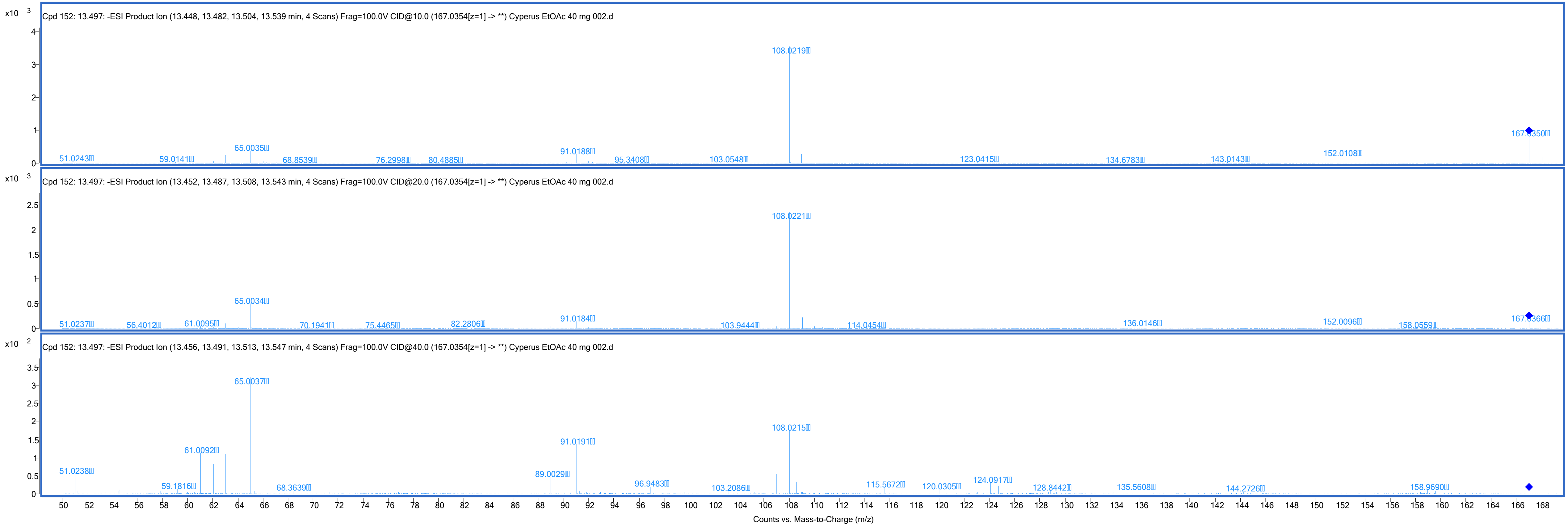

Figure S3A.23. The ESI-MS/MS fragmentation spectra of compound No 23 at m/z 167.0341 at various collision energies (10, 20, 40 eV) in the negative ionization mode.

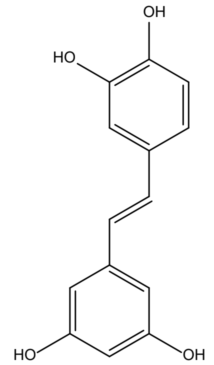

Piceatannol

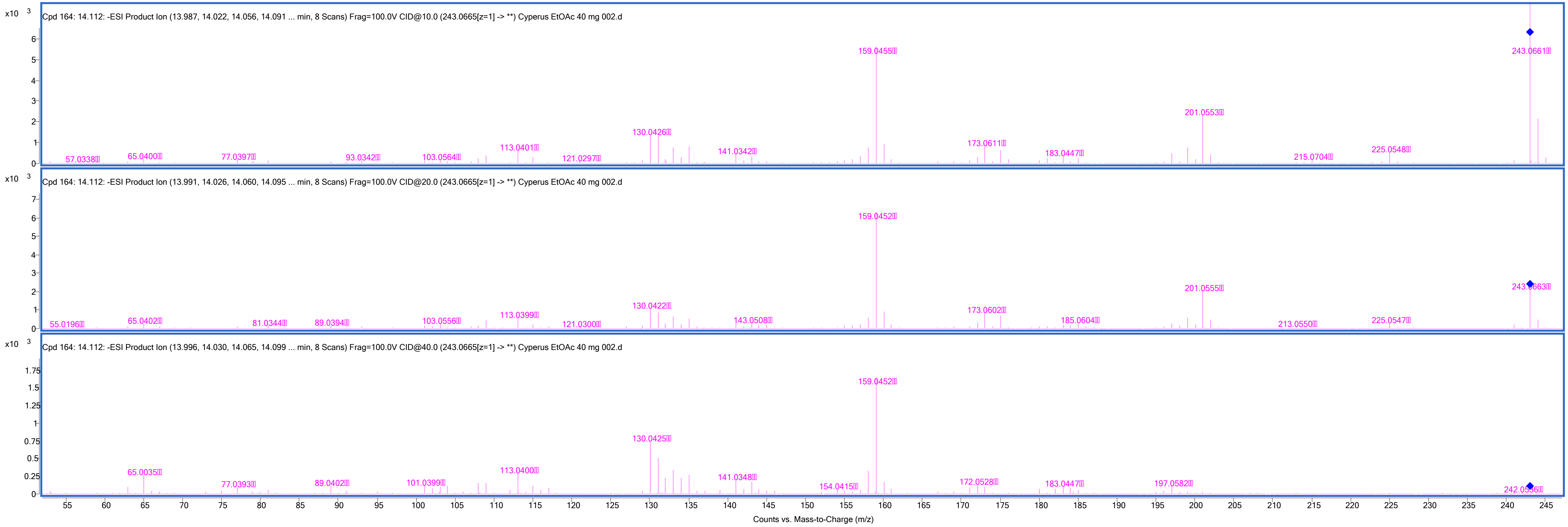

Figure S3A.24. The ESI-MS/MS fragmentation spectra of compound No 24 at m/z 243.0666 at various collision energies (10, 20, 40 eV) in the negative ionization mode.

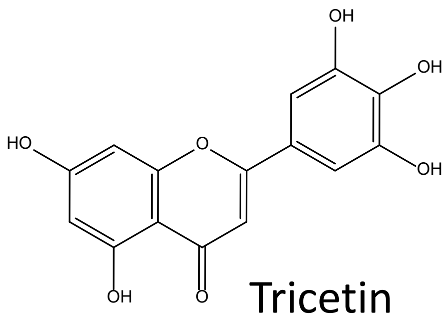

Tricetin

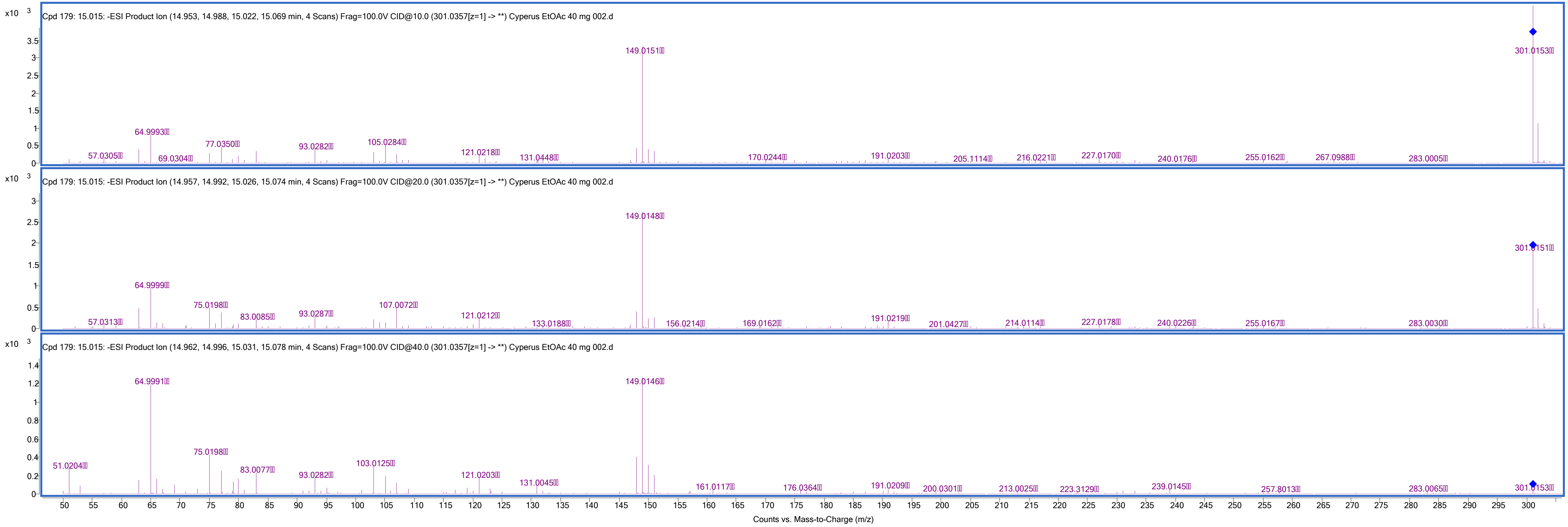

Figure S3A.25. The ESI-MS/MS fragmentation spectra of compound No 25 at m/z 301.0357 at various collision energies (10, 20, 40 eV) in the negative ionization mode.

Scirpusin B

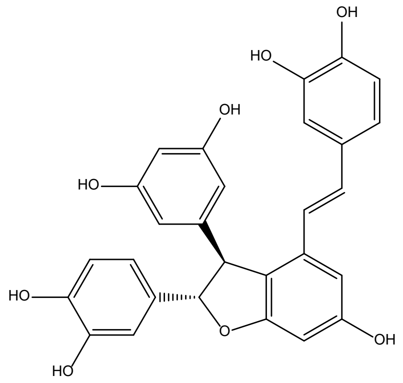

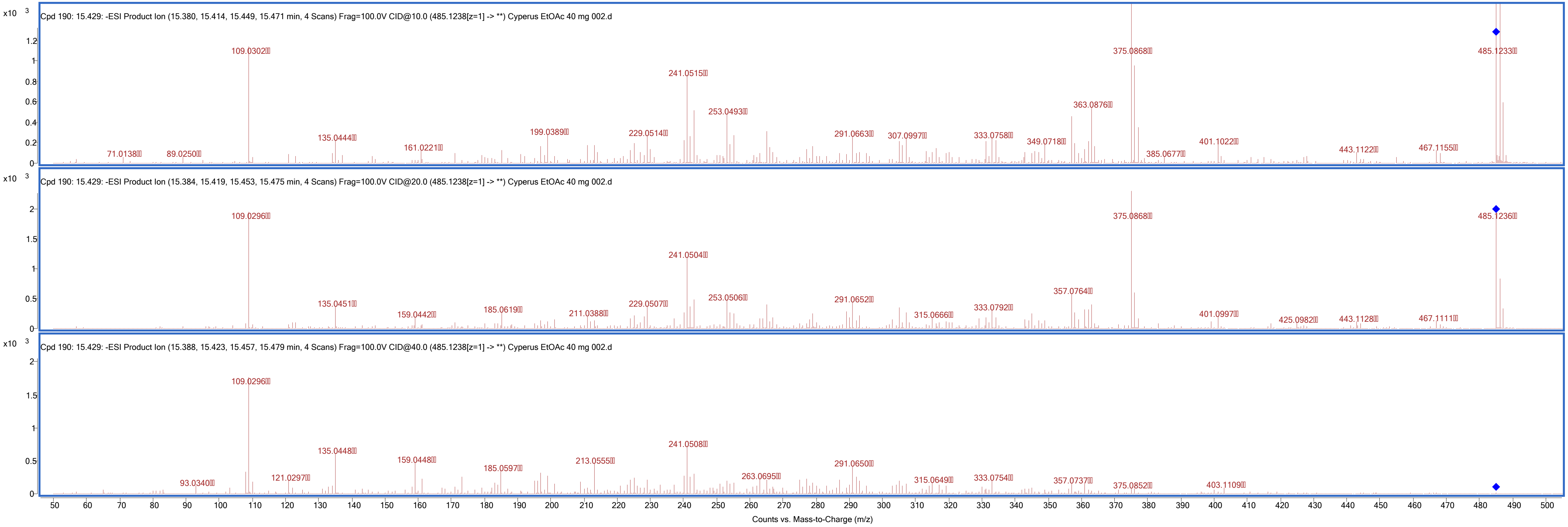

Figure S3A.26. The ESI-MS/MS fragmentation spectra of compound No 26 at m/z 485.1251 at various collision energies (10, 20, 40 eV) in the negative ionization mode.

Figure S3A.27. The purpose of fragmentation of scirpusin B

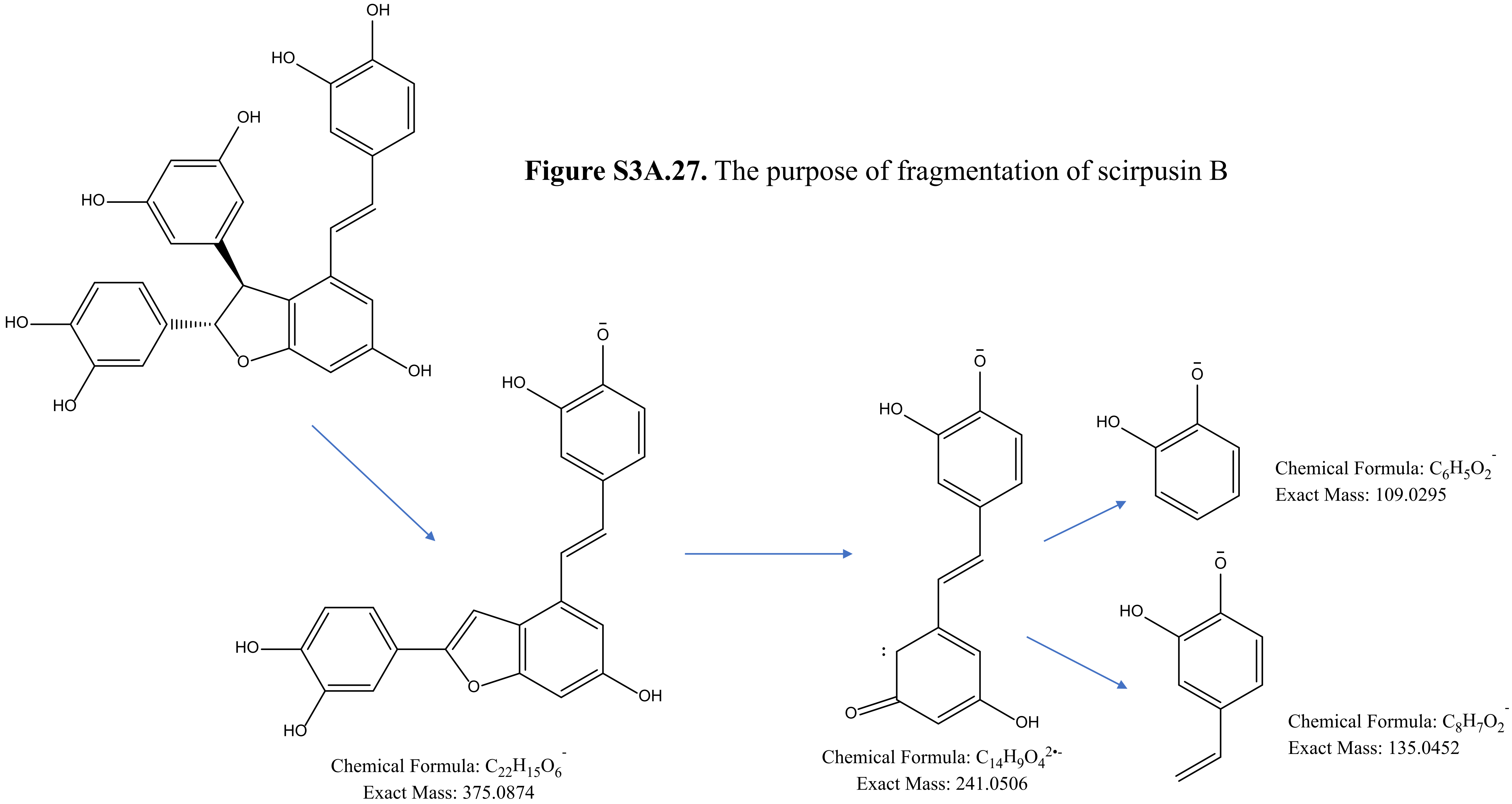

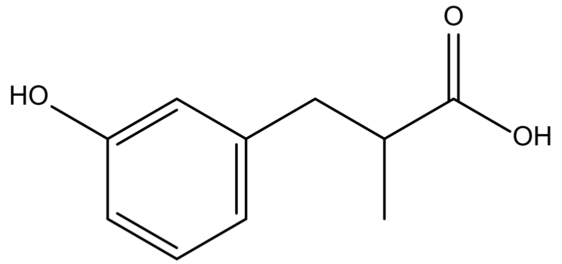

3-(3-Hydroxyphenyl)-2-methylpropionic acid

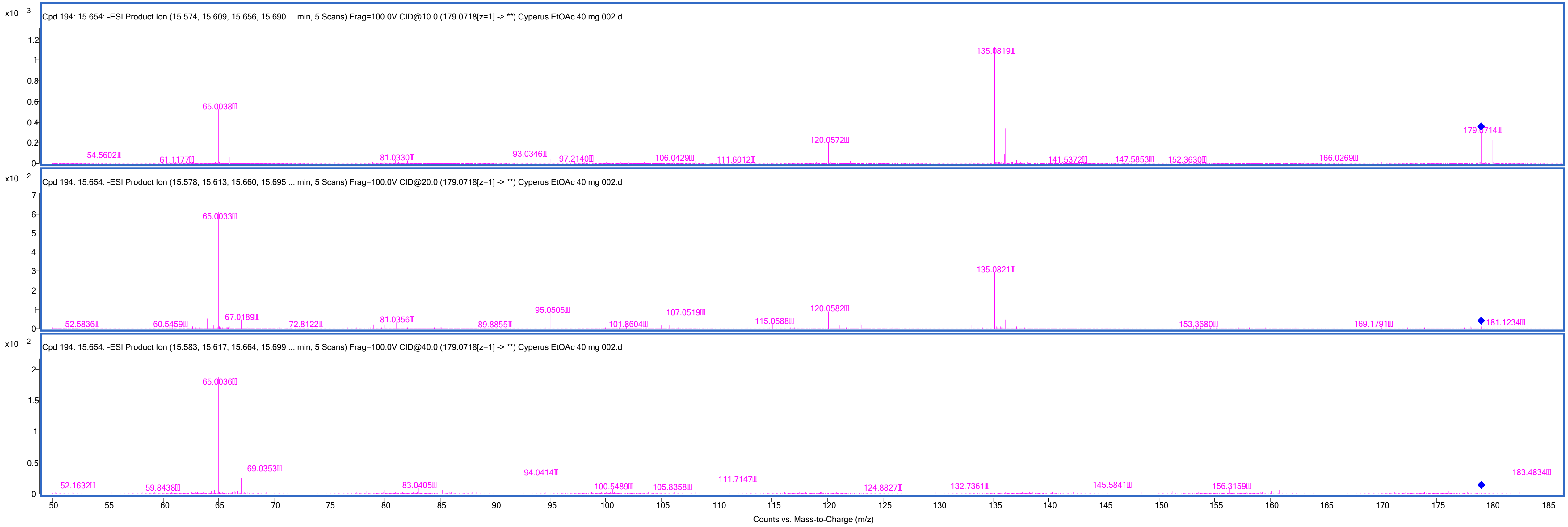

Figure S3A.28. The ESI-MS/MS fragmentation spectra of compound No 27 at m/z 179.0715 at various collision energies (10, 20, 40 eV) in the negative ionization mode.

Scirpusin A

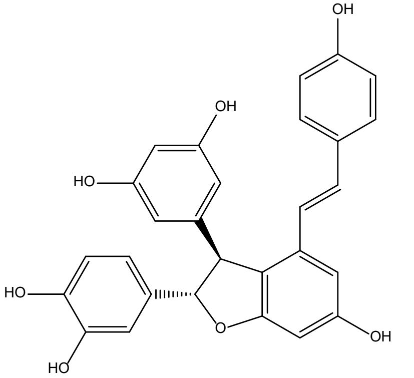

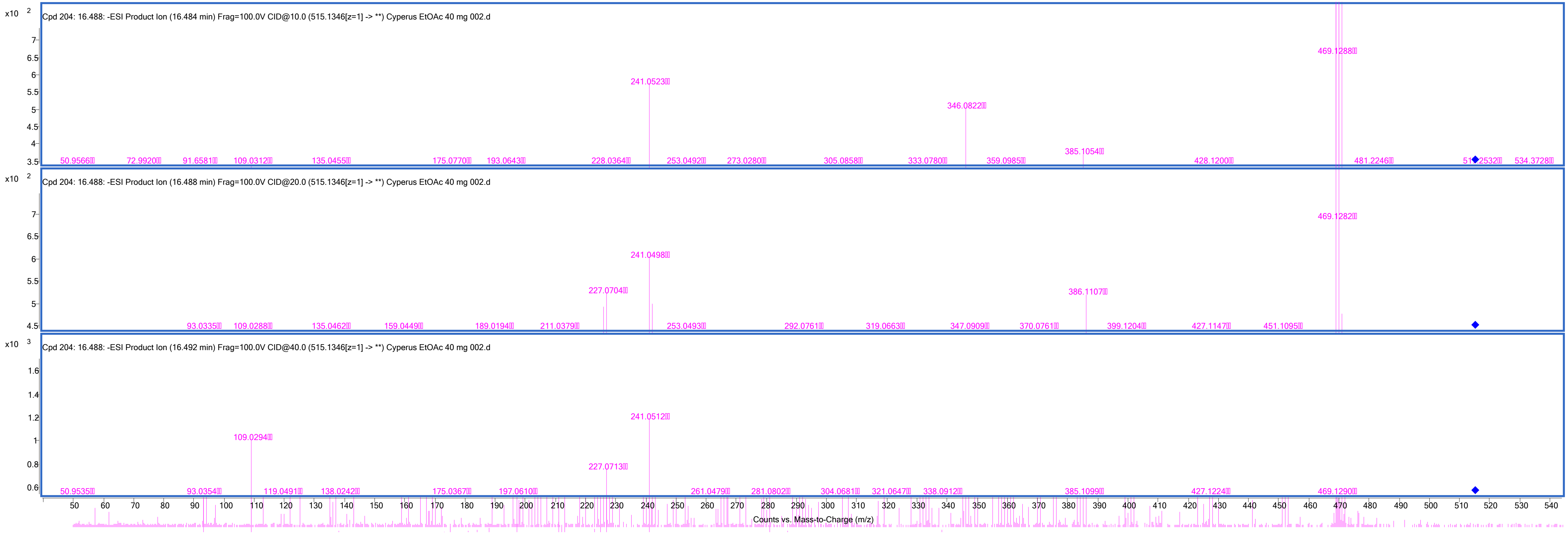

Figure S3A.29. The ESI-MS/MS fragmentation spectra of compound No 28 at m/z 469.1291 at various collision energies (10, 20, 40 eV) in the negative ionization mode.

Figure S3A.30. The purpose of fragmentation of scirpusin A

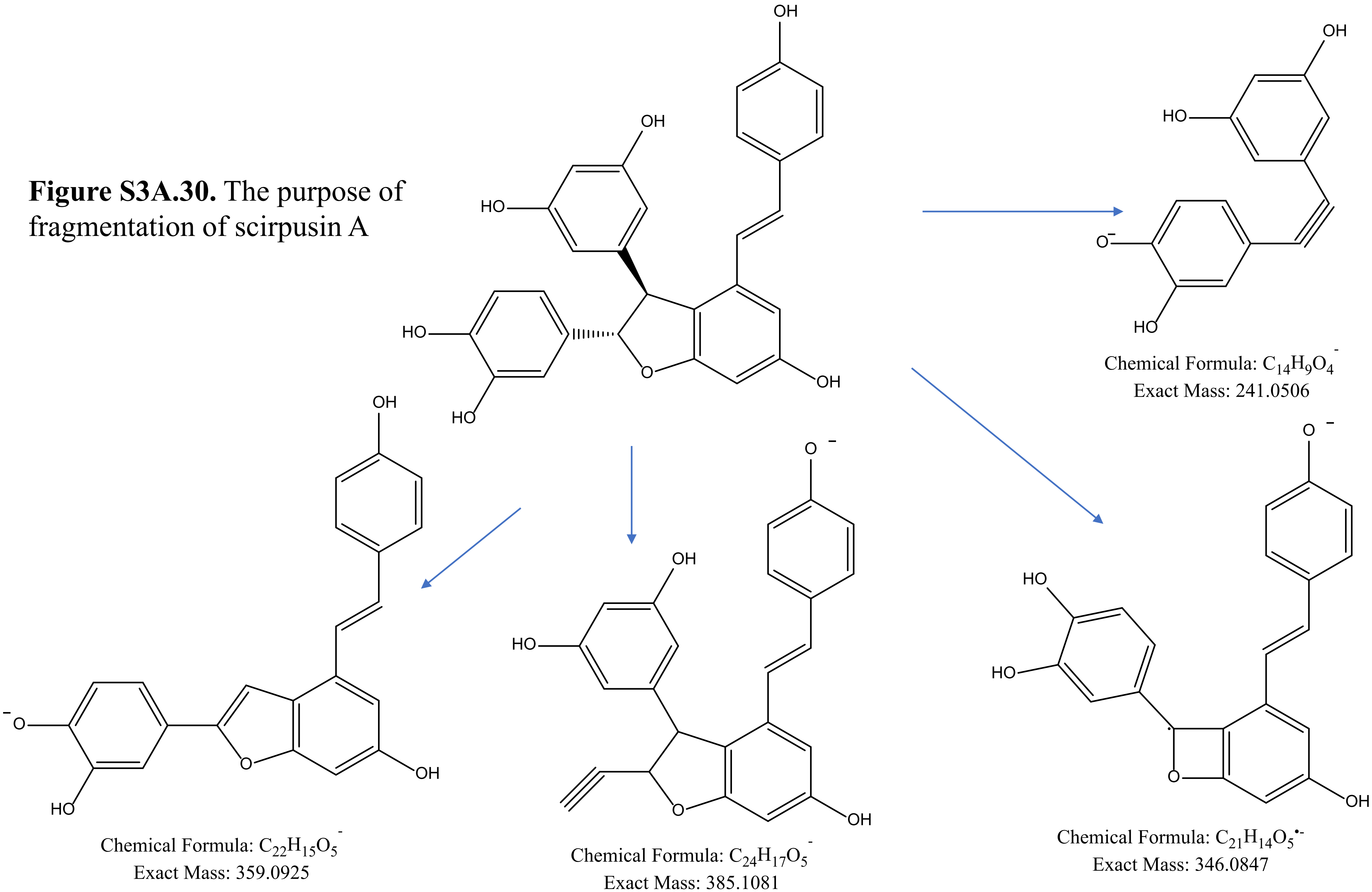

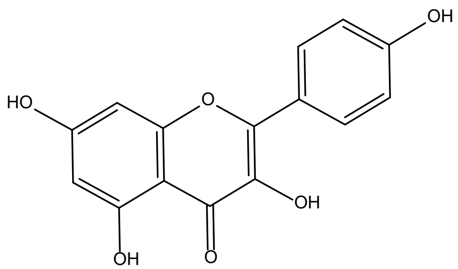

Kaempferol

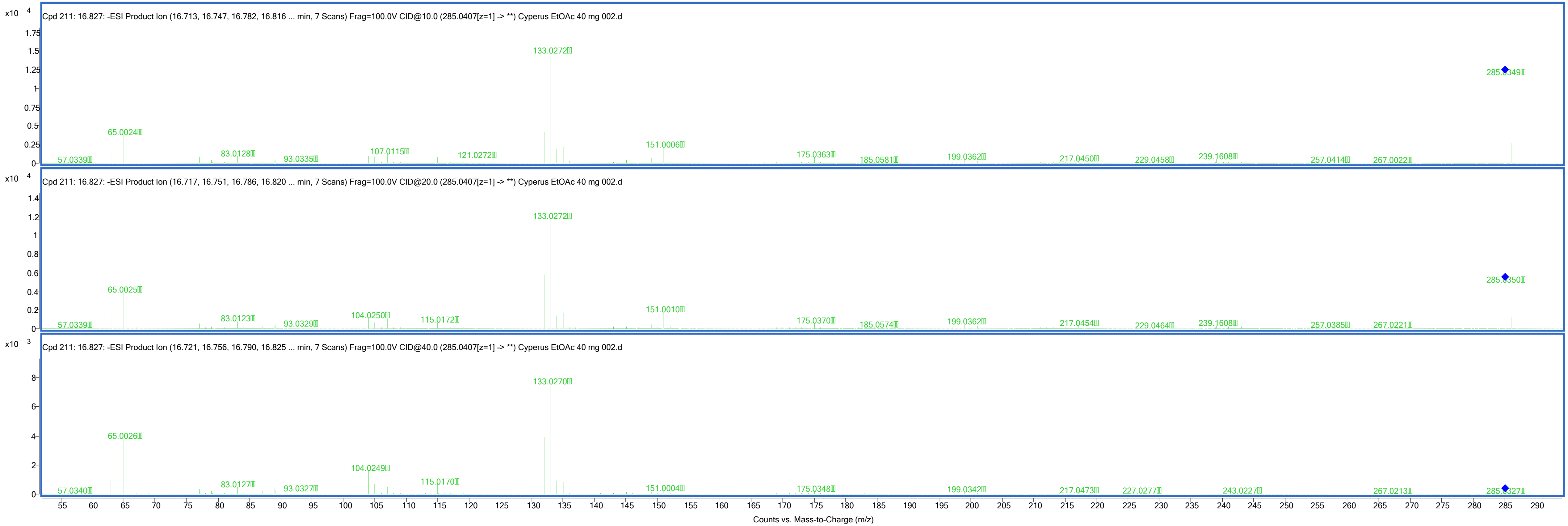

Figure S3A.31. The ESI-MS/MS fragmentation spectra of compound No 29 at m/z 285.0408 at various collision energies (10, 20, 40 eV) in the negative ionization mode.

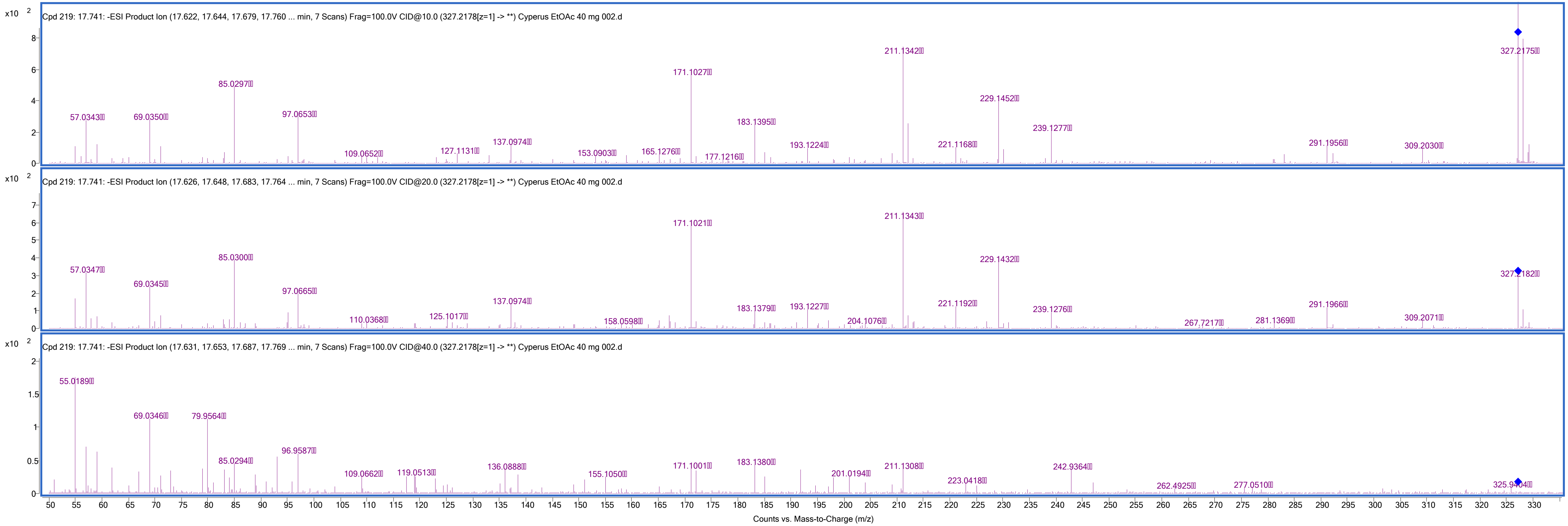

Figure S3A.32. The ESI-MS/MS fragmentation spectra of compound No 30 at m/z 327.2171 at various collision energies (10, 20, 40 eV) in the negative ionization mode.

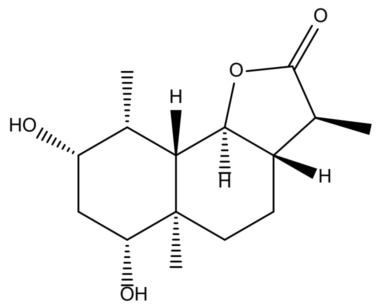

4,11,13,15-Tetrahydroridentin B

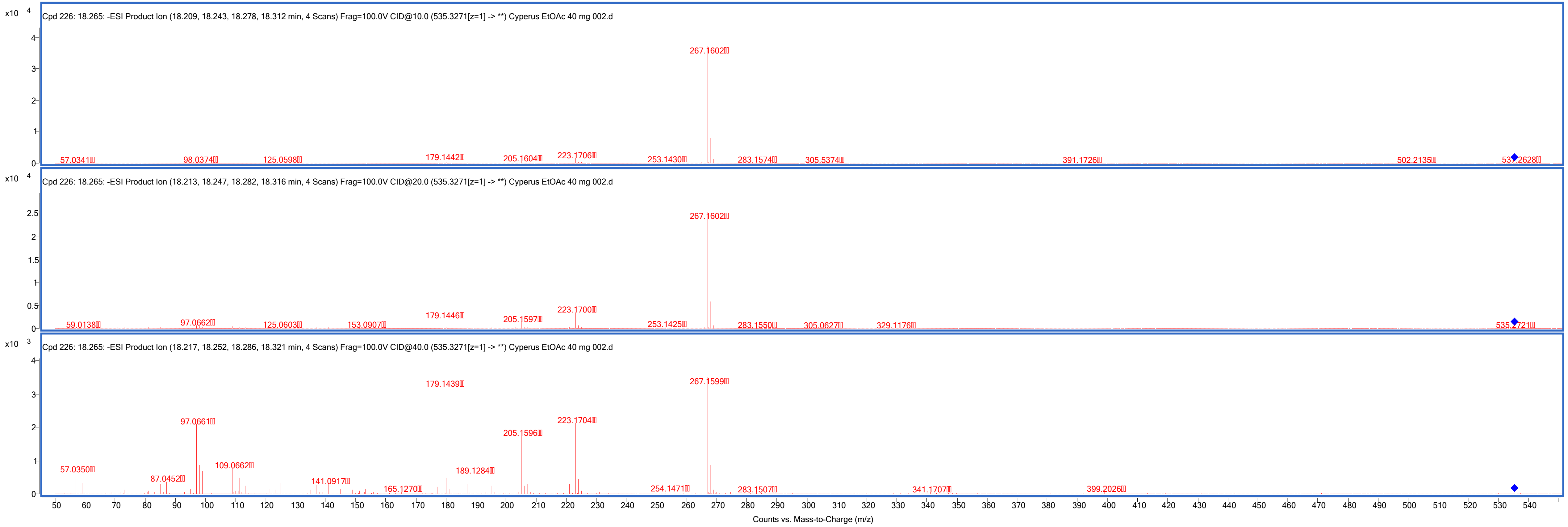

Figure S3A.33. The ESI-MS/MS fragmentation spectra of compound No 31 at m/z 267.1601 at various collision energies (10, 20, 40 eV) in the negative ionization mode.

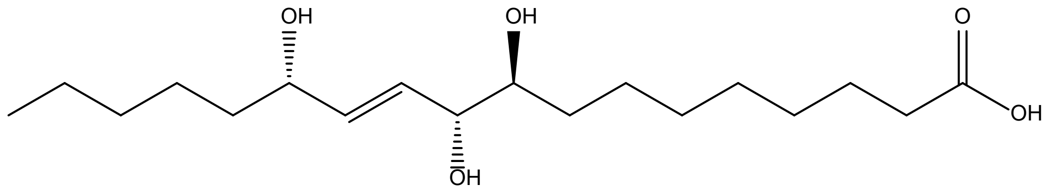

(9*S*,10*R*,11*E*,13*S*)-9,10,13-trihydroxyoctadec-11-enoic acid

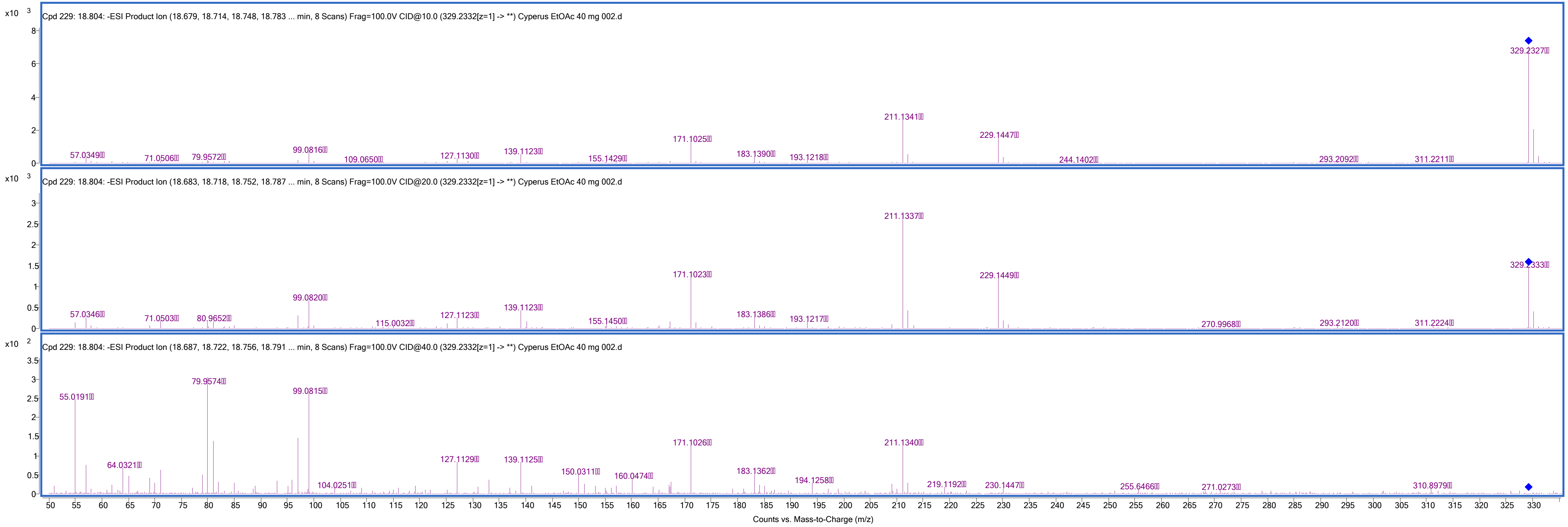

Figure S3A.34. The ESI-MS/MS fragmentation spectra of compound No 32 at m/z 329.2335 at various collision energies (10, 20, 40 eV) in the negative ionization mode.

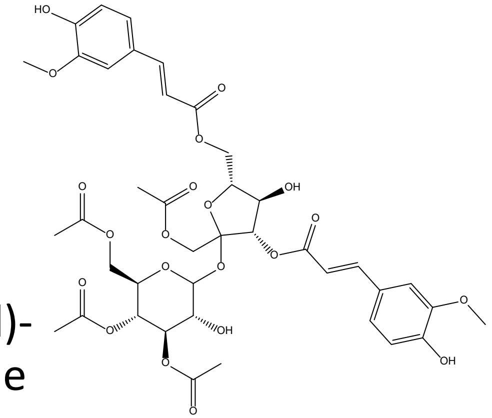

1-*O*-Acetyl-3-*O*,6-*O*-bis(3-methoxy-4-hydroxy-trans-cinnamoyl)-β-D-fructofuranosyl 3-*O*,4-*O*,6-*O*-triacetyl-α-D-glucopyranoside

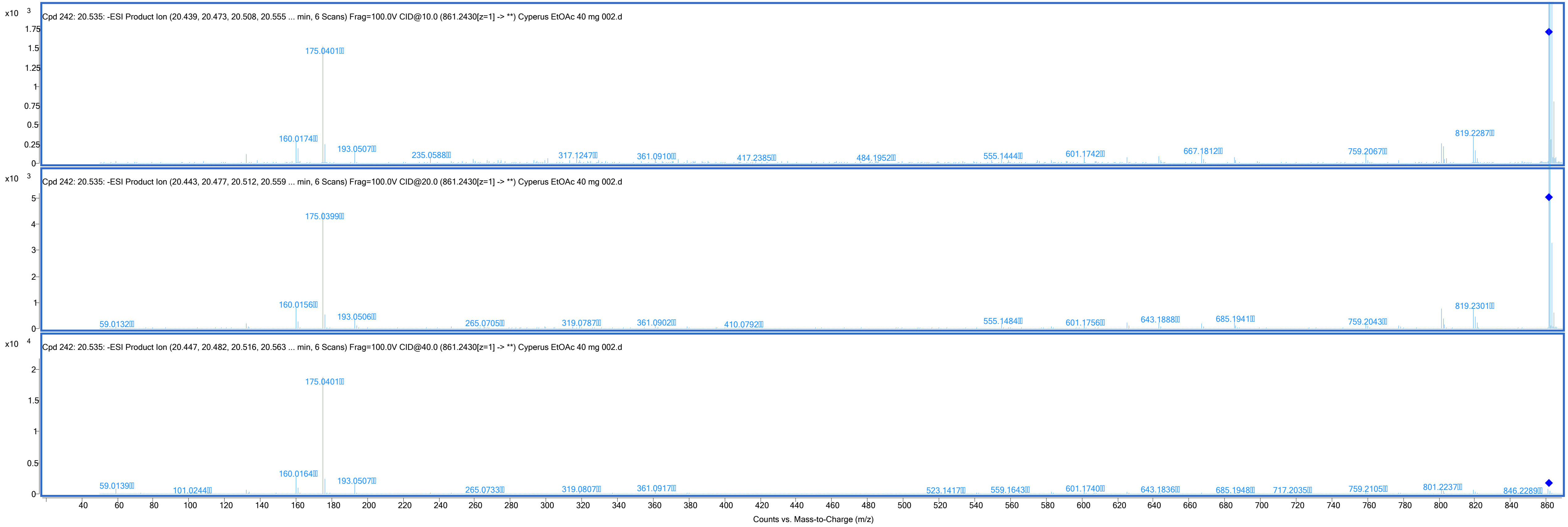

Figure S3A.35. The ESI-MS/MS fragmentation spectra of compound No 33 at m/z 861.2446 at various collision energies (10, 20, 40 eV) in the negative ionization mode.

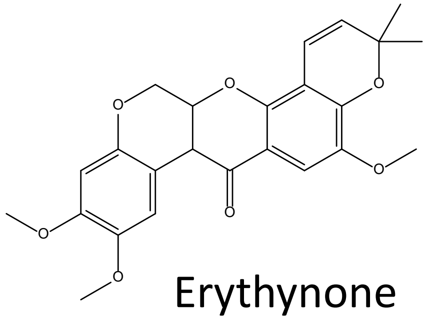

Erythynone

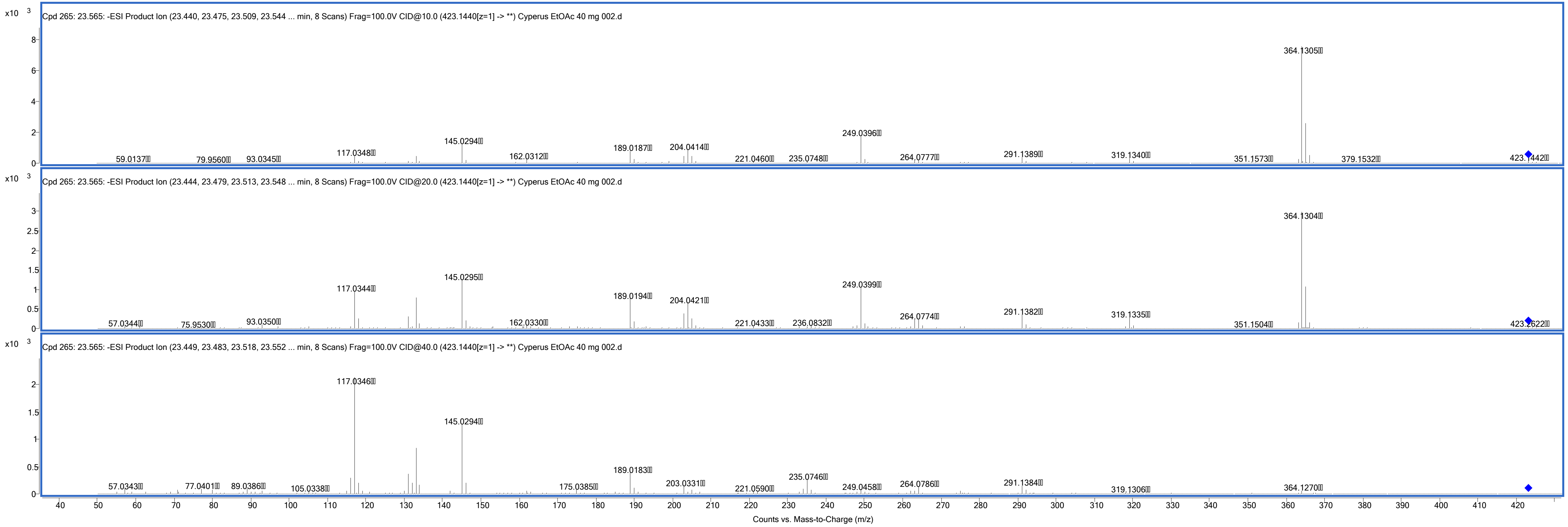

Figure S3A.36. The ESI-MS/MS fragmentation spectra of compound No 34 at m/z 423.1451 at various collision energies (10, 20, 40 eV) in the negative ionization mode.

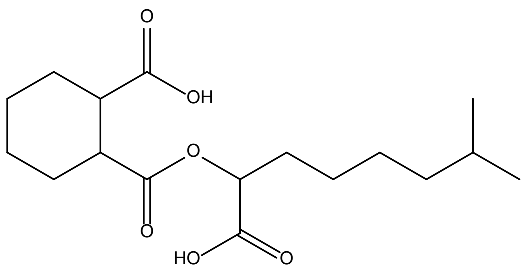

2-(((1-carboxy-6-methylheptyl)oxy)carbonyl)cyclohexane-1-carboxylic acid

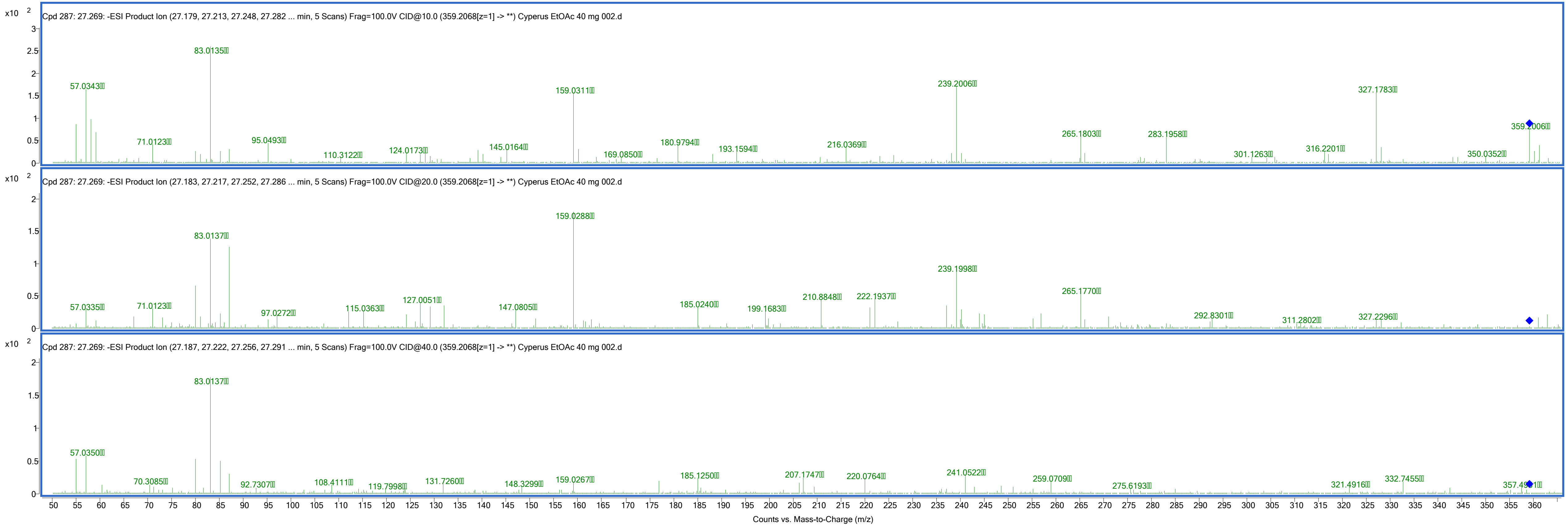

Figure S3A.37. The ESI-MS/MS fragmentation spectra of compound No 35 at m/z 327.1814 at various collision energies (10, 20, 40 eV) in the negative ionization mode.

MS/MS Fragmentation
Positive mode

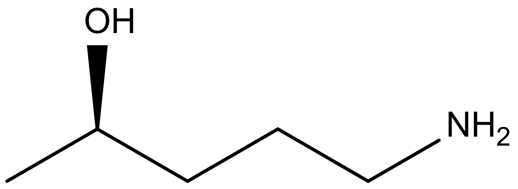

(*R*)-5-aminopentan-2-ol

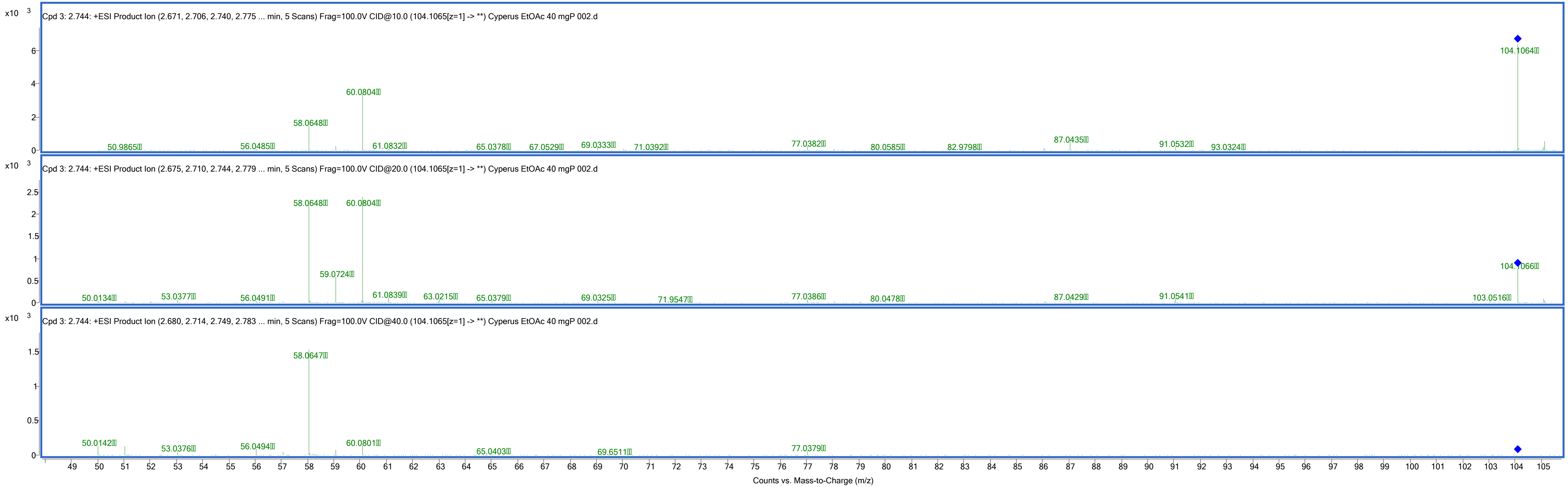

Figure S3B.1. The ESI-MS/MS fragmentation spectra of compound No 1 at m/z 104.1070 at various collision energies (10, 20, 40 eV) in the positive ionization mode.

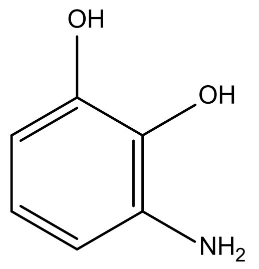

3-Aminobenzene-1,2-diol

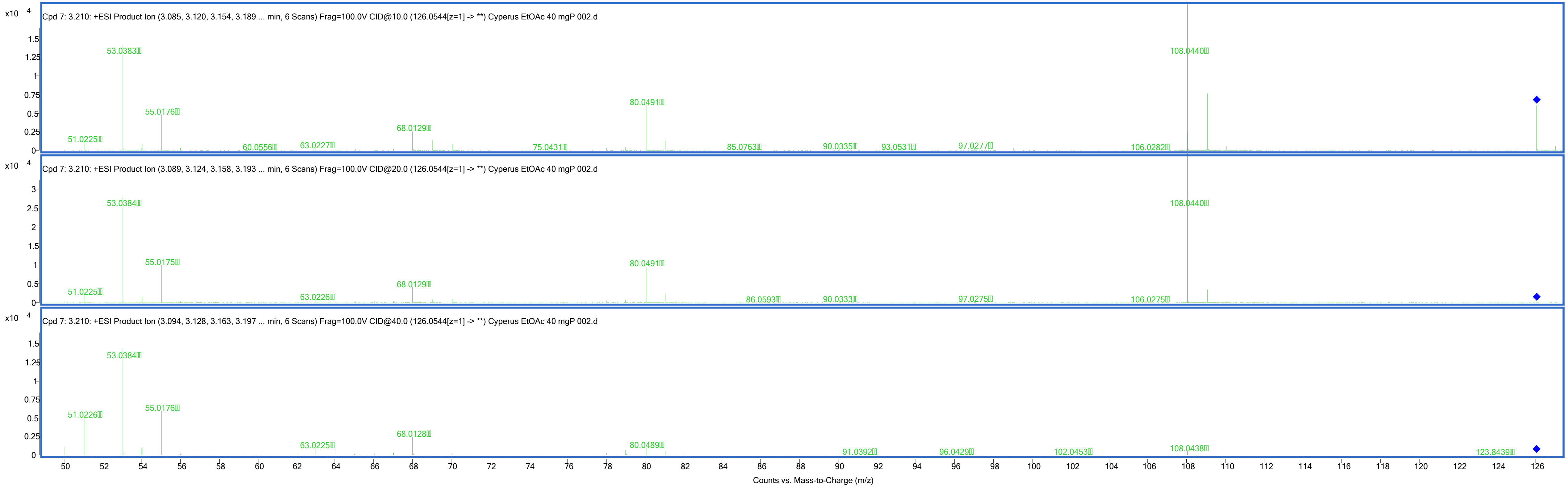

Figure S3B.2. The ESI-MS/MS fragmentation spectra of compound No 2 at m/z 126.0549 at various collision energies (10, 20, 40 eV) in the positive ionization mode.

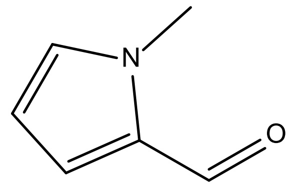

1-Methyl-2-pyrrolecarboxaldehyde

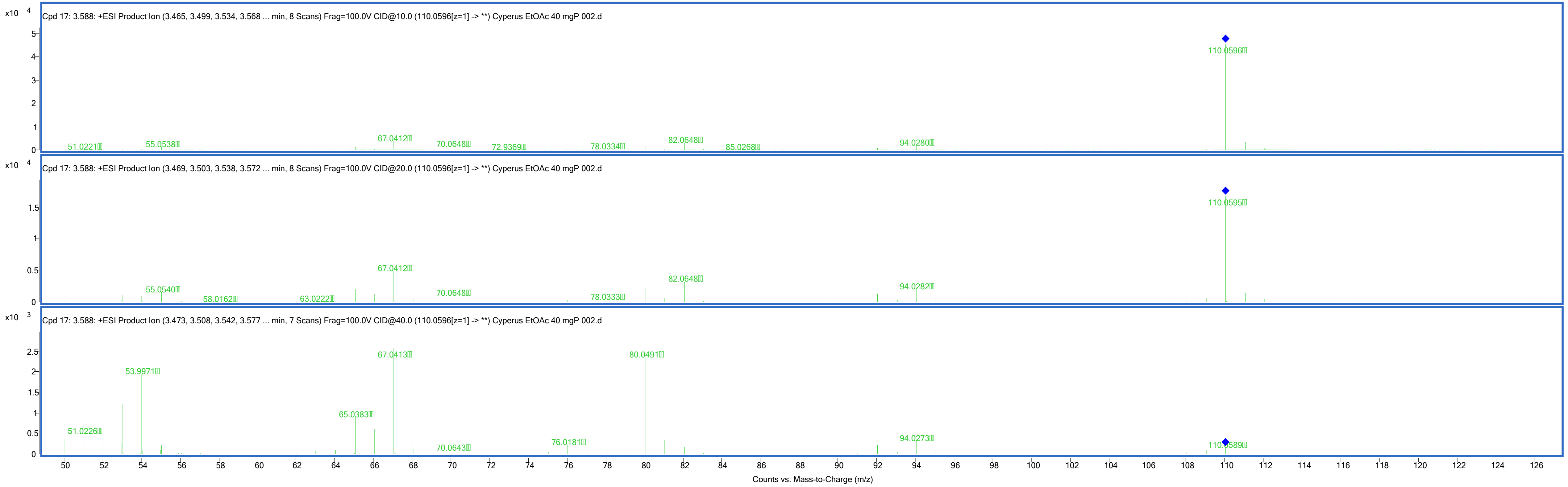

Figure S3B.3. The ESI-MS/MS fragmentation spectra of compound No 3 at m/z 110.0602 at various collision energies (10, 20, 40 eV) in the positive ionization mode.

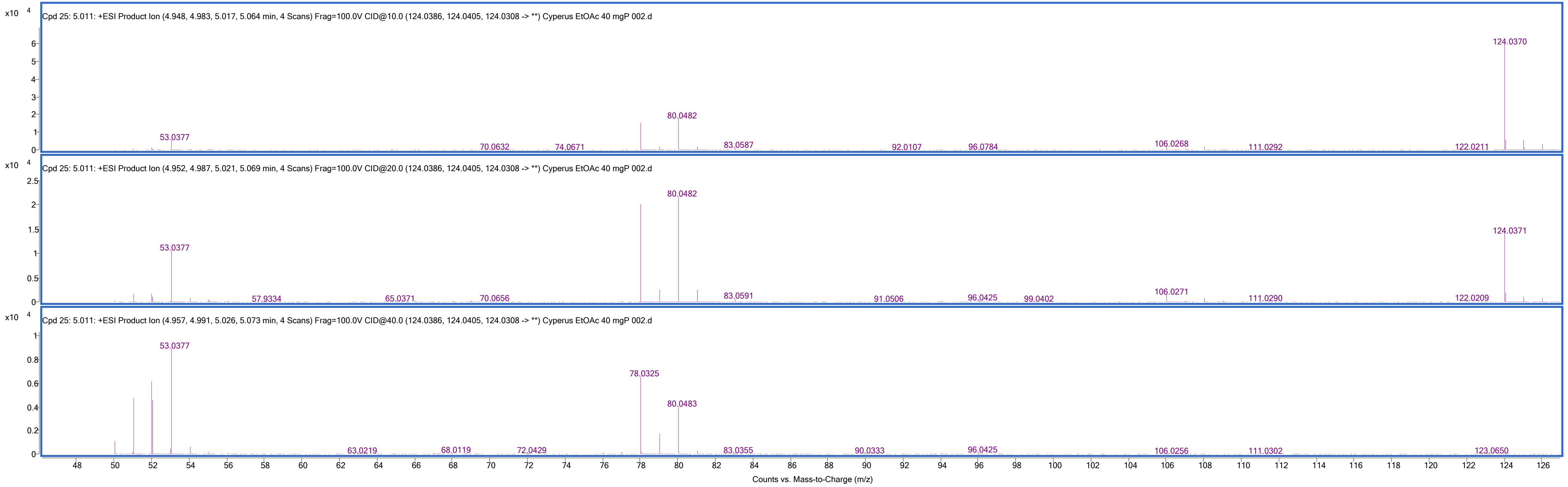

Figure S3B.4. The ESI-MS/MS fragmentation spectra of compound No 4 at m/z 124.0427 at various collision energies (10, 20, 40 eV) in the positive ionization mode.

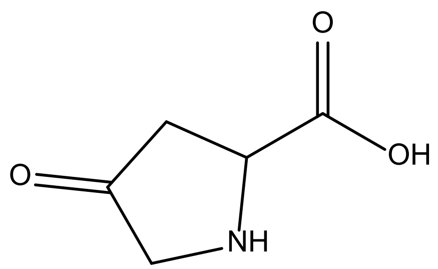

4-oxo-L-proline

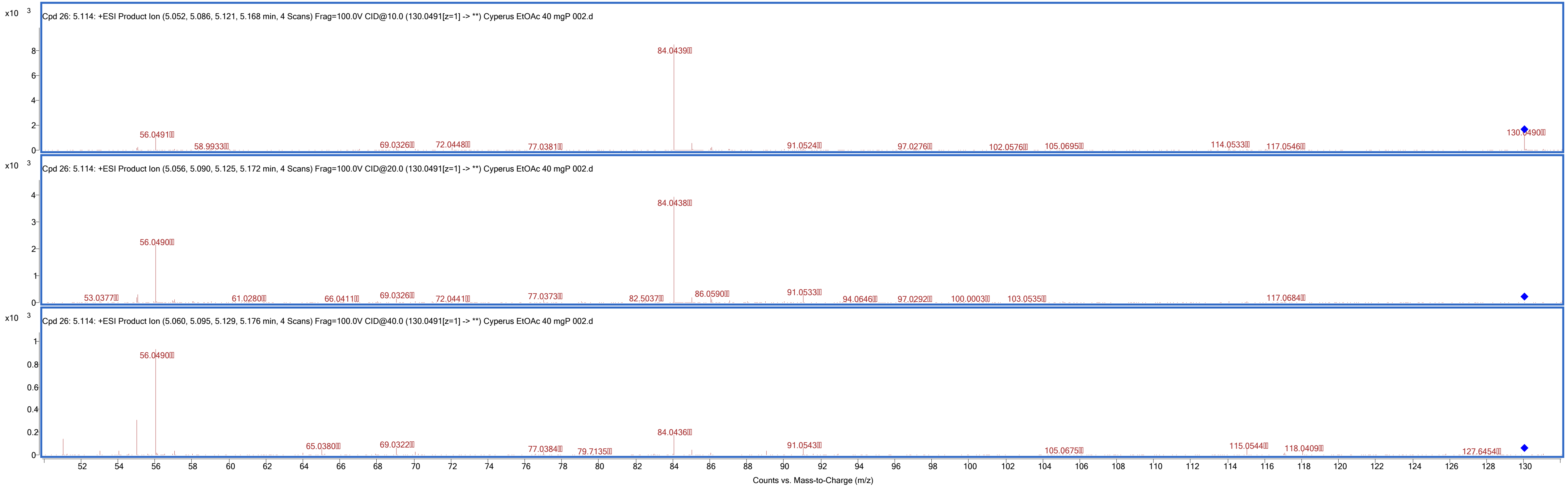

Figure S3B.5. The ESI-MS/MS fragmentation spectra of compound No 5 at m/z 130.0496 at various collision energies (10, 20, 40 eV) in the positive ionization mode.

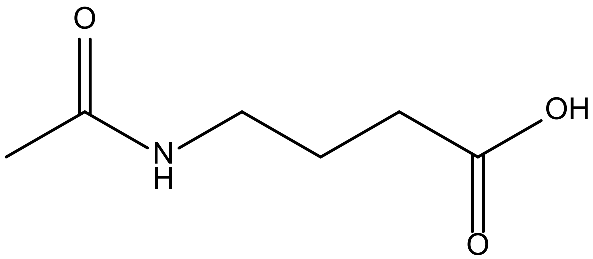

4-Acetamidobutanoic acid

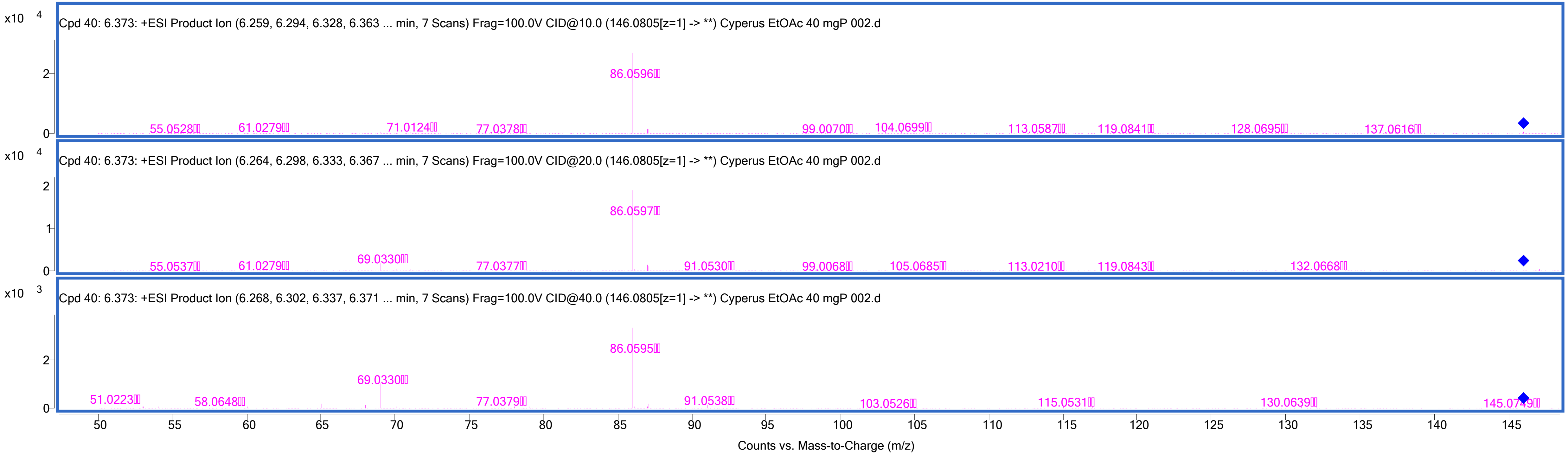

Figure S3B.6. The ESI-MS/MS fragmentation spectra of compound No 6 at m/z 146.0814 at various collision energies (10, 20, 40 eV) in the positive ionization mode.

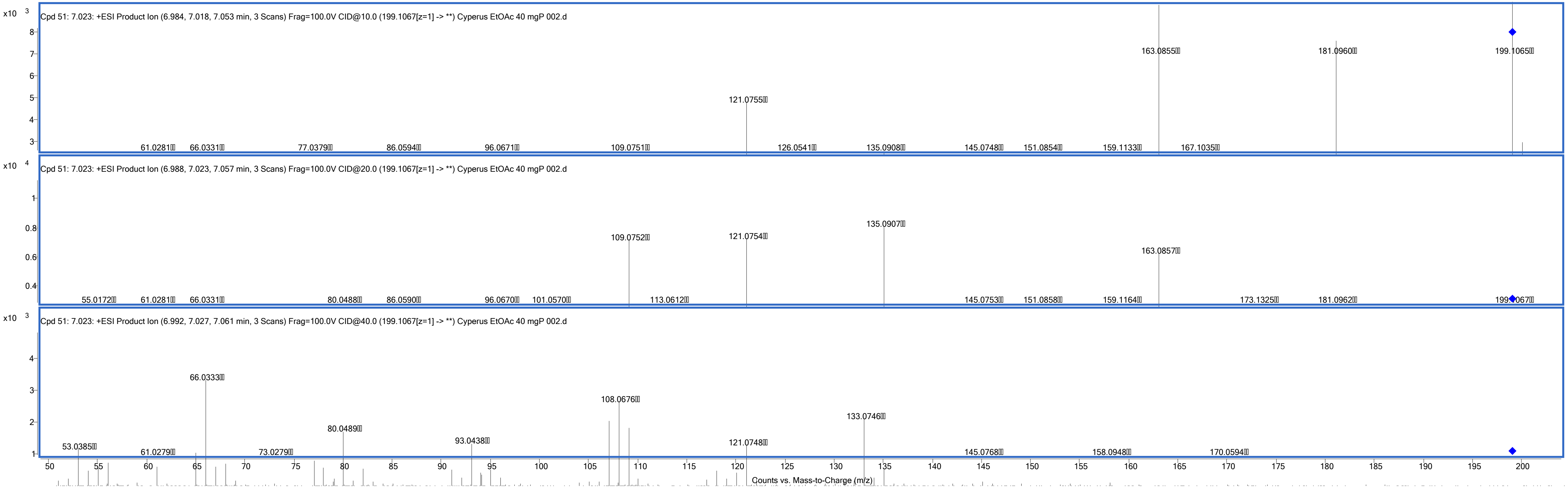

Figure S3B.7. The ESI-MS/MS fragmentation spectra of compound No 7 at m/z 199.1078 at various collision energies (10, 20, 40 eV) in the positive ionization mode.

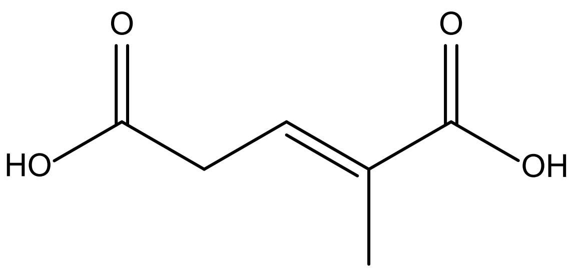

(*E*)-2-Methylglutaconic acid

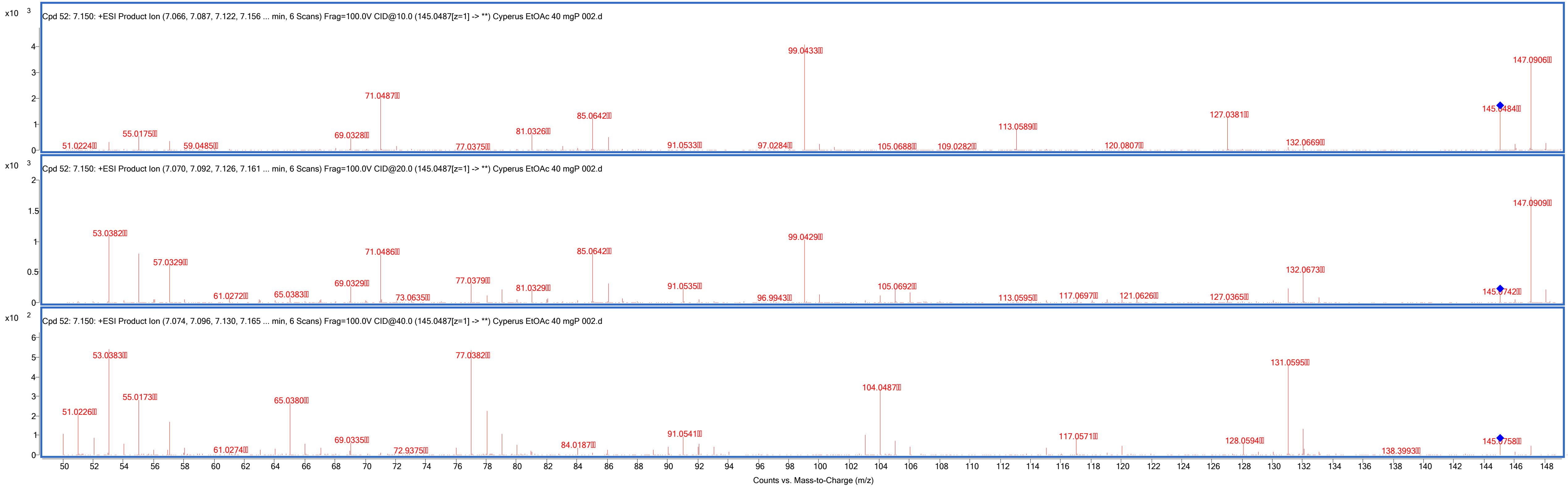

Figure S3B.8. The ESI-MS/MS fragmentation spectra of compound No 8 at m/z 145.0498 at various collision energies (10, 20, 40 eV) in the positive ionization mode.

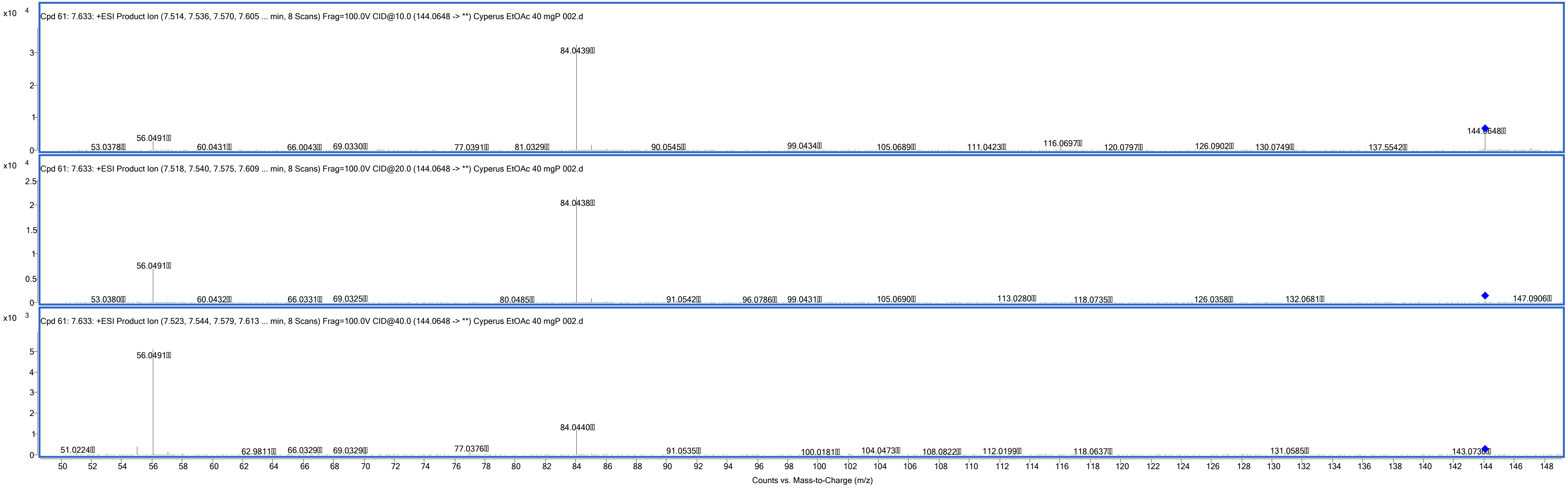

Figure S3B.9. The ESI-MS/MS fragmentation spectra of compound No 9 at m/z 144.0655 at various collision energies (10, 20, 40 eV) in the positive ionization mode.

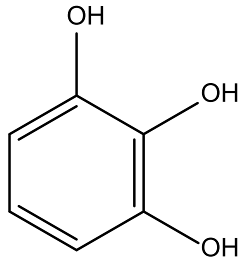

1,2,3-Trihydroxybenzene

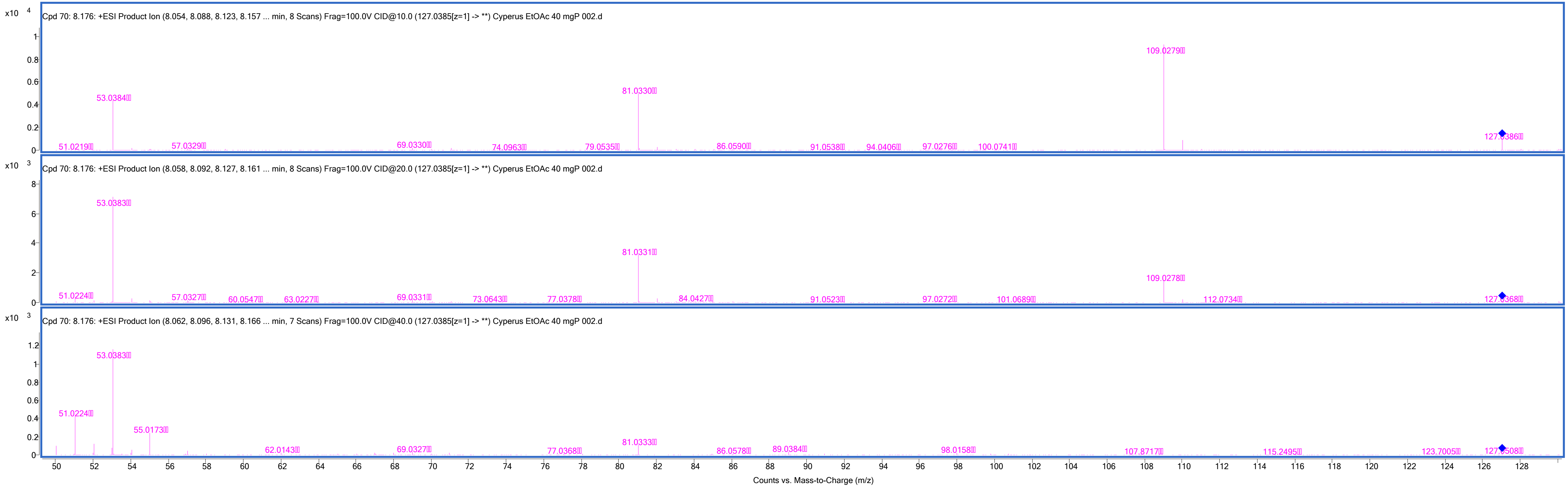

Figure S3B.10. The ESI-MS/MS fragmentation spectra of compound No 10 at m/z 127.0394 at various collision energies (10, 20, 40 eV) in the positive ionization mode.

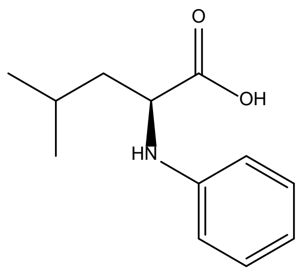

Phenyl-Leucine

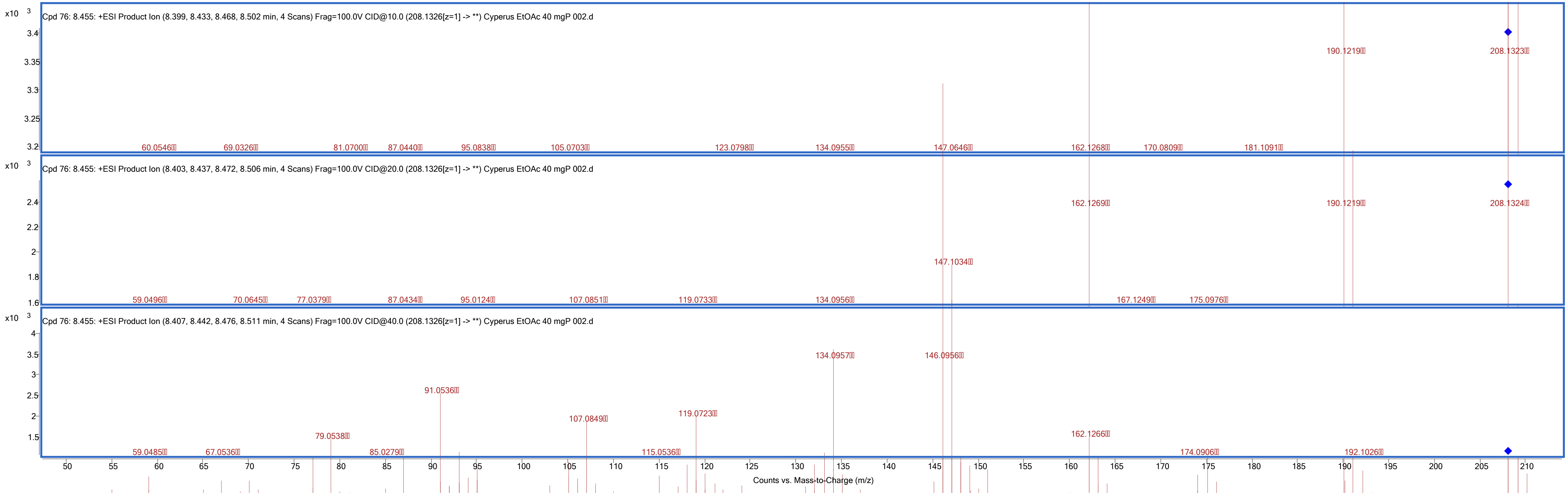

Figure S3B.11. The ESI-MS/MS fragmentation spectra of compound No 11 at m/z 208.1333 at various collision energies (10, 20, 40 eV) in the positive ionization mode.

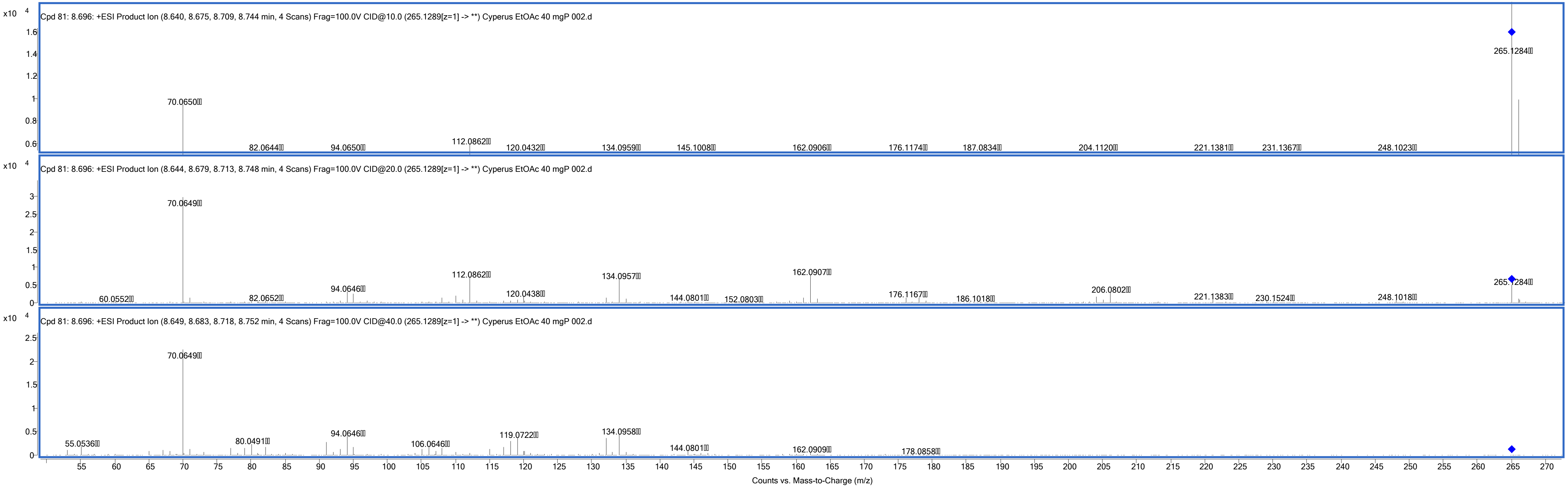

Figure S3B.12. The ESI-MS/MS fragmentation spectra of compound No 12 at m/z 265.1295 at various collision energies (10, 20, 40 eV) in the positive ionization mode.

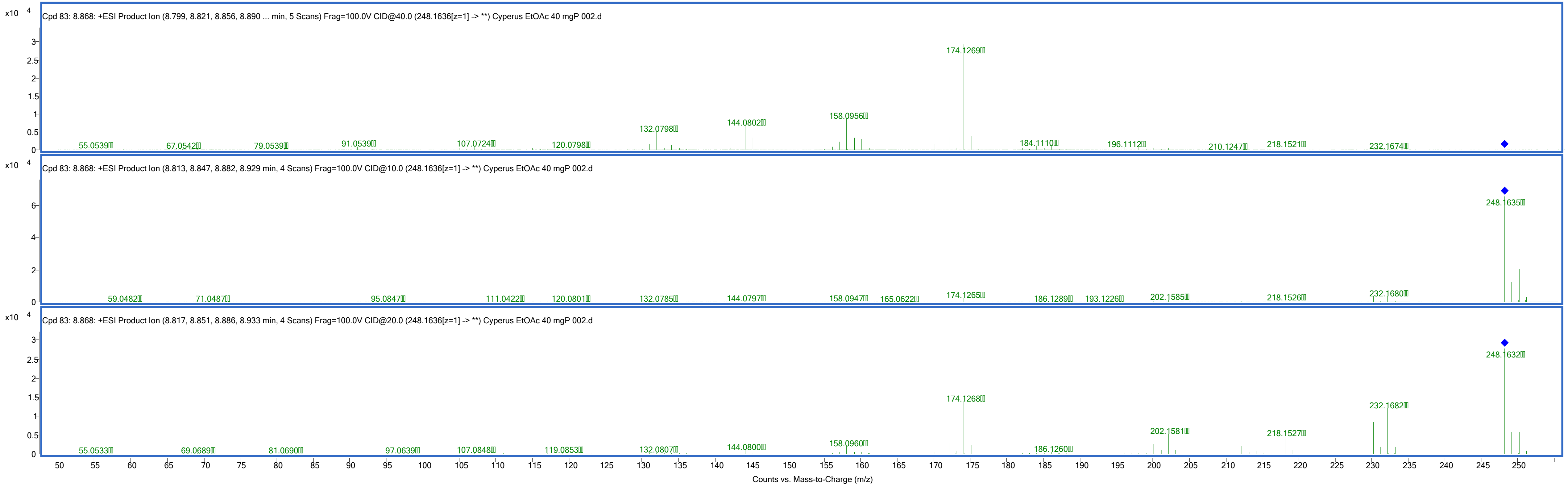

Figure S3B.13. The ESI-MS/MS fragmentation spectra of compound No 13 at m/z 248.1645 at various collision energies (10, 20, 40 eV) in the positive ionization mode.

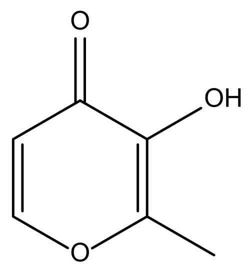

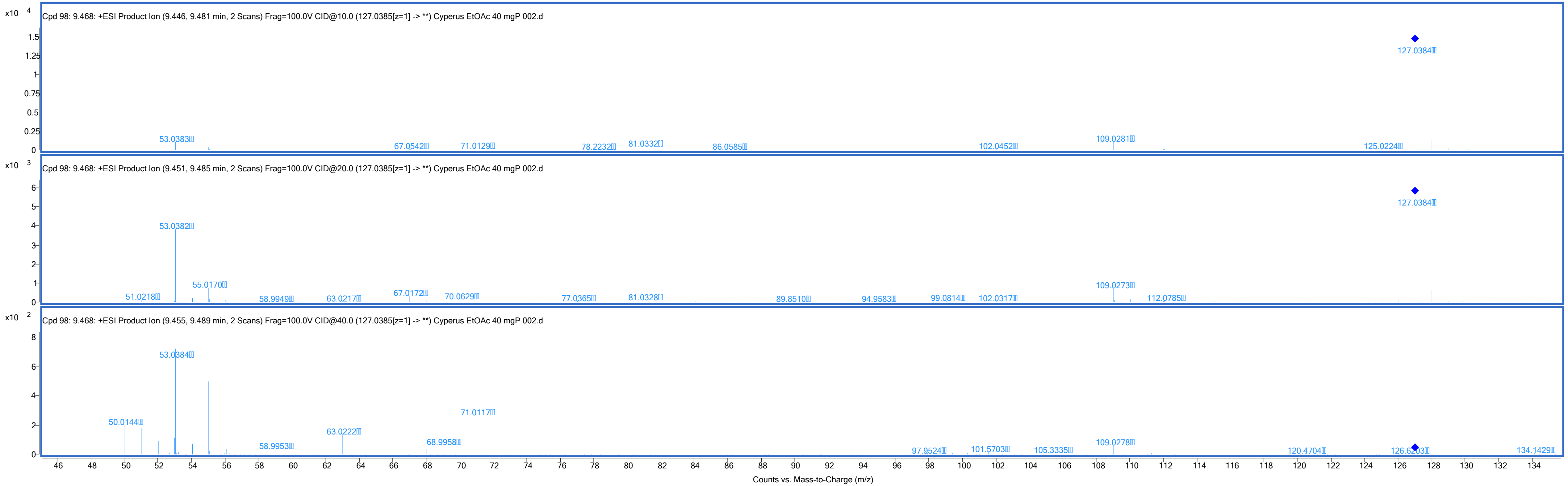

Figure S3B.14. The ESI-MS/MS fragmentation spectra of compound No 14 at m/z 127.0391 at various collision energies (10, 20, 40 eV) in the positive ionization mode.

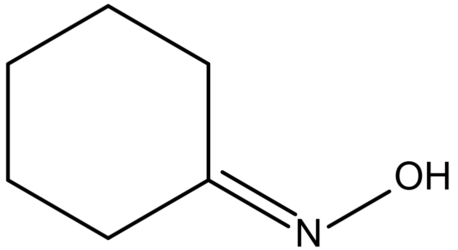

Cyclohexanone oxime

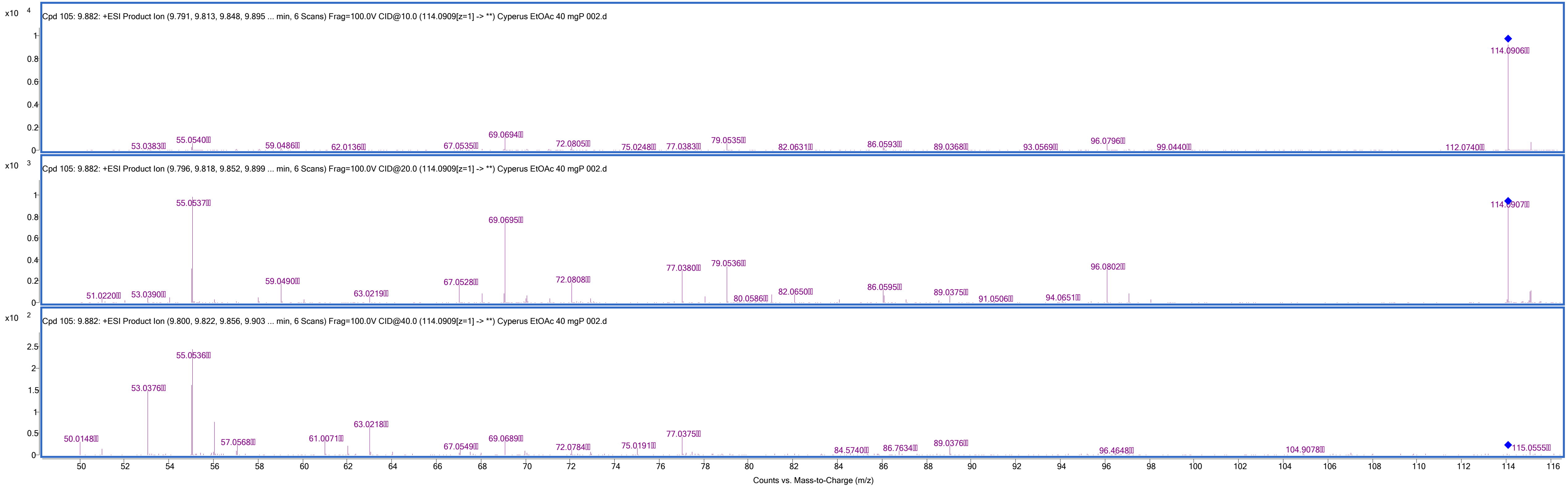

Figure S3B.15. The ESI-MS/MS fragmentation spectra of compound No 15 at m/z 114.0914 at various collision energies (10, 20, 40 eV) in the positive ionization mode.

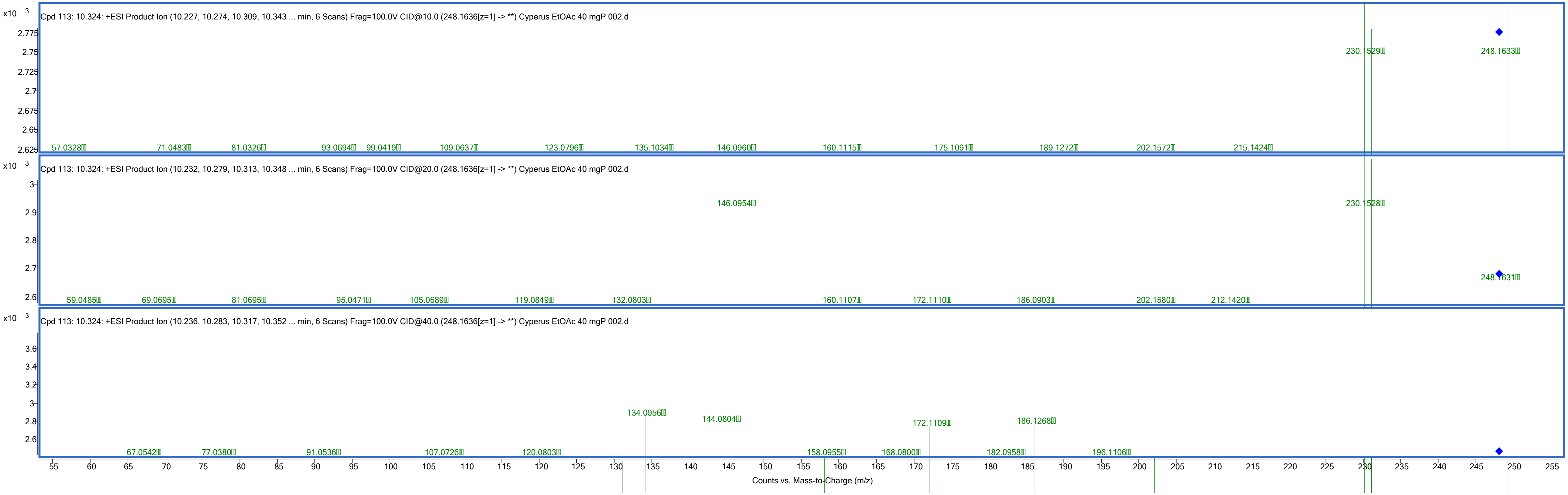

Figure S3B.16. The ESI-MS/MS fragmentation spectra of compound No 16 at m/z 248.1646 at various collision energies (10, 20, 40 eV) in the positive ionization mode.

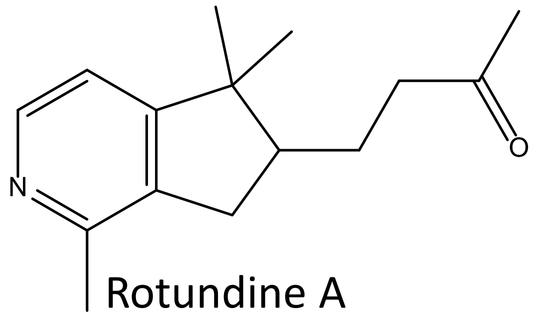

Rotundine A

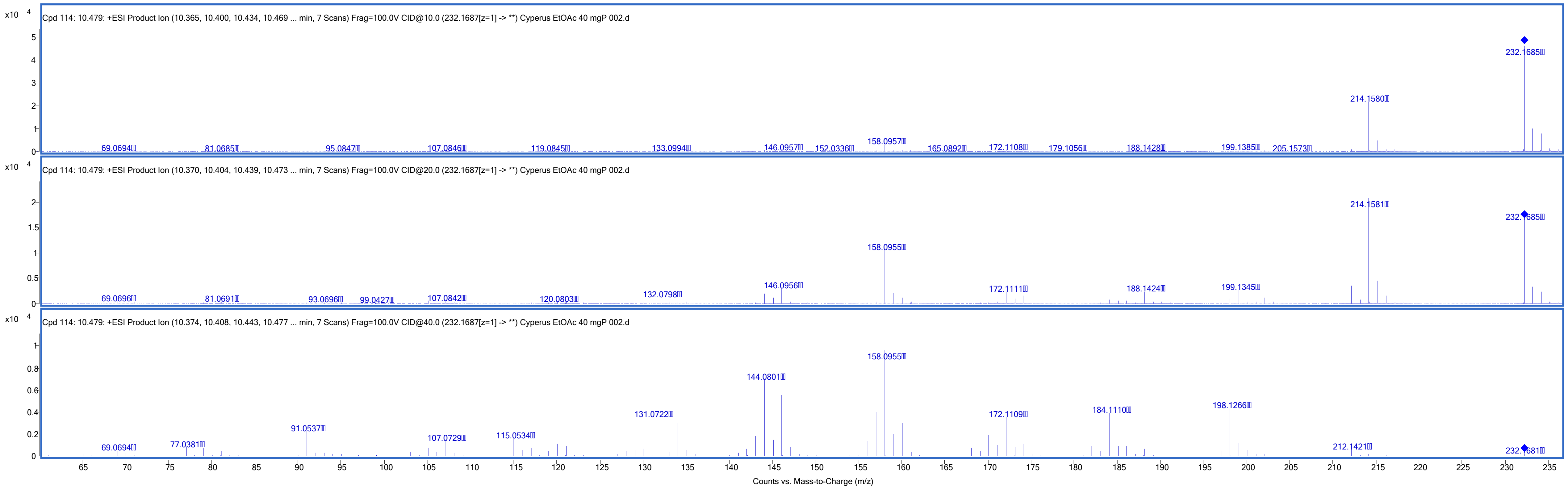

Figure S3B.17. The ESI-MS/MS fragmentation spectra of compound No 17 at m/z 232.1695 at various collision energies (10, 20, 40 eV) in the positive ionization mode.

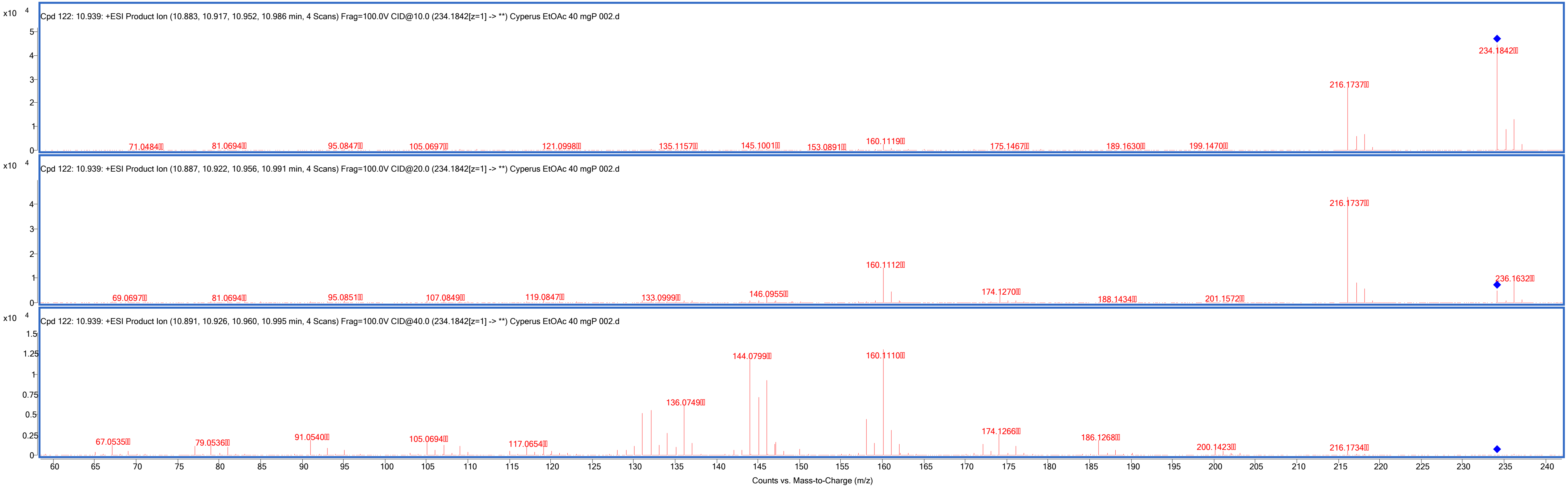

Figure S3B.18. The ESI-MS/MS fragmentation spectra of compound No 18 at m/z 234.1852 at various collision energies (10, 20, 40 eV) in the positive ionization mode.

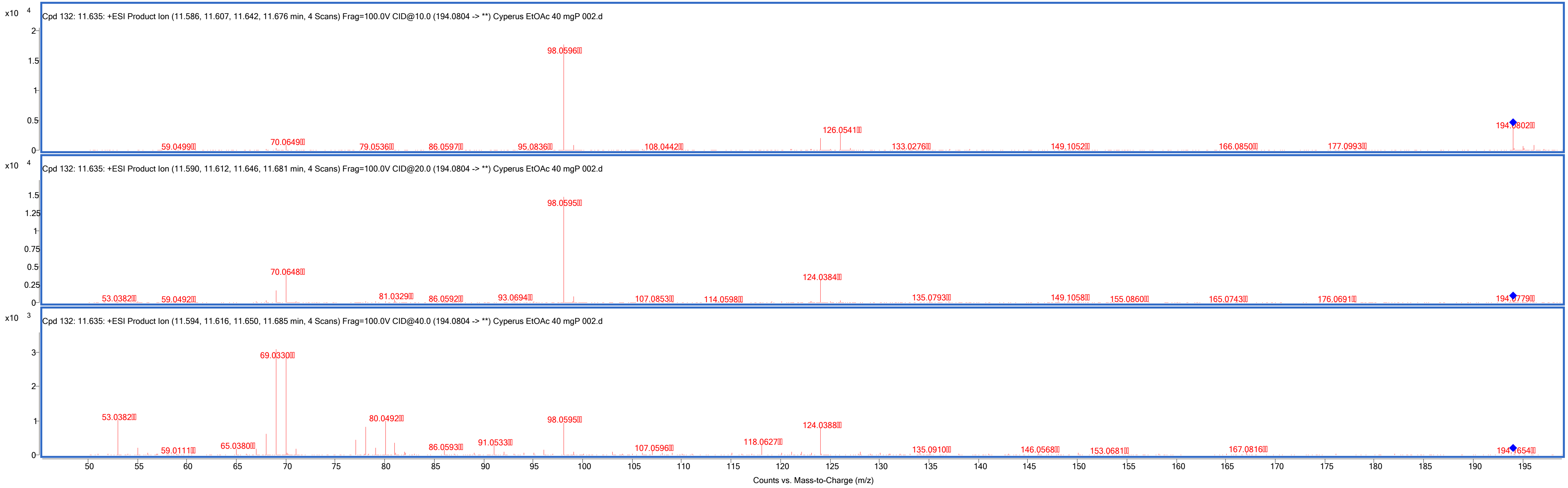

Figure S3B.19. The ESI-MS/MS fragmentation spectra of compound No 19 at m/z 194.0812 at various collision energies (10, 20, 40 eV) in the positive ionization mode.

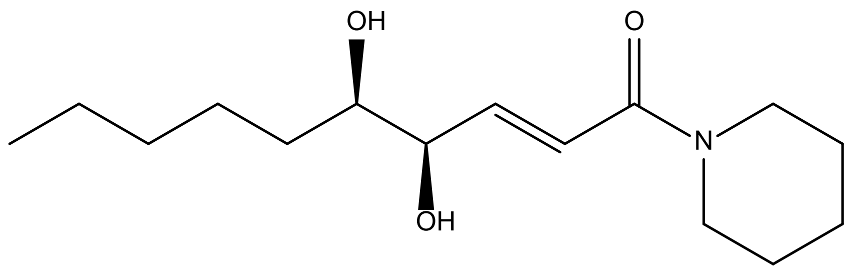

(2*E*,4*R*,5*R*)-4,5-Dihydroxy-1-(1-piperidinyl)-2-decen-1-one

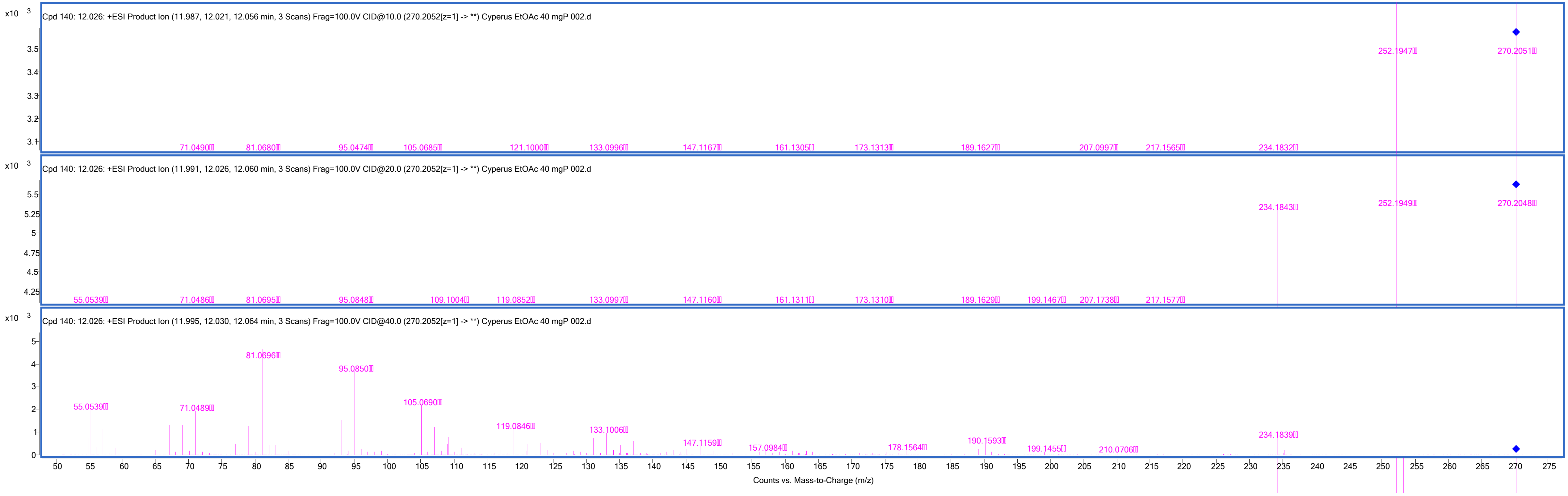

Figure S3B.20. The ESI-MS/MS fragmentation spectra of compound No 20 at m/z 270.2062 at various collision energies (10, 20, 40 eV) in the positive ionization mode.

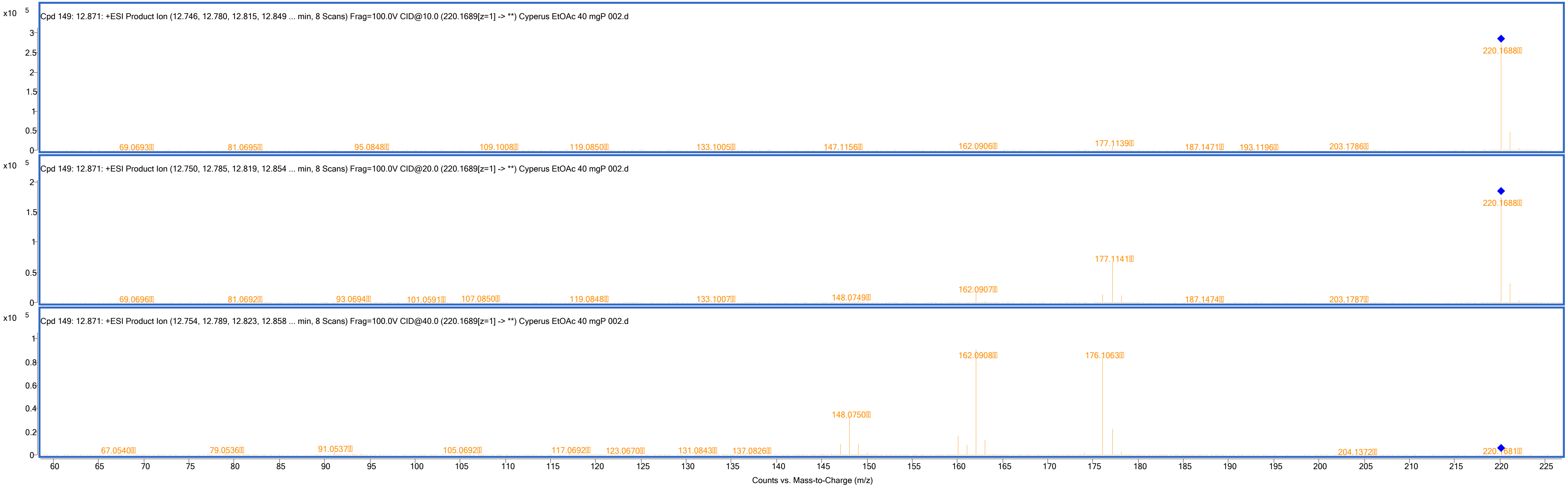

Figure S3B.21. The ESI-MS/MS fragmentation spectra of compound No 21 at m/z 220.1694 at various collision energies (10, 20, 40 eV) in the positive ionization mode.

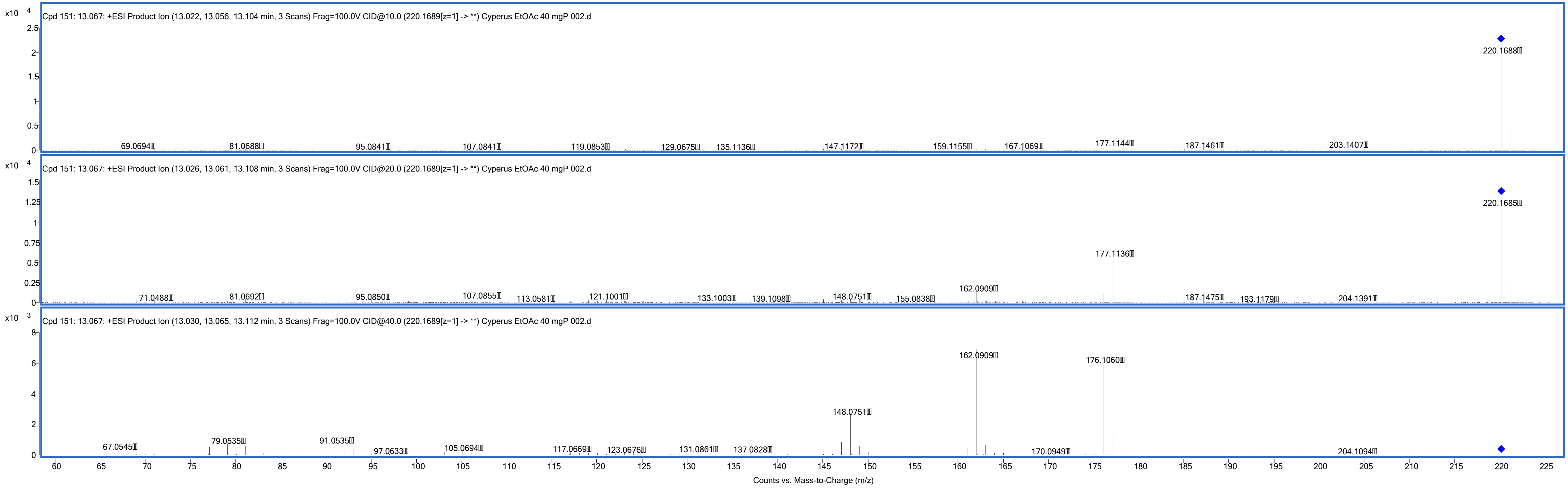

Figure S3B.22. The ESI-MS/MS fragmentation spectra of compound No 22 at m/z 220.1694 at various collision energies (10, 20, 40 eV) in the positive ionization mode.

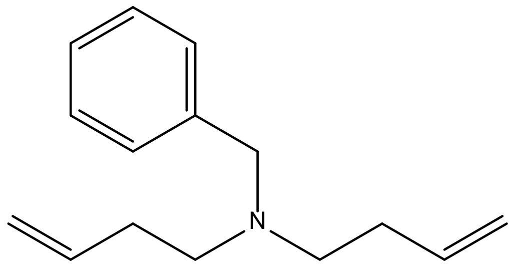

N-benzyl-N-but-3-enylbut-3-en-1-amine

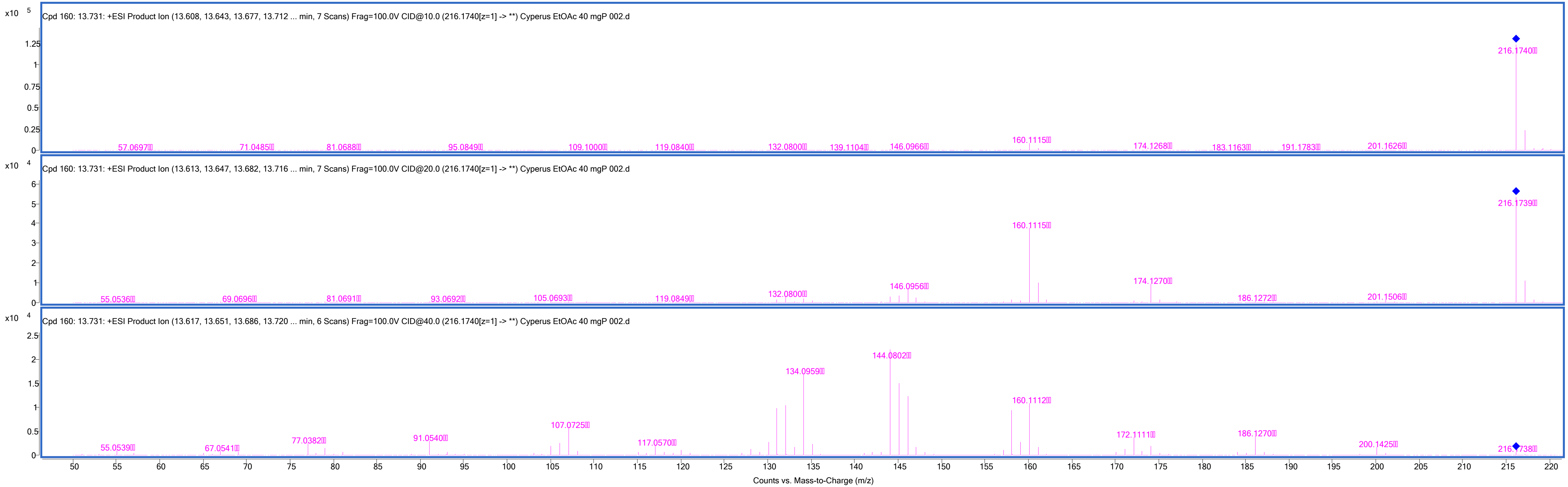

Figure S3B.23. The ESI-MS/MS fragmentation spectra of compound No 23 at m/z 216.1744 at various collision energies (10, 20, 40 eV) in the positive ionization mode.

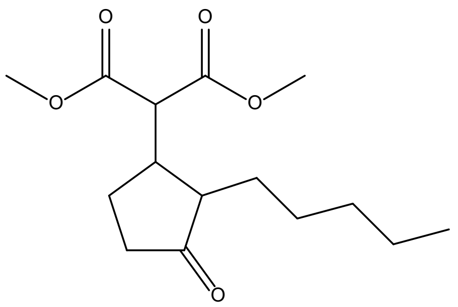

Dimethyl (3-oxo-2-pentylcyclopentyl)malonate

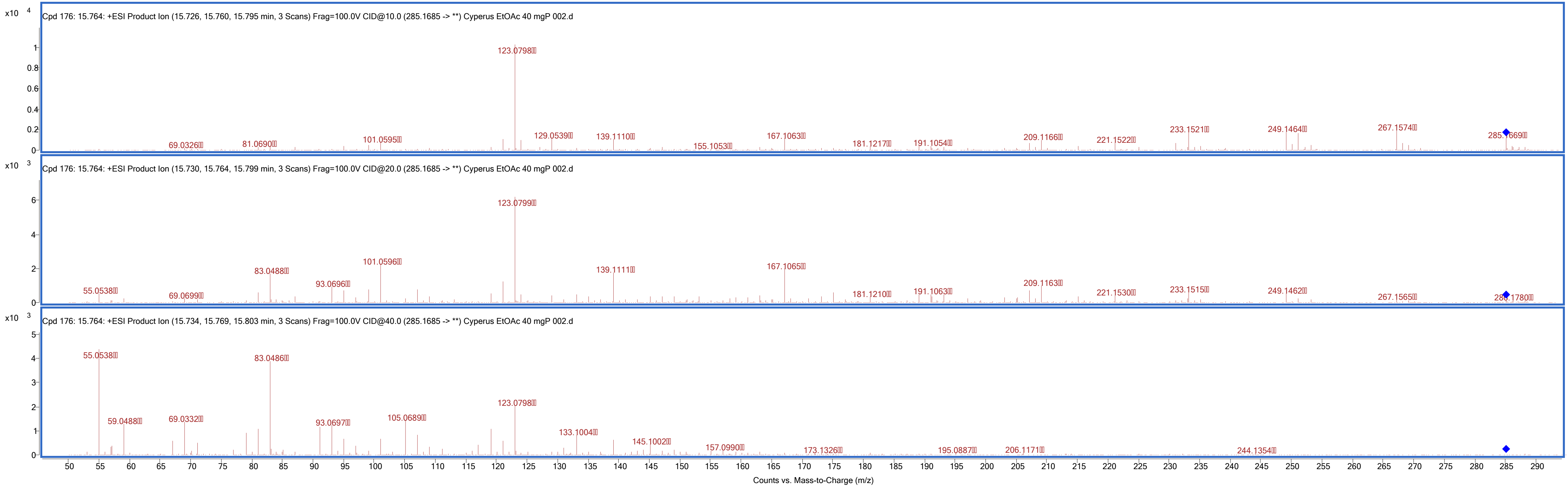

Figure S3B.24. The ESI-MS/MS fragmentation spectra of compound No 23 at m/z 216.1744 at various collision energies (10, 20, 40 eV) in the positive ionization mode.

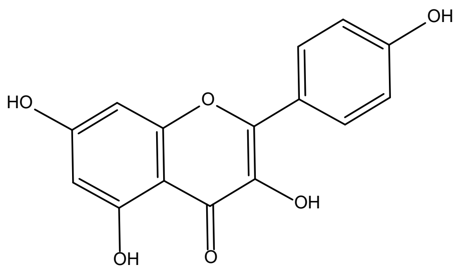

Kaempferol

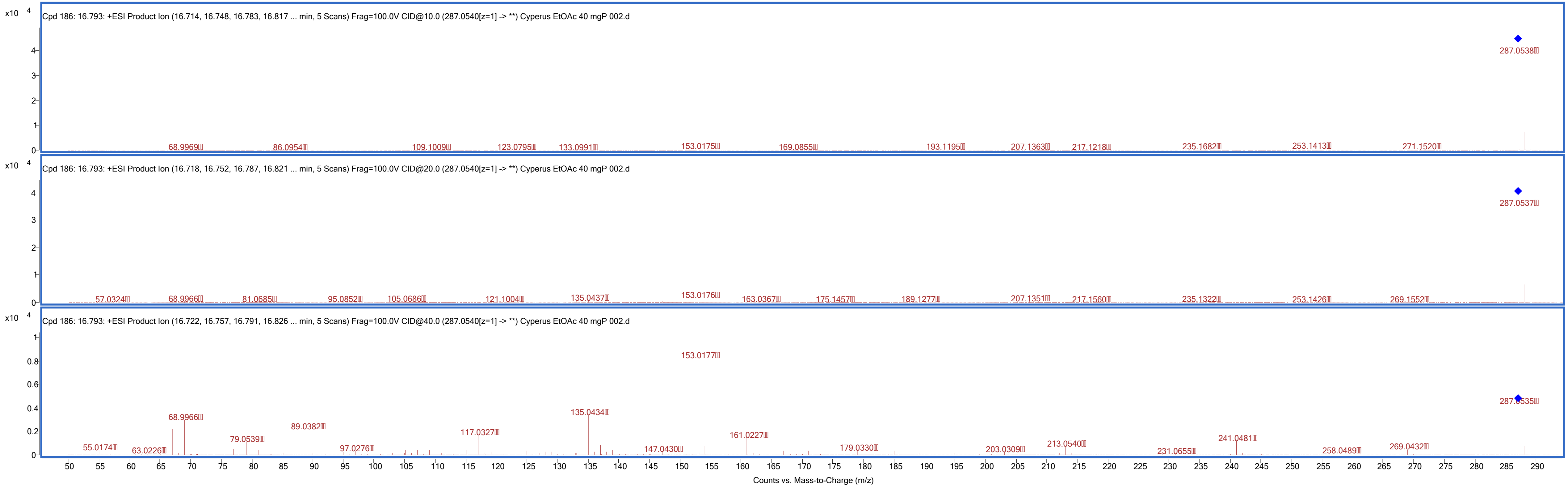

Figure S3B.25. The ESI-MS/MS fragmentation spectra of compound No 25 at m/z 287.0548 at various collision energies (10, 20, 40 eV) in the positive ionization mode.

Camphor

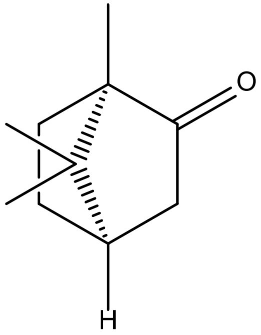

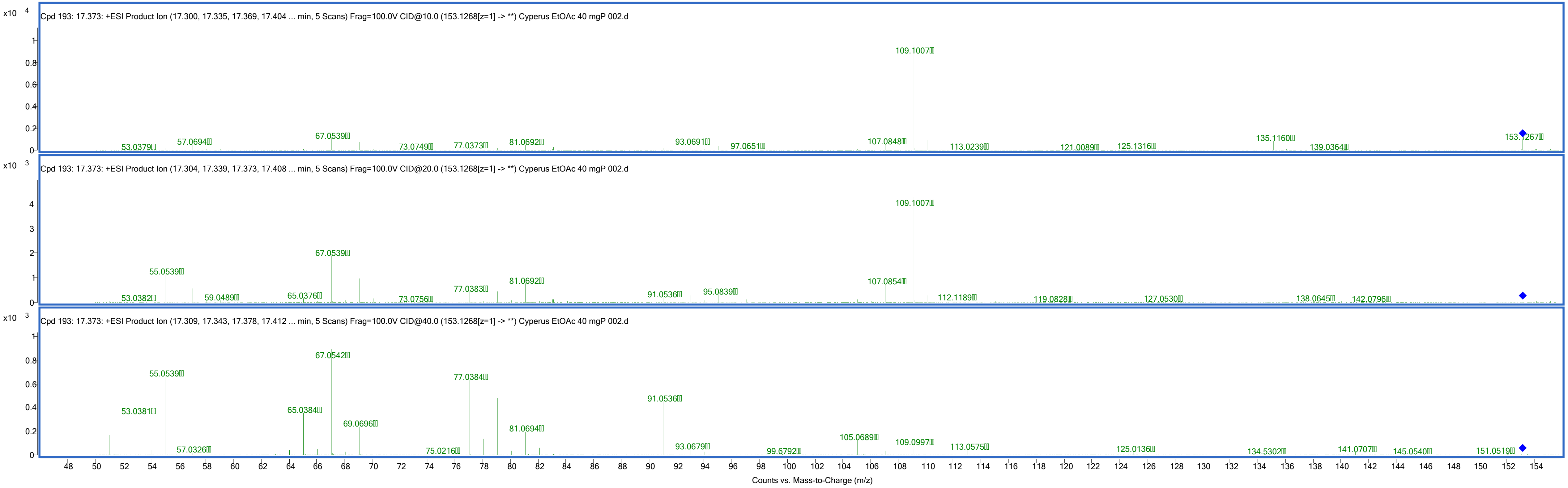

Figure S3B.26. The ESI-MS/MS fragmentation spectra of compound No 26 at m/z 153.1272 at various collision energies (10, 20, 40 eV) in the positive ionization mode.

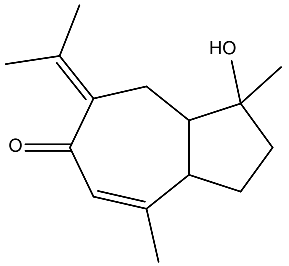

Procurcumenol

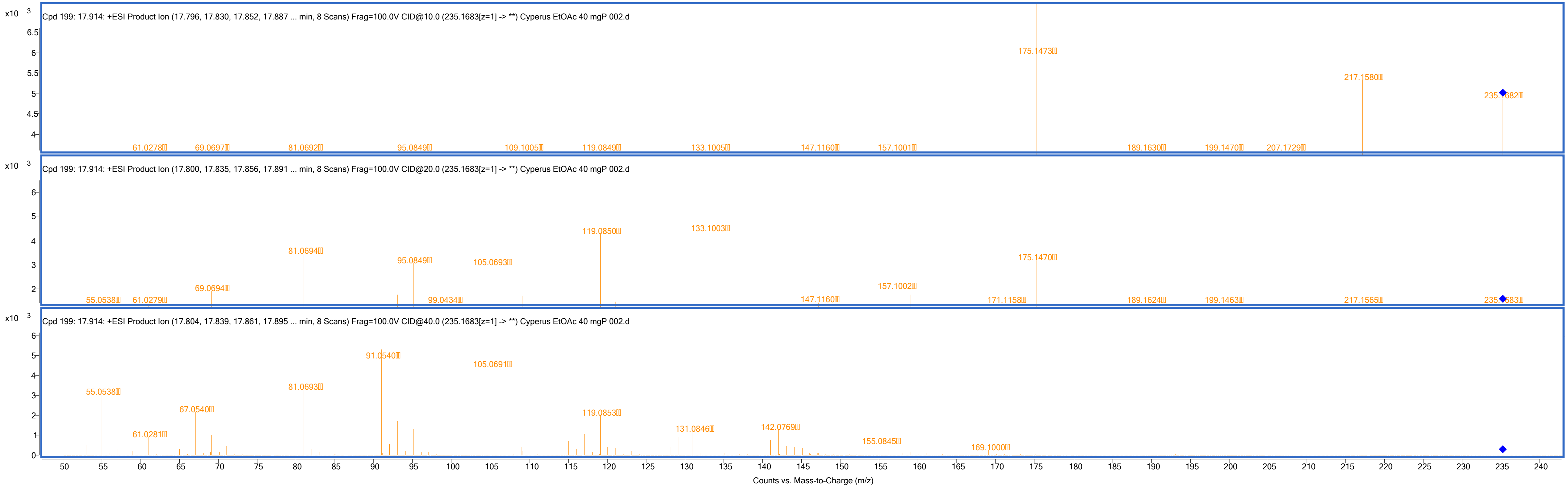

Figure S3B.27. The ESI-MS/MS fragmentation spectra of compound No 27 at m/z 235.1692 at various collision energies (10, 20, 40 eV) in the positive ionization mode.

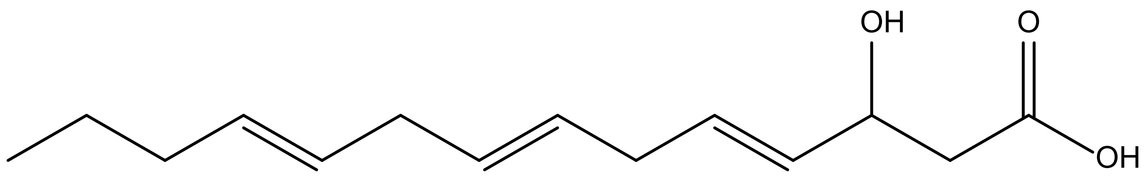

3-hydroxytetradeca-4,7,10-trienoic acid

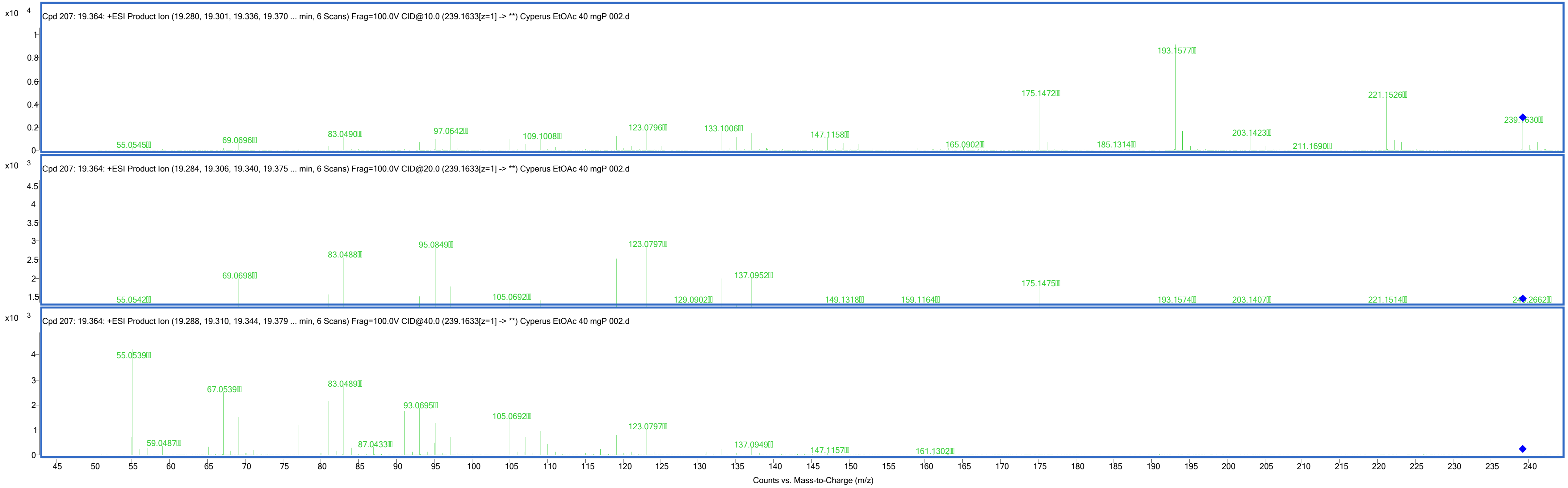

Figure S3B.28. The ESI-MS/MS fragmentation spectra of compound No 28 at m/z 239.1641 at various collision energies (10, 20, 40 eV) in the positive ionization mode.

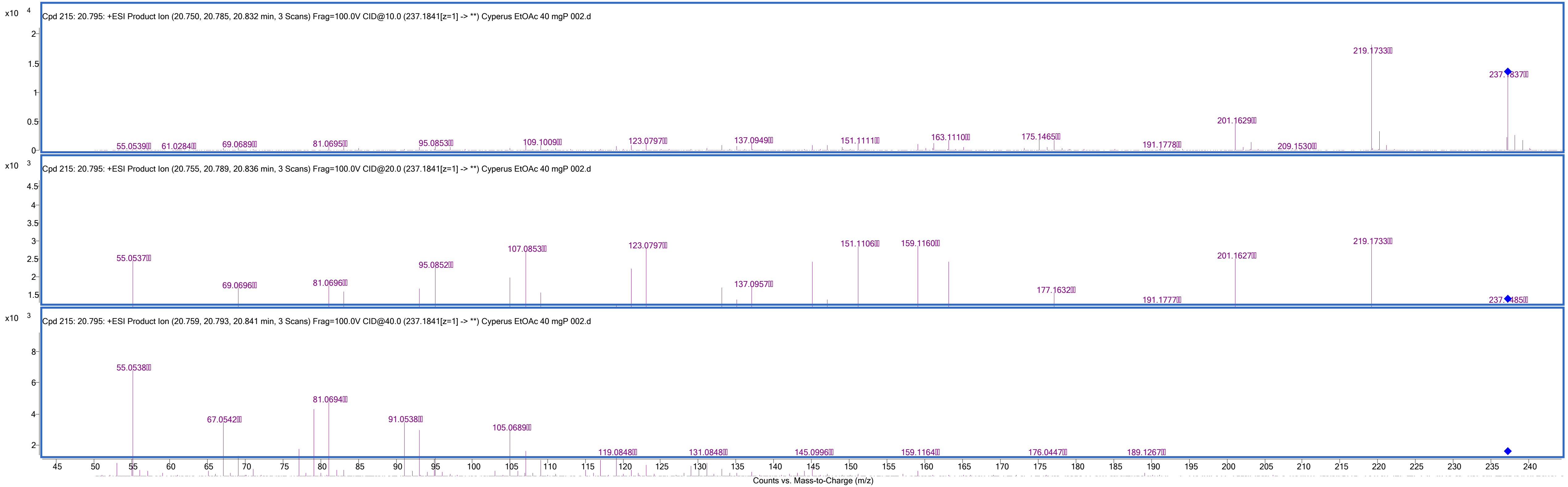

Figure S3B.29. The ESI-MS/MS fragmentation spectra of compound No 29 at m/z 237.1845 at various collision energies (10, 20, 40 eV) in the positive ionization mode.

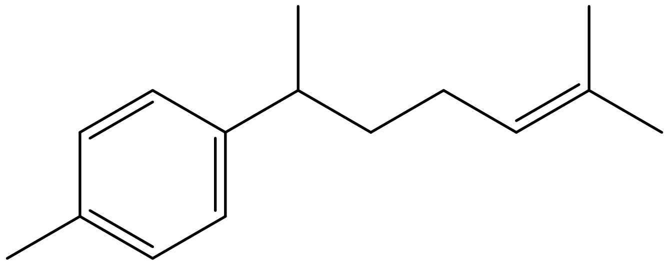

Curcumene

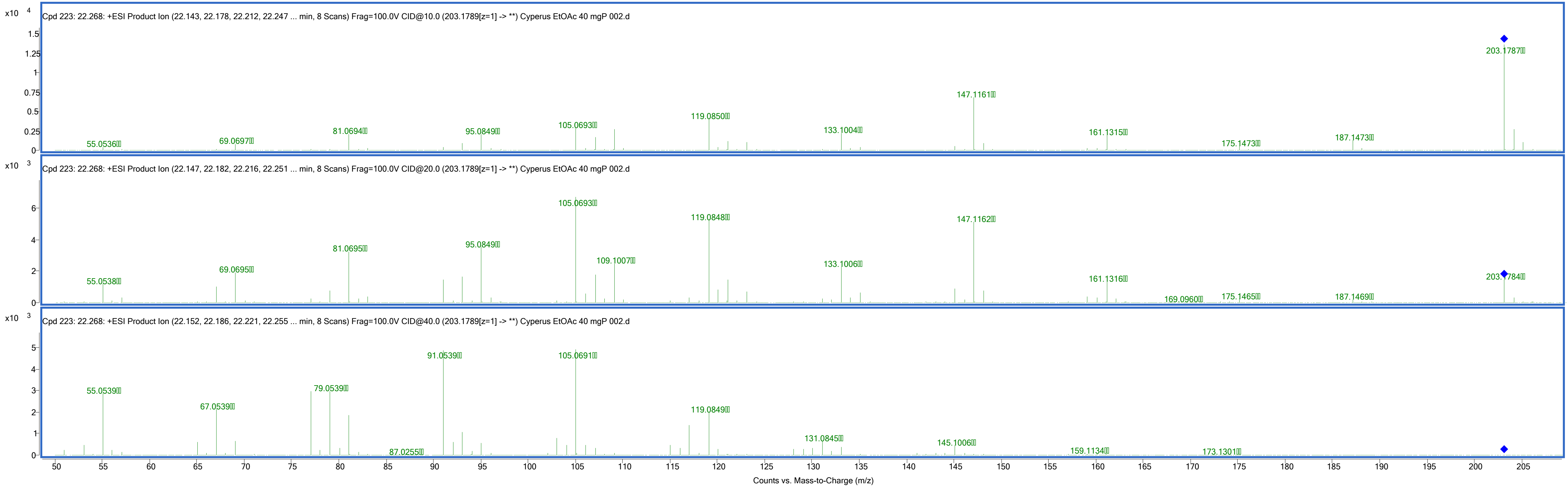

Figure S3B.30. The ESI-MS/MS fragmentation spectra of compound No 30 at m/z 203.1793 at various collision energies (10, 20, 40 eV) in the positive ionization mode.

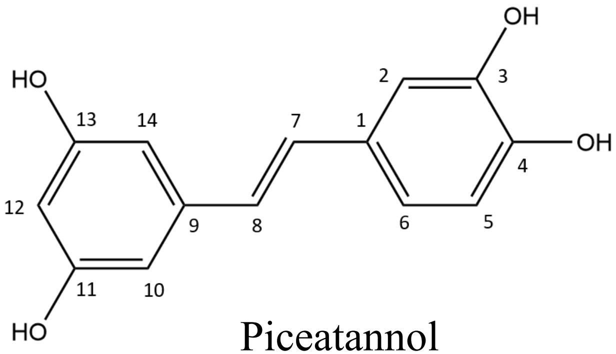

Piceatannol

$[M\text{-}H]^-$, 243.0664, Calculated for $C_{14}H_{11}O_4$, 243.0663

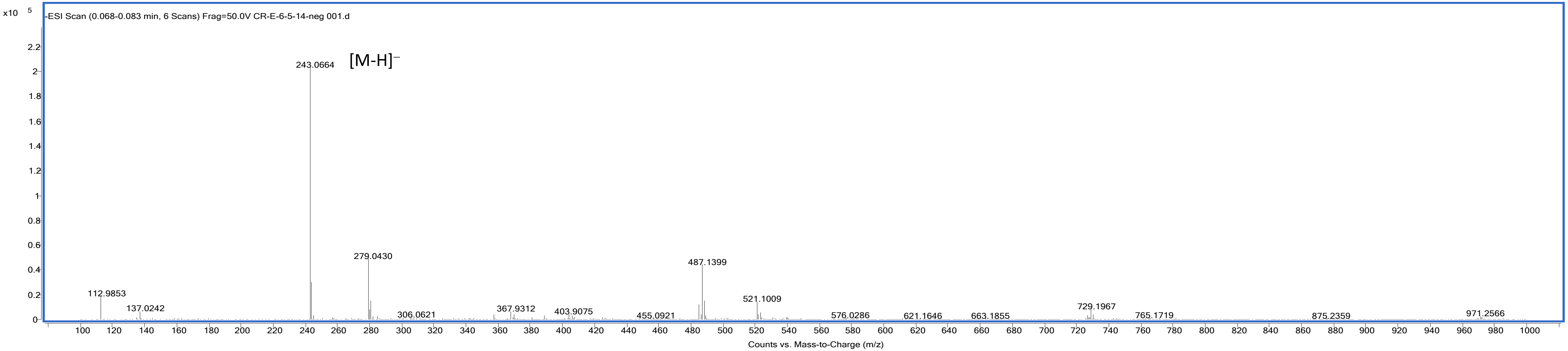

Fig. S4. The mass spectra of isolated compound CR-6-5-14 (piceatannol) operated by electrospray ionization-mass spectrometry (ESI-MS) in negative mode.

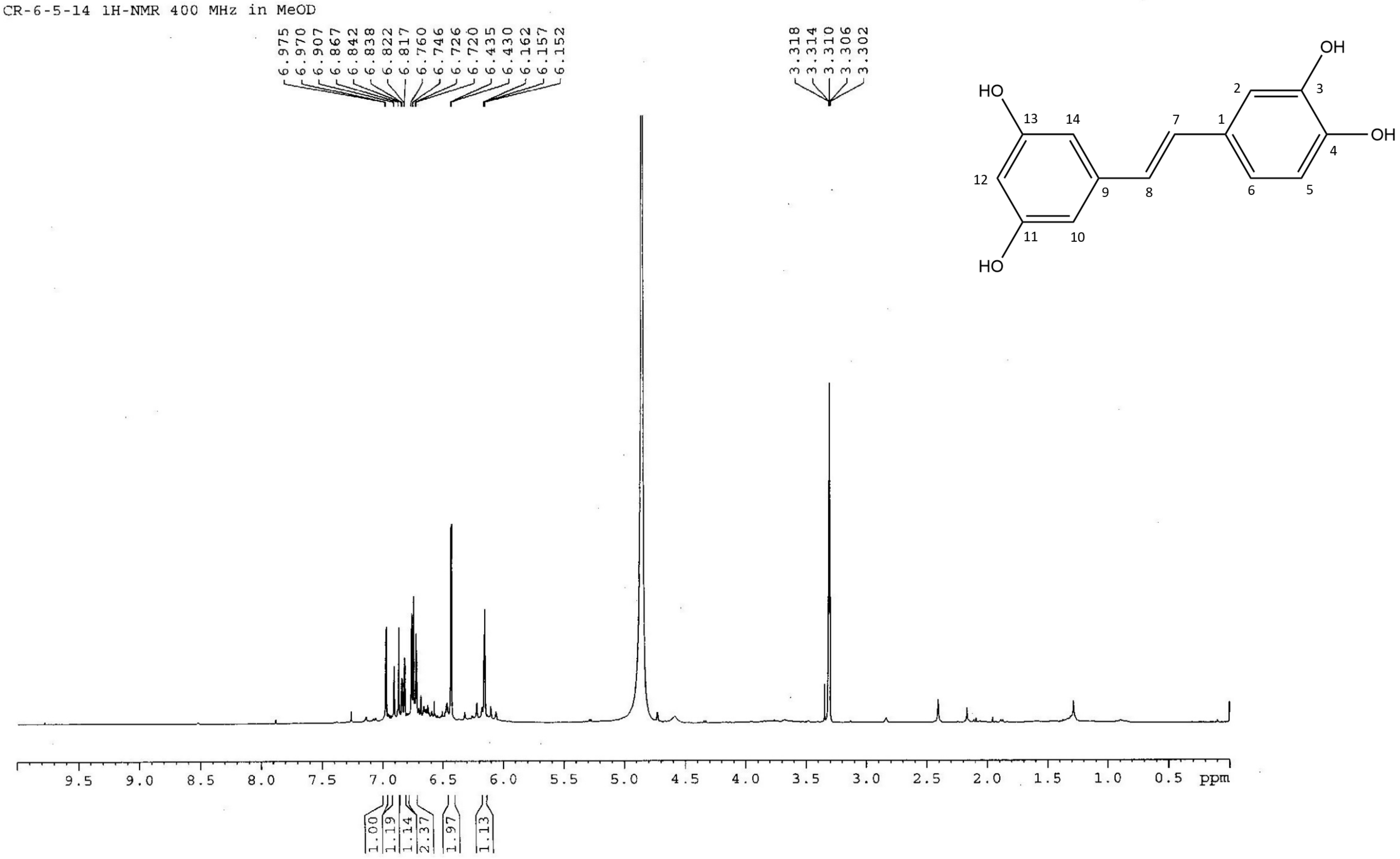

Fig. S5. The ^{1}H-NMR (400 MHz) spectra of isolated compound CR-6-5-14 (piceatannol) in CD_3OD.

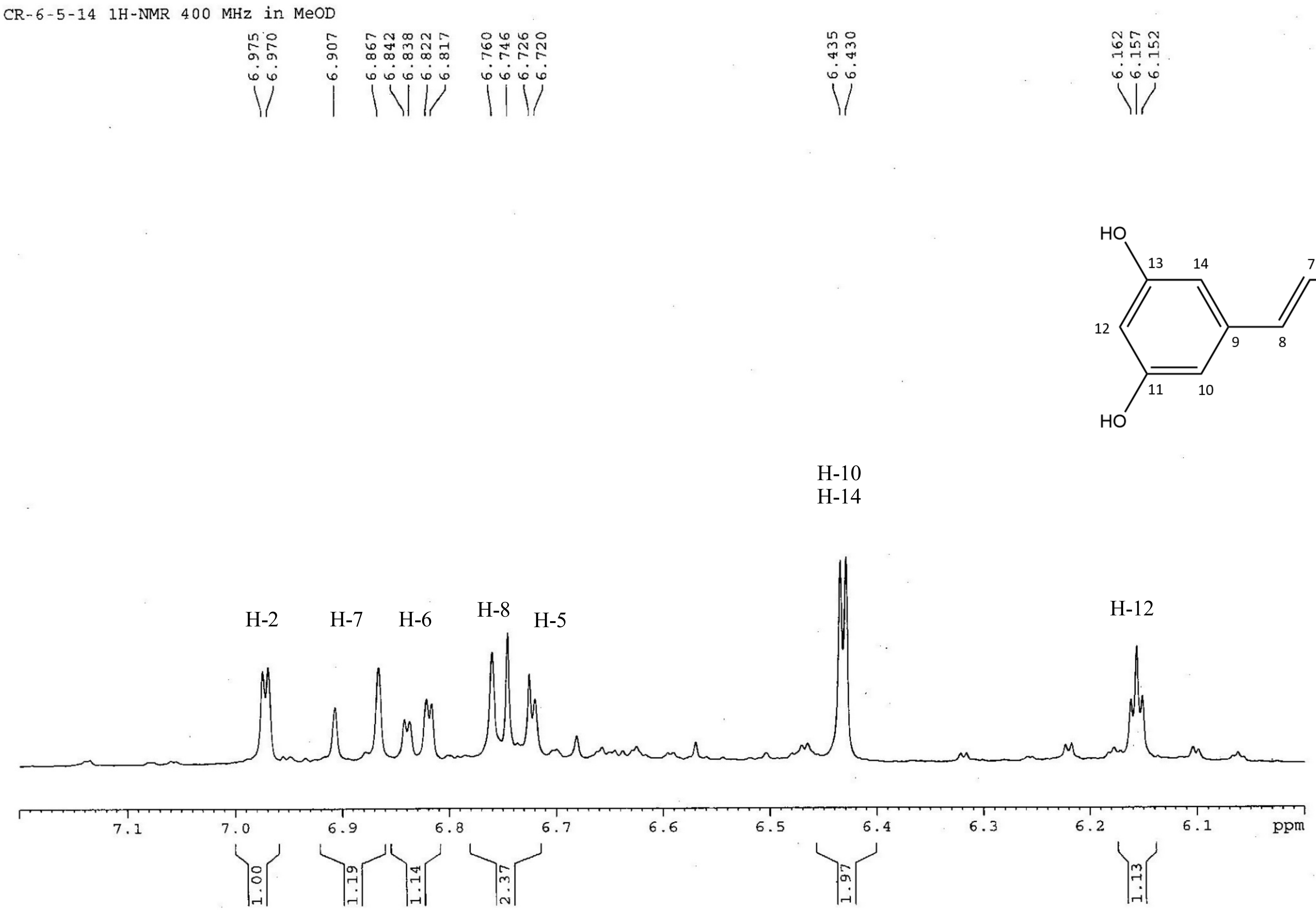

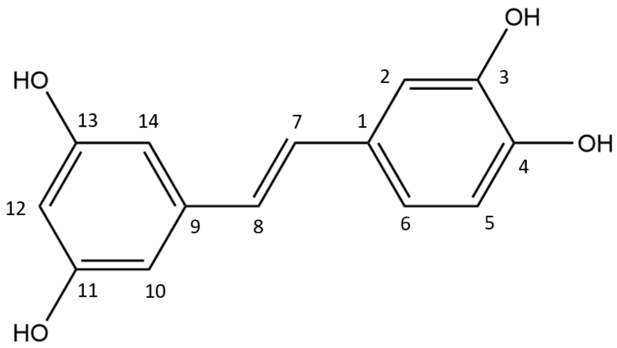

Fig. S6. The ^{1}H-NMR (400 MHz) spectra of isolated compound CR-6-5-14 (piceatannol) in CD_3OD (expanded).

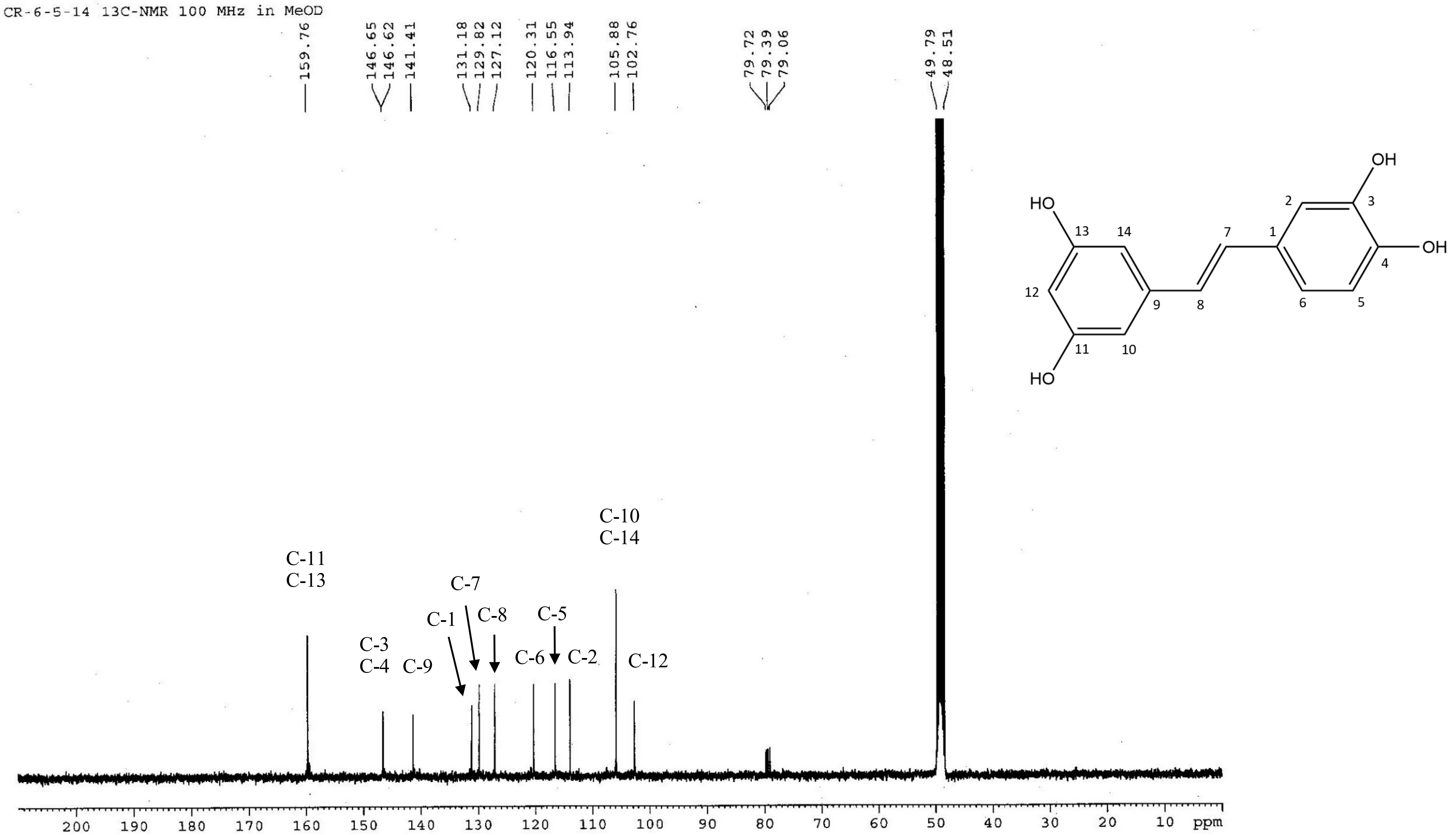

Fig. S7. The ^{13}C-NMR (100 MHz) spectra of isolated compound CR-6-5-14 (piceatannol) in CD_3OD (expanded).

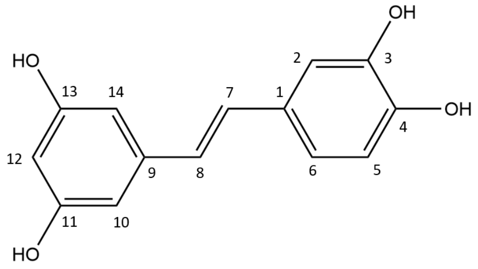

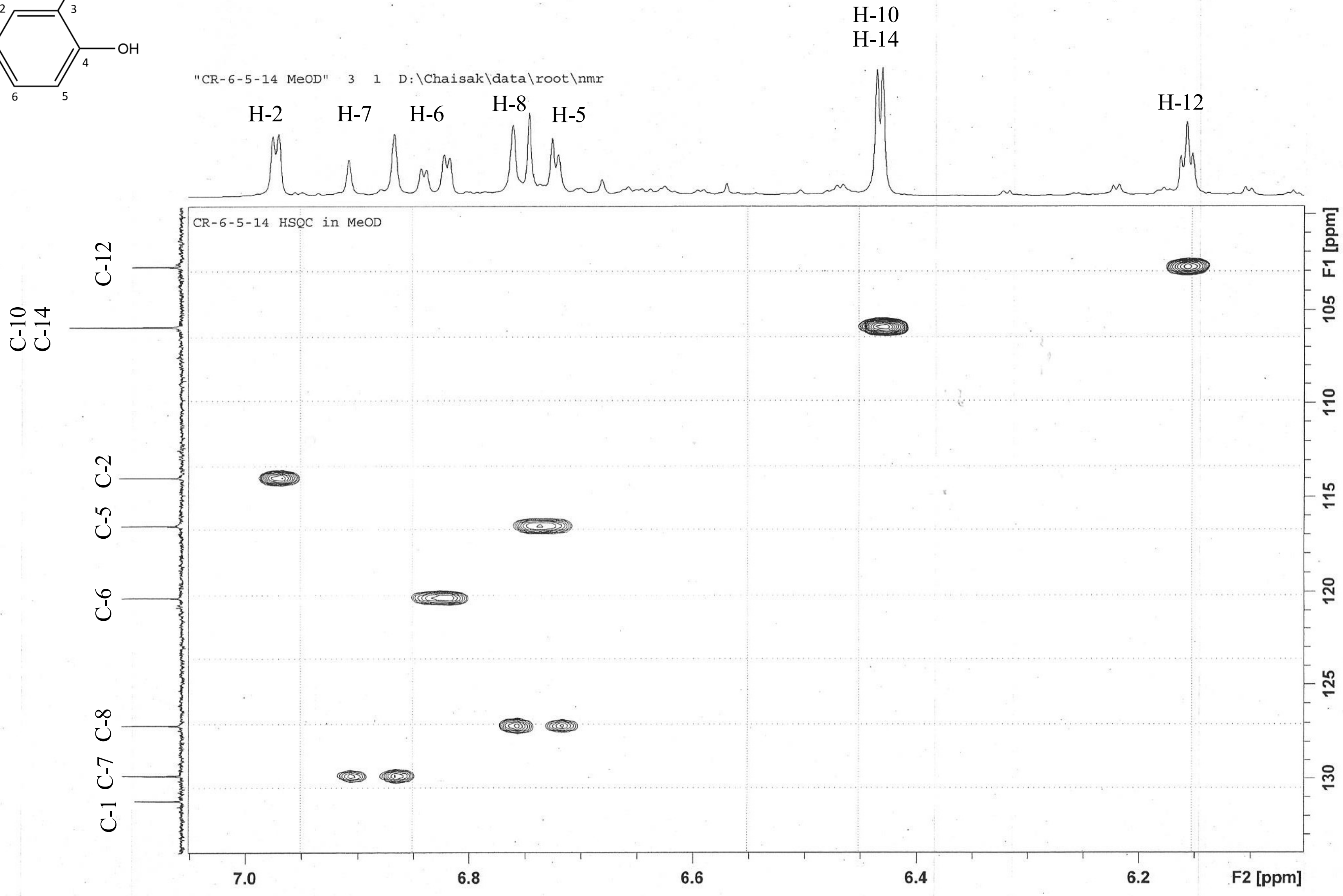

Fig. S8. The HSQC spectra of isolated compound CR-6-5-14 (piceatannol) in CD_3OD.

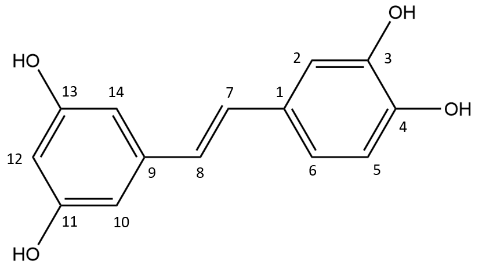

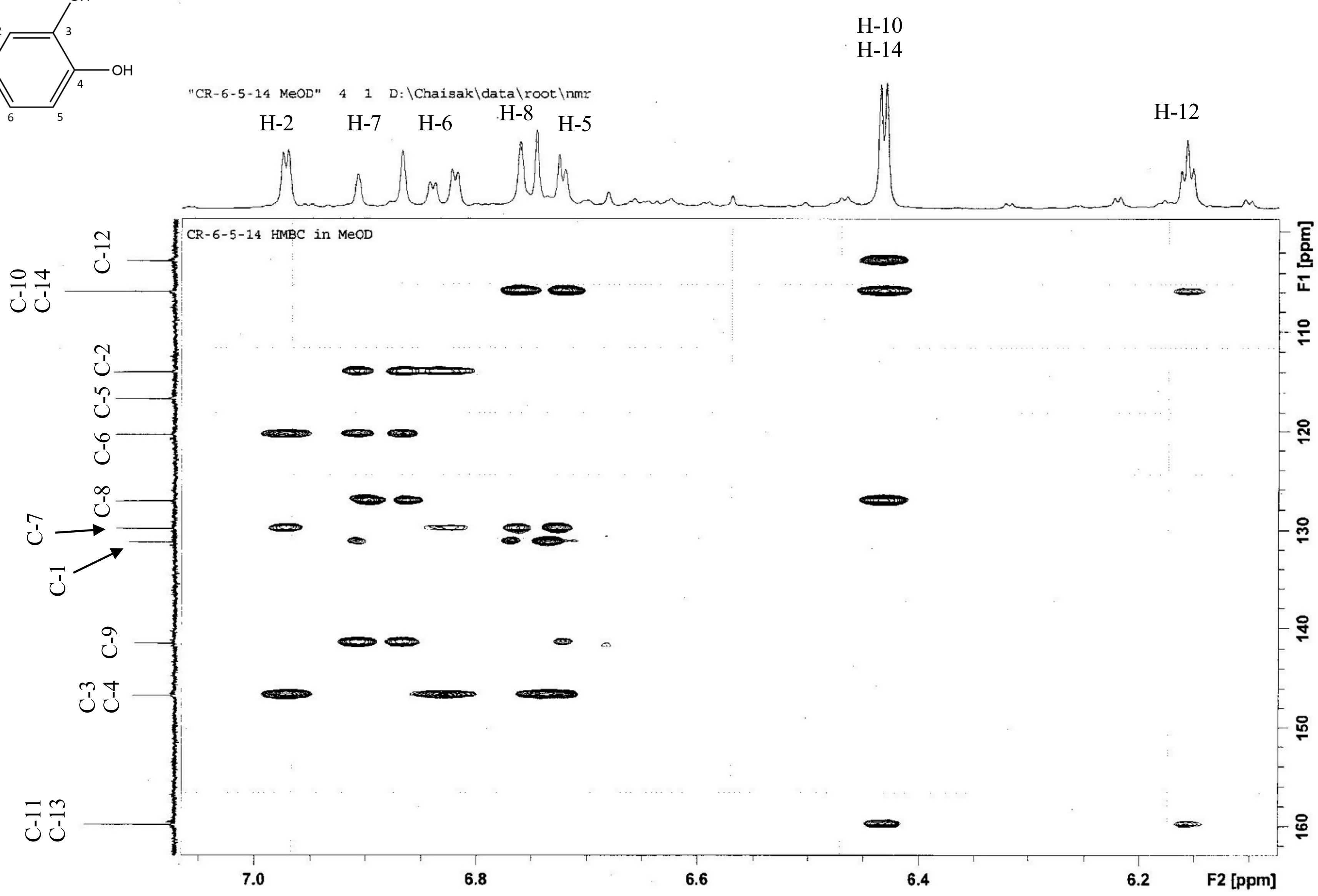

Fig. S9. The HMBC spectra of isolated compound CR-6-5-14 (piceatannol) in CD_3OD.

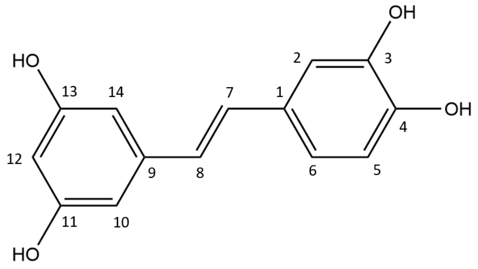

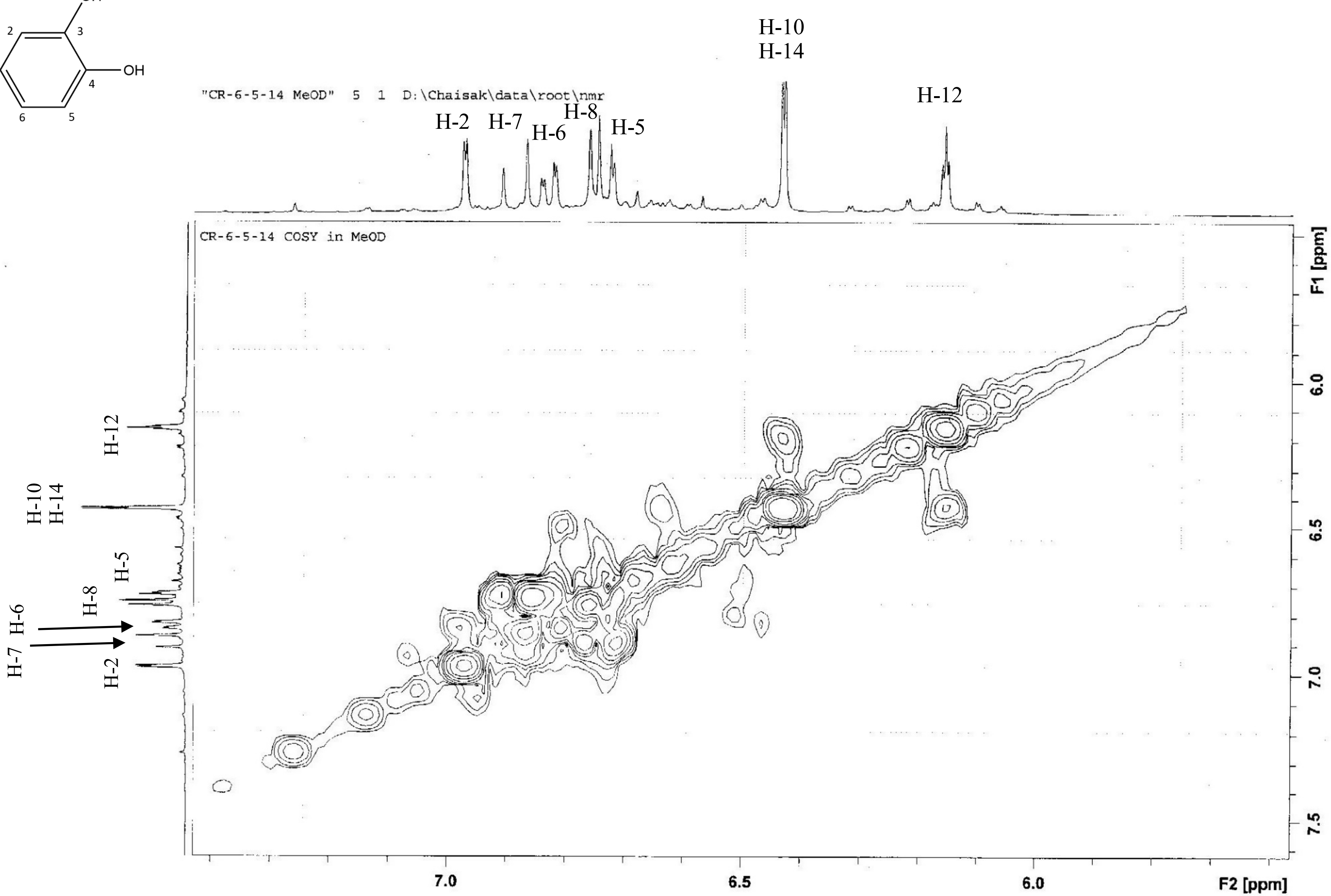

Fig. S10. The COSY spectra of isolated compound CR-6-5-14 (piceatannol) in CD_3OD.

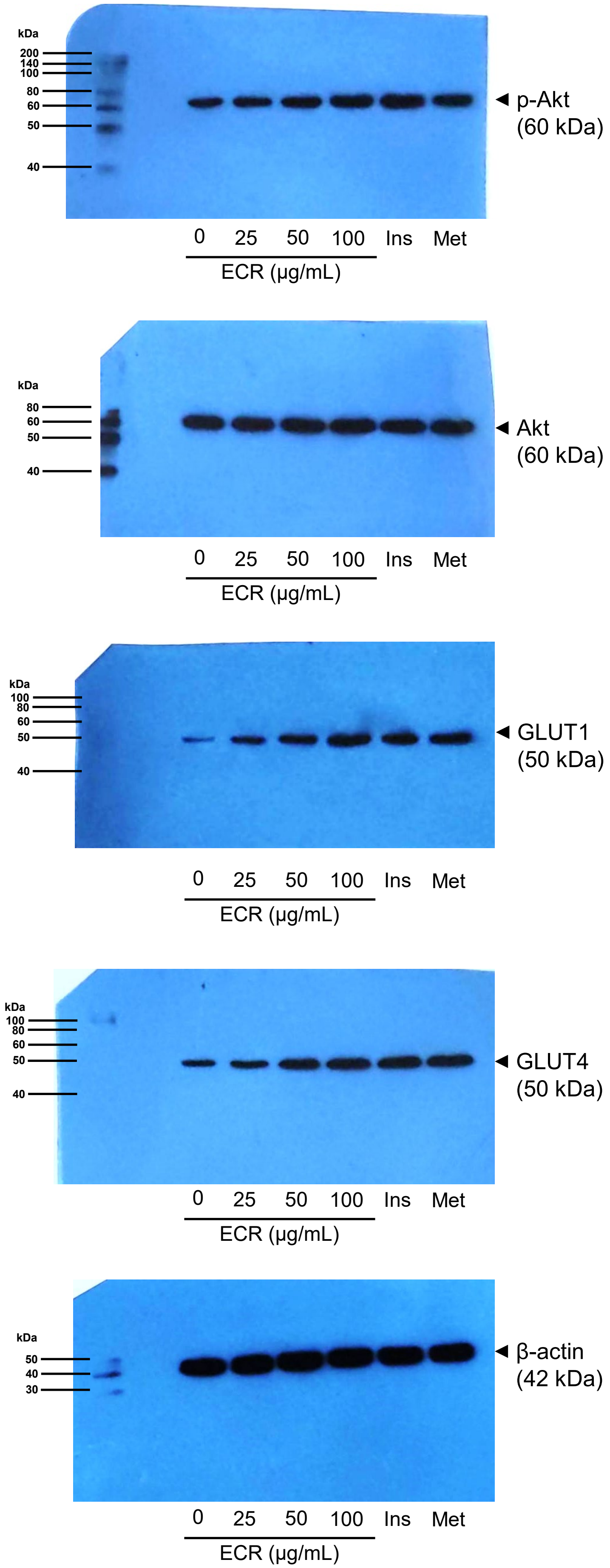

Fig. S11. Original image for Fig.3a. Effect of ECR on protein expression of glucose transporters and Akt signaling protein in L6 cells. Insulin (Ins, 100 nM) and metformin (Met, 1 mM).

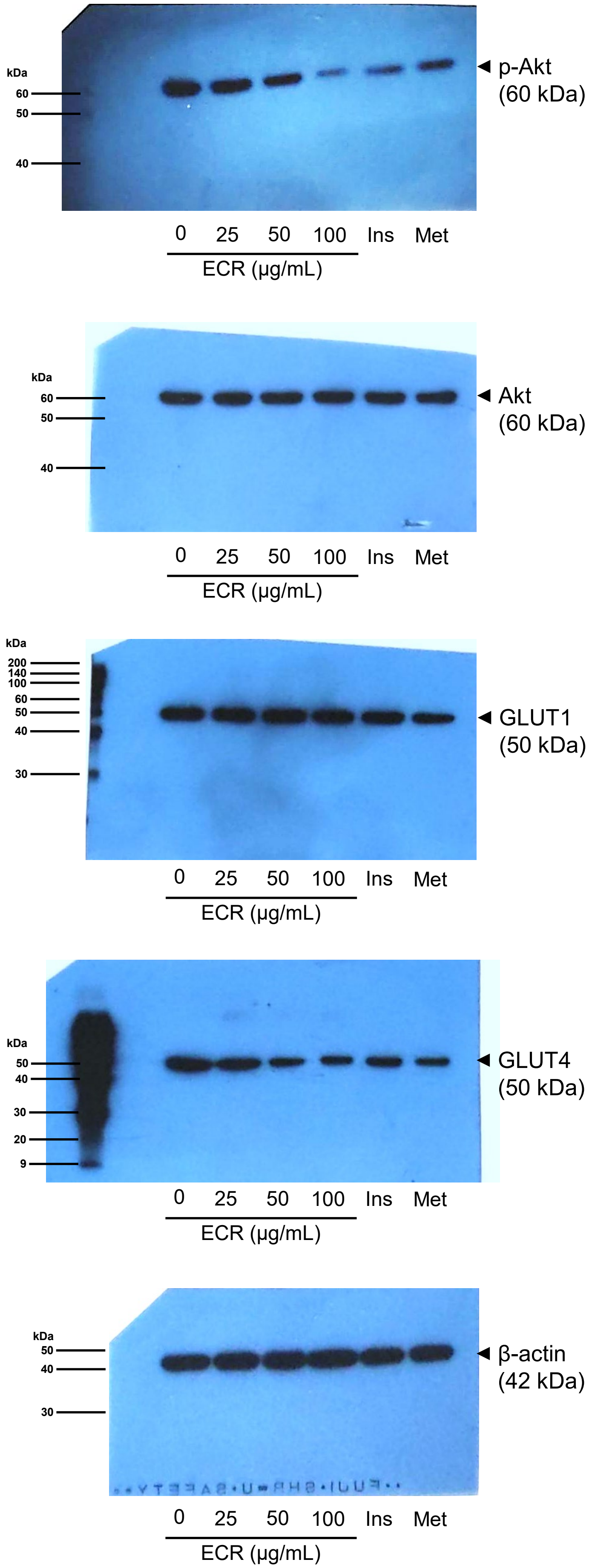

Fig. S12. Original image for Fig.3e. Effect of ECR on protein expression of glucose transporters and Akt signaling protein in 3T3-L1 cells. Insulin (Ins, 100 nM) and metformin (Met, 1 mM).

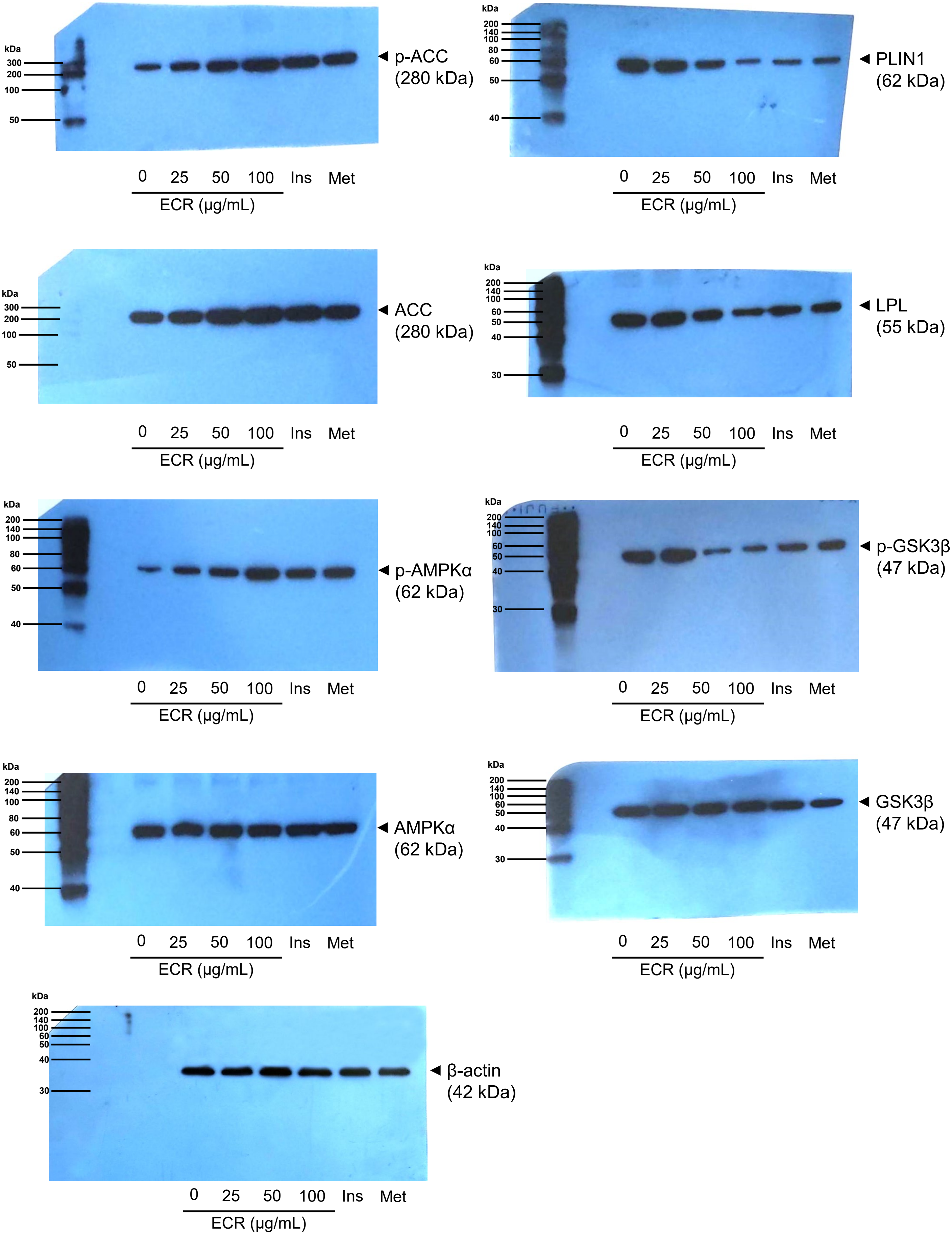

Fig. S13. Original image for Fig.6c. Suppressive effect of ECR on adipocyte maturation in 3T3-L1 cells. Insulin (Ins, 100 nM) and metformin (Met, 1 mM).
